# Supplementary material for: The association of bearing surface materials with the risk of revision following primary total hip replacement: A cohort analysis of 1,026,481 hip replacements from the National Joint Registry
Source: PLoS Med. 2024 Nov 7;21(11):e1004478. doi: 10.1371/journal.pmed.1004478 (PMC11542800; doi:10.1371/journal.pmed.1004478)
Supplement: S1 STROBE Checklist — (DOCX) [file pmed.1004478.s001.docx]

STROBE Statement—Checklist of items that should be included in reports of ***cohort studies***

|  | Item No | Recommendation | Page No |
| --- | --- | --- | --- |
| **Title and abstract** | 1 | (*a*) Indicate the study’s design with a commonly used term in the title or the abstract | Title |
|  |  | (*b*) Provide in the abstract an informative and balanced summary of what was done and what was found | Abstract |
| Introduction | | | |
| Background/rationale | 2 | Explain the scientific background and rationale for the investigation being reported | INTRODUCTION  Lines 1 to 25 and then 1 to 7 |
| Objectives | 3 | State specific objectives, including any prespecified hypotheses | End of INTRODUCTION  Lines 8-10 |
| Methods | | | |
| Study design | 4 | Present key elements of study design early in the paper | Methods, Data source lines 3-13 |
| Setting | 5 | Describe the setting, locations, and relevant dates, including periods of recruitment, exposure, follow-up, and data collection | Methods, Data source lines 3-13 |
| Participants | 6 | (*a*) Give the eligibility criteria, and the sources and methods of selection of participants. Describe methods of follow-up | Methods, Data source lines 3-13 |
|  |  | (*b*) For matched studies, give matching criteria and number of exposed and unexposed | na |
| Variables | 7 | Clearly define all outcomes, exposures, predictors, potential confounders, and effect modifiers. Give diagnostic criteria, if applicable | Methods, Outcomes and Exposure and adjustment factors |
| Data sources/ measurement | 8* | For each variable of interest, give sources of data and details of methods of assessment (measurement). Describe comparability of assessment methods if there is more than one group | Methods, Outcomes and Exposure and adjustment factors |
| Bias | 9 | Describe any efforts to address potential sources of bias | Methods, Outcomes and Exposure and adjustment factors |
| Study size | 10 | Explain how the study size was arrived at | Methods, Outcomes and Exposure and adjustment factors |
| Quantitative variables | 11 | Explain how quantitative variables were handled in the analyses. If applicable, describe which groupings were chosen and why | Methods, Statistical analysis |
| Statistical methods | 12 | (*a*) Describe all statistical methods, including those used to control for confounding | Methods, Statistical analysis |
|  |  | (*b*) Describe any methods used to examine subgroups and interactions | Methods, Statistical analysis |
|  |  | (*c*) Explain how missing data were addressed | Methods, Statistical analysis |
|  |  | (*d*) If applicable, explain how loss to follow-up was addressed | na |
|  |  | (*e*) Describe any sensitivity analyses | na |
| Results | | |  |
| Participants | 13* | (a) Report numbers of individuals at each stage of study—eg numbers potentially eligible, examined for eligibility, confirmed eligible, included in the study, completing follow-up, and analysed | Table 1, Results lines 2-4 |
|  |  | (b) Give reasons for non-participation at each stage | sFig1 |
|  |  | (c) Consider use of a flow diagram | sFig1 |
| Descriptive data | 14* | (a) Give characteristics of study participants (eg demographic, clinical, social) and information on exposures and potential confounders | Tab1, sTab1-8 |
|  |  | (b) Indicate number of participants with missing data for each variable of interest | na |
|  |  | (c) Summarise follow-up time (eg, average and total amount) | Results lines 2-4 |
| Outcome data | 15* | Report numbers of outcome events or summary measures over time | Results Monobloc acetabular implants and Results Modular acetabular implants |

| Main results | 16 | (*a*) Give unadjusted estimates and, if applicable, confounder-adjusted estimates and their precision (eg, 95% confidence interval). Make clear which confounders were adjusted for and why they were included | Results Monobloc acetabular implants and Results Modular acetabular implants, Tabs2-3, Fig 1-2, sTab9-26, sFig2-18 |
| --- | --- | --- | --- |
|  |  | (*b*) Report category boundaries when continuous variables were categorized | Results Monobloc acetabular implants and Results Modular acetabular implants, Tabs2-3, Fig 1-2, sTab9-26, sFig2-18 |
|  |  | (*c*) If relevant, consider translating estimates of relative risk into absolute risk for a meaningful time period | na |
| Other analyses | 17 | Report other analyses done—eg analyses of subgroups and interactions, and sensitivity analyses | na |
| Discussion | | | |
| Key results | 18 | Summarise key results with reference to study objectives | Discussion 2-25 |
| Limitations | 19 | Discuss limitations of the study, taking into account sources of potential bias or imprecision. Discuss both direction and magnitude of any potential bias | Discussion lines 13-26+lines 1-9 on following page. |
| Interpretation | 20 | Give a cautious overall interpretation of results considering objectives, limitations, multiplicity of analyses, results from similar studies, and other relevant evidence | Discussion lines 10-25+lines 1-26 on following page+liens 1-12 following page |
| Generalisability | 21 | Discuss the generalisability (external validity) of the study results | Discussion, last paragraph: lines 10-15 |
| Other information | | | |
| Funding | 22 | Give the source of funding and the role of the funders for the present study and, if applicable, for the original study on which the present article is based | Financial Disclosure Statement, lines 7-12 |

*Give information separately for exposed and unexposed groups.

**Note:** An Explanation and Elaboration article discusses each checklist item and gives methodological background and published examples of transparent reporting. The STROBE checklist is best used in conjunction with this article (freely available on the Web sites of PLoS Medicine at http://www.plosmedicine.org/, Annals of Internal Medicine at http://www.annals.org/, and Epidemiology at http://www.epidem.com/). Information on the STROBE Initiative is available at http://www.strobe-statement.org.
