# Supplementary material for: The association of bearing surface materials with the risk of revision following primary total hip replacement: A cohort analysis of 1,026,481 hip replacements from the National Joint Registry
Source: PLoS Med. 2024 Nov 7;21(11):e1004478. doi: 10.1371/journal.pmed.1004478 (PMC11542800; doi:10.1371/journal.pmed.1004478)
Supplement: S1 Text — Including: Table A. Description of the studied sample and revision rate for aseptic loosening. Table B. Description of the studied sample and revision rate for peri-prosthetic fracture. Table C. Description of the studied sample and revision rate for implant wear. Table D. Description of the studied sample and revision rate for malalignment. Table E. Description of the studied sample and revision rate for dislocation or subluxation. Table F. Description of the studied sample and revision rate for pain. Table G. Description of the studied sample and revision rate for infection. Table H. Description of the studied sample and revision rate for any other reason(s). Table I. Monobloc acetabular component—all-cause revision hazard ratio (HR) and 95% confidence interval (CI) by time point from primary procedure-Reference: Implant with cobalt chrome head and highly crosslinked polyethylene cup. Table J. Monobloc acetabular component-Revision for aseptic loosening hazard ratio (HR) and 95% confidence interval (CI) by time point from primary procedure-Reference: Implant with cobalt chrome head and highly crosslinked polyethylene cup. Table K. Monobloc acetabular component-Revision for peri-prosthetic fracture hazard ratio (HR) and 95% confidence interval (CI) by time point from primary procedure-Reference: Implant with cobalt chrome head and highly crosslinked polyethylene cup. Table L. Monobloc acetabular component-Revision for implant wear hazard ratio (HR) and 95% confidence interval (CI) by time point from primary procedure-Reference: Implant with cobalt chrome head and highly crosslinked polyethylene cup. Table M. Monobloc acetabular component-Revision for malalignment hazard ratio (HR) and 95% confidence interval (CI) by time point from primary procedure-Reference: Implant with cobalt chrome head and highly crosslinked polyethylene cup. Table N. Monobloc acetabular component-Revision for dislocation or subluxation hazard ratio (HR) and 95% confidence interval (CI) by ti [file pmed.1004478.s002.docx]

# **Supplementary**

## **Supplementary 1: Description of the sample by specific indication for revision**

**Table A:** Description of the studied sample and revision rate for aseptic loosening

|  |  | **Monobloc** |  |  | **Modular** |  |  |
| --- | --- | --- | --- | --- | --- | --- | --- |
|  |  | **N** | **Revision(n)** | **Revision (per 10,000)** | **N** | **Revision(n)** | **Revision (per 10,000)** |
| **Year of primary surgery** | **<2010** | 111781 | 1212 | 108.4 | 111028 | 1066 | 96.0 |
|  | **2010-2014** | 127413 | 477 | 37.4 | 228155 | 990 | 43.4 |
|  | **2015-219** | 139703 | 136 | 9.7 | 309018 | 379 | 12.3 |
| **Gender** | **Female** | 251218 | 1464 | 58.3 | 377914 | 1513 | 40.0 |
|  | **Male** | 127679 | 834 | 65.3 | 270287 | 1530 | 56.6 |
| **Age at primary (years)** | **<55** | 14368 | 185 | 128.8 | 93190 | 697 | 74.8 |
|  | **55 to 64** | 48592 | 572 | 117.7 | 165336 | 1010 | 61.1 |
|  | **65 to 74** | 141877 | 1087 | 76.6 | 231077 | 941 | 40.7 |
|  | **>=75** | 174060 | 454 | 26.1 | 158598 | 395 | 24.9 |
| **Body Mass Index at primary** | **<18.5** | 2658 | 6 | 22.6 | 3682 | 12 | 32.6 |
|  | **[18.5-24.9]** | 51097 | 204 | 39.9 | 87542 | 271 | 31.0 |
|  | **[25-29.9]** | 92193 | 347 | 37.6 | 172498 | 606 | 35.1 |
|  | **>29.9** | 84542 | 355 | 42.0 | 177560 | 755 | 42.5 |
|  | **Unknown BMI** | 148407 | 1386 | 93.4 | 206919 | 1399 | 67.6 |
| **ASA grade** | **P1 - Fit and healthy** | 40275 | 424 | 105.3 | 104981 | 665 | 63.3 |
|  | **P2 - Mild disease not incapacitating** | 259297 | 1565 | 60.4 | 446785 | 2020 | 45.2 |
|  | **P3 - Incapacitating systemic disease** | 76495 | 304 | 39.7 | 93486 | 352 | 37.7 |
|  | **P4 - Life threatening disease** | 2783 | 5 | 18.0 | 2901 | 5 | 17.2 |
|  | **P5 - Expected to die within 24hrs with or without an operation** | 47 | 0 | 0.0 | 48 | 1 | 208.3 |
| **Fixation type** | **Cemented** | 345036 | 2057 | 59.6 | 674 | 8 | 118.7 |
|  | **Hybrid** | 9 | 0 | 0.0 | 242042 | 540 | 22.3 |
|  | **Reverse hybrid** | 30394 | 199 | 65.5 | 89 | 5 | 561.8 |
|  | **Uncemented** | 35 | 0 | 0.0 | 395493 | 2397 | 60.6 |
|  | **Unclassified** | 3423 | 42 | 122.7 | 9903 | 93 | 93.9 |
| **Stem composition** | **Stainless Steel** | 289364 | 1503 | 51.9 | 178564 | 373 | 20.9 |
|  | **Cobalt chrome** | 54909 | 528 | 96.2 | 64626 | 182 | 28.2 |
|  | **Titanium** | 30680 | 218 | 71.1 | 391672 | 2359 | 60.2 |
|  | **Other or unknown** | 3944 | 49 | 124.2 | 13339 | 129 | 96.7 |
| **Head size (mm)** | **22.25** | 12893 | 119 | 92.3 | 1021 | 10 | 97.9 |
|  | **26** | 18694 | 191 | 102.2 | 775 | 6 | 77.4 |
|  | **28** | 241452 | 1637 | 67.8 | 144874 | 1053 | 72.7 |
|  | **30-32** | 93203 | 283 | 30.4 | 268385 | 809 | 30.1 |
|  | **>=36** | 9232 | 26 | 28.2 | 223243 | 1072 | 48.0 |
|  | **Unknown** | 3423 | 42 | 122.7 | 9903 | 93 | 93.9 |
| **Head Cup** | **Head: Alumina, Cup: HCLPE** | 6621 | 18 | 27.2 |  |  |  |
|  | **Head: Alumina, Cup: non-HCLPE** | 12255 | 107 | 87.3 |  |  |  |
|  | **Head: Cobalt Chrome, Cup: HCLPE** | 35069 | 81 | 23.1 |  |  |  |
|  | **Head: Cobalt Chrome,, Cup: non-HCLPE** | 69075 | 589 | 85.3 |  |  |  |
|  | **Head: Delta Ceramic, Cup: HCLPE** | 23279 | 56 | 24.1 |  |  |  |
|  | **Head: Delta Ceramic, Cup: non-HCLPE** | 14064 | 101 | 71.8 |  |  |  |
|  | **Head: Stainless Steel, Cup: HCLPE** | 35825 | 58 | 16.2 |  |  |  |
|  | **Head: Stainless Steel, Cup: non-HCLPE** | 182709 | 1288 | 70.5 |  |  |  |
| **Shell composition** | **Cobalt Chrome** |  |  |  | 720 | 11 | 152.8 |
|  | **Stainless Steel** |  |  |  | 5273 | 45 | 85.3 |
|  | **Tantalum** |  |  |  | 5741 | 36 | 62.7 |
|  | **Titanium** |  |  |  | 629205 | 2908 | 46.2 |
|  | **Unknown** |  |  |  | 7262 | 43 | 59.2 |
| **Head Liner** | **Head: Alumina, Liner: Alumina** |  |  |  | 27602 | 248 | 89.8 |
|  | **Head: Alumina, Liner: HCLPE** |  |  |  | 25191 | 75 | 29.8 |
|  | **Head: Alumina, Liner: non-HCLPE** |  |  |  | 3281 | 49 | 149.3 |
|  | **Head: Cobalt Chrome, Liner: HCLPE** |  |  |  | 183106 | 579 | 31.6 |
|  | **Head: Cobalt Chrome, Liner: non-HCLPE** |  |  |  | 42389 | 594 | 140.1 |
|  | **Head: Delta Ceramic, Liner: Delta Ceramic** |  |  |  | 119558 | 847 | 70.8 |
|  | **Head: Delta Ceramic, Liner: HCLPE** |  |  |  | 140539 | 257 | 18.3 |
|  | **Head: Delta Ceramic, Liner: non-HCLPE** |  |  |  | 13448 | 109 | 81.1 |
|  | **Head: Delta Ceramic, Pre-assembled implant** |  |  |  | 4614 | 21 | 45.5 |
|  | **Head: Other head materials, Pre-assembled implant** |  |  |  | 2648 | 22 | 83.1 |
|  | **Head: Oxidised, Liner: HCLPE** |  |  |  | 21263 | 35 | 16.5 |
|  | **Head: Stainless Steel, Liner: HCLPE** |  |  |  | 45672 | 44 | 9.6 |
|  | **Head: Stainless Steel, Liner: non-HCLPE** |  |  |  | 18890 | 163 | 86.3 |

ASA: American Society Anaesthesiologists Physical Status Classification

HCLPE: Highly Cross-Linked Polyethylene

**Table B:** Description of the studied sample and revision rate for peri-prosthetic fracture

|  |  | **Monobloc** |  |  | **Modular** |  |  |
| --- | --- | --- | --- | --- | --- | --- | --- |
|  |  | **N** | **Revision(n)** | **Revision(per 10,000)** | **N** | **Revision(n)** | **Revision(per 10,000)** |
| **Year of primary surgery** | **<2010** | 111781 | 408 | 36.5 | 111028 | 594 | 53.5 |
|  | **2010-2014** | 127413 | 355 | 27.9 | 228155 | 858 | 37.6 |
|  | **2015-219** | 139703 | 218 | 15.6 | 309018 | 605 | 19.6 |
| **Gender** | **Female** | 251218 | 587 | 23.4 | 377914 | 1504 | 39.8 |
|  | **Male** | 127679 | 652 | 51.1 | 270287 | 1064 | 39.4 |
| **Age at primary (years)** | **<55** | 14368 | 47 | 32.7 | 93190 | 214 | 23.0 |
|  | **55 to 64** | 48592 | 128 | 26.3 | 165336 | 481 | 29.1 |
|  | **65 to 74** | 141877 | 467 | 32.9 | 231077 | 910 | 39.4 |
|  | **>=75** | 174060 | 597 | 34.3 | 158598 | 963 | 60.7 |
| **Body Mass Index at primary** | **<18.5** | 2658 | 8 | 30.1 | 3682 | 20 | 54.3 |
|  | **[18.5-24.9]** | 51097 | 151 | 29.5 | 87542 | 369 | 42.2 |
|  | **[25-29.9]** | 92193 | 253 | 27.4 | 172498 | 571 | 33.1 |
|  | **>29.9** | 84542 | 237 | 28.0 | 177560 | 599 | 33.7 |
|  | **Unknown BMI** | 148407 | 590 | 39.8 | 206919 | 1009 | 48.8 |
| **ASA grade** | **P1 - Fit and healthy** | 40275 | 118 | 29.3 | 104981 | 312 | 29.7 |
|  | **P2 - Mild disease not incapacitating** | 259297 | 851 | 32.8 | 446785 | 1748 | 39.1 |
|  | **P3 - Incapacitating systemic disease** | 76495 | 263 | 34.4 | 93486 | 495 | 53.0 |
|  | **P4 - Life threatening disease** | 2783 | 7 | 25.1 | 2901 | 13 | 44.8 |
|  | **P5 - Expected to die within 24hrs with or without an operation** | 47 | 0 | 0.0 | 48 | 0 | 0.0 |
| **Fixation type** | **Cemented** | 345036 | 1117 | 32.4 | 674 | 4 | 59.3 |
|  | **Hybrid** | 9 | 0 | 0.0 | 242042 | 1011 | 41.8 |
|  | **Reverse hybrid** | 30394 | 109 | 35.9 | 89 | 0 | 0.0 |
|  | **Uncemented** | 35 | 0 | 0.0 | 395493 | 1477 | 37.3 |
|  | **Unclassified** | 3423 | 13 | 38.0 | 9903 | 76 | 76.7 |
| **Stem composition** | **Stainless Steel** | 289364 | 845 | 29.2 | 178564 | 549 | 30.8 |
|  | **Cobalt chrome** | 54909 | 267 | 48.6 | 64626 | 468 | 72.4 |
|  | **Titanium** | 30680 | 113 | 36.8 | 391672 | 1451 | 37.0 |
|  | **Other or unknown** | 3944 | 14 | 35.5 | 13339 | 100 | 75.0 |
| **Head size (mm)** | **22.25** | 12893 | 37 | 28.7 | 1021 | 4 | 39.2 |
|  | **26** | 18694 | 88 | 47.1 | 775 | 1 | 12.9 |
|  | **28** | 241452 | 760 | 31.5 | 144874 | 660 | 45.6 |
|  | **30-32** | 93203 | 296 | 31.8 | 268385 | 887 | 33.0 |
|  | **>=36** | 9232 | 45 | 48.7 | 223243 | 940 | 42.1 |
|  | **Unknown** | 3423 | 13 | 38.0 | 9903 | 76 | 76.7 |
| **Head Cup** | **Head: Alumina, Cup: HCLPE** | 6621 | 14 | 21.1 |  |  |  |
|  | **Head: Alumina, Cup: non-HCLPE** | 12255 | 28 | 22.9 |  |  |  |
|  | **Head: Cobalt Chrome, Cup: HCLPE** | 35069 | 117 | 33.4 |  |  |  |
|  | **Head: Cobalt Chrome,, Cup: non-HCLPE** | 69075 | 372 | 53.8 |  |  |  |
|  | **Head: Delta Ceramic, Cup: HCLPE** | 23279 | 45 | 19.3 |  |  |  |
|  | **Head: Delta Ceramic, Cup: non-HCLPE** | 14064 | 31 | 22.0 |  |  |  |
|  | **Head: Stainless Steel, Cup: HCLPE** | 35825 | 72 | 20.1 |  |  |  |
|  | **Head: Stainless Steel, Cup: non-HCLPE** | 182709 | 560 | 30.6 |  |  |  |
| **Shell composition** | **Cobalt Chrome** |  |  |  | 720 | 5 | 69.4 |
|  | **Stainless Steel** |  |  |  | 5273 | 38 | 72.1 |
|  | **Tantalum** |  |  |  | 5741 | 33 | 57.5 |
|  | **Titanium** |  |  |  | 629205 | 2468 | 39.2 |
|  | **Unknown** |  |  |  | 7262 | 24 | 33.0 |
| **Head Liner** | **Head: Alumina, Liner: Alumina** |  |  |  | 27602 | 133 | 48.2 |
|  | **Head: Alumina, Liner: HCLPE** |  |  |  | 25191 | 72 | 28.6 |
|  | **Head: Alumina, Liner: non-HCLPE** |  |  |  | 3281 | 20 | 61.0 |
|  | **Head: Cobalt Chrome, Liner: HCLPE** |  |  |  | 183106 | 920 | 50.2 |
|  | **Head: Cobalt Chrome, Liner: non-HCLPE** |  |  |  | 42389 | 322 | 76.0 |
|  | **Head: Delta Ceramic, Liner: Delta Ceramic** |  |  |  | 119558 | 392 | 32.8 |
|  | **Head: Delta Ceramic, Liner: HCLPE** |  |  |  | 140539 | 352 | 25.0 |
|  | **Head: Delta Ceramic, Liner: non-HCLPE** |  |  |  | 13448 | 39 | 29.0 |
|  | **Head: Delta Ceramic, Pre-assembled implant** |  |  |  | 4614 | 10 | 21.7 |
|  | **Head: Other head materials, Pre-assembled implant** |  |  |  | 2648 | 14 | 52.9 |
|  | **Head: Oxidised, Liner: HCLPE** |  |  |  | 21263 | 55 | 25.9 |
|  | **Head: Stainless Steel, Liner: HCLPE** |  |  |  | 45672 | 123 | 26.9 |
|  | **Head: Stainless Steel, Liner: non-HCLPE** |  |  |  | 18890 | 116 | 61.4 |

ASA: American Society Anaesthesiologists Physical Status Classification

HCLPE: Highly Cross-Linked Polyethylene

**Table C:** Description of the studied sample and revision rate for implant wear

|  |  | **Monobloc** |  |  | **Modular** |  |  |
| --- | --- | --- | --- | --- | --- | --- | --- |
|  |  | **N** | **Revision(n)** | **Revision(per 10,000)** | **N** | **Revision(n)** | **Revision(per 10,000)** |
| **Year of primary surgery** | **<2010** | 111781 | 218 | 19.5 | 111028 | 412 | 37.1 |
|  | **2010-2014** | 127413 | 68 | 5.3 | 228155 | 264 | 11.6 |
|  | **2015-219** | 139703 | 34 | 2.4 | 309018 | 100 | 3.2 |
| **Gender** | **Female** | 251218 | 262 | 10.4 | 377914 | 594 | 15.7 |
|  | **Male** | 127679 | 141 | 11.0 | 270287 | 374 | 13.8 |
| **Age at primary (years)** | **<55** | 14368 | 17 | 11.8 | 93190 | 169 | 18.1 |
|  | **55 to 64** | 48592 | 93 | 19.1 | 165336 | 313 | 18.9 |
|  | **65 to 74** | 141877 | 201 | 14.2 | 231077 | 345 | 14.9 |
|  | **>=75** | 174060 | 92 | 5.3 | 158598 | 141 | 8.9 |
| **Body Mass Index at primary** | **<18.5** | 2658 | 1 | 3.8 | 3682 | 3 | 8.1 |
|  | **[18.5-24.9]** | 51097 | 31 | 6.1 | 87542 | 95 | 10.9 |
|  | **[25-29.9]** | 92193 | 64 | 6.9 | 172498 | 154 | 8.9 |
|  | **>29.9** | 84542 | 65 | 7.7 | 177560 | 218 | 12.3 |
|  | **Unknown BMI** | 148407 | 242 | 16.3 | 206919 | 498 | 24.1 |
| **ASA grade** | **P1 - Fit and healthy** | 40275 | 71 | 17.6 | 104981 | 208 | 19.8 |
|  | **P2 - Mild disease not incapacitating** | 259297 | 276 | 10.6 | 446785 | 650 | 14.6 |
|  | **P3 - Incapacitating systemic disease** | 76495 | 54 | 7.1 | 93486 | 106 | 11.3 |
|  | **P4 - Life threatening disease** | 2783 | 2 | 7.2 | 2901 | 4 | 13.8 |
|  | **P5 - Expected to die within 24hrs with or without an operation** | 47 | 0 | 0.0 | 48 | 0 | 0.0 |
| **Fixation type** | **Cemented** | 345036 | 360 | 10.4 | 674 | 2 | 29.7 |
|  | **Hybrid** | 9 | 0 | 0.0 | 242042 | 225 | 9.3 |
|  | **Reverse hybrid** | 30394 | 36 | 11.8 | 89 | 1 | 112.4 |
|  | **Uncemented** | 35 | 0 | 0.0 | 395493 | 712 | 18.0 |
|  | **Unclassified** | 3423 | 7 | 20.5 | 9903 | 28 | 28.3 |
| **Stem composition** | **Stainless Steel** | 289364 | 259 | 8.9 | 178564 | 153 | 8.6 |
|  | **Cobalt chrome** | 54909 | 97 | 17.7 | 64626 | 76 | 11.8 |
|  | **Titanium** | 30680 | 39 | 12.7 | 391672 | 699 | 17.9 |
|  | **Other or unknown** | 3944 | 8 | 20.3 | 13339 | 40 | 30.0 |
| **Head size (mm)** | **22.25** | 12893 | 17 | 13.2 | 1021 | 7 | 68.6 |
|  | **26** | 18694 | 39 | 20.9 | 775 | 4 | 51.6 |
|  | **28** | 241452 | 294 | 12.2 | 144874 | 512 | 35.3 |
|  | **30-32** | 93203 | 43 | 4.6 | 268385 | 211 | 7.9 |
|  | **>=36** | 9232 | 3 | 3.3 | 223243 | 206 | 9.2 |
|  | **Unknown** | 3423 | 7 | 20.5 | 9903 | 28 | 28.3 |
| **Head Cup** | **Head: Alumina, Cup: HCLPE** | 6621 | 0 | 0.0 |  |  |  |
|  | **Head: Alumina, Cup: non-HCLPE** | 12255 | 12 | 9.8 |  |  |  |
|  | **Head: Cobalt Chrome, Cup: HCLPE** | 35069 | 12 | 3.4 |  |  |  |
|  | **Head: Cobalt Chrome,, Cup: non-HCLPE** | 69075 | 126 | 18.2 |  |  |  |
|  | **Head: Delta Ceramic, Cup: HCLPE** | 23279 | 7 | 3.0 |  |  |  |
|  | **Head: Delta Ceramic, Cup: non-HCLPE** | 14064 | 20 | 14.2 |  |  |  |
|  | **Head: Stainless Steel, Cup: HCLPE** | 35825 | 13 | 3.6 |  |  |  |
|  | **Head: Stainless Steel, Cup: non-HCLPE** | 182709 | 213 | 11.7 |  |  |  |
| **Shell composition** | **Cobalt Chrome** |  |  |  | 720 | 3 | 41.7 |
|  | **Stainless Steel** |  |  |  | 5273 | 8 | 15.2 |
|  | **Tantalum** |  |  |  | 5741 | 18 | 31.4 |
|  | **Titanium** |  |  |  | 629205 | 935 | 14.9 |
|  | **Unknown** |  |  |  | 7262 | 4 | 5.5 |
| **Head Liner** | **Head: Alumina, Liner: Alumina** |  |  |  | 27602 | 55 | 19.9 |
|  | **Head: Alumina, Liner: HCLPE** |  |  |  | 25191 | 18 | 7.2 |
|  | **Head: Alumina, Liner: non-HCLPE** |  |  |  | 3281 | 25 | 76.2 |
|  | **Head: Cobalt Chrome, Liner: HCLPE** |  |  |  | 183106 | 179 | 9.8 |
|  | **Head: Cobalt Chrome, Liner: non-HCLPE** |  |  |  | 42389 | 288 | 67.9 |
|  | **Head: Delta Ceramic, Liner: Delta Ceramic** |  |  |  | 119558 | 150 | 12.6 |
|  | **Head: Delta Ceramic, Liner: HCLPE** |  |  |  | 140539 | 86 | 6.1 |
|  | **Head: Delta Ceramic, Liner: non-HCLPE** |  |  |  | 13448 | 54 | 40.2 |
|  | **Head: Delta Ceramic, Pre-assembled implant** |  |  |  | 4614 | 3 | 6.5 |
|  | **Head: Other head materials, Pre-assembled implant** |  |  |  | 2648 | 1 | 3.8 |
|  | **Head: Oxidised, Liner: HCLPE** |  |  |  | 21263 | 12 | 5.6 |
|  | **Head: Stainless Steel, Liner: HCLPE** |  |  |  | 45672 | 17 | 3.7 |
|  | **Head: Stainless Steel, Liner: non-HCLPE** |  |  |  | 18890 | 80 | 42.3 |

ASA: American Society Anaesthesiologists Physical Status Classification

HCLPE: Highly Cross-Linked Polyethylene

**Table D:** Description of the studied sample and revision rate for malalignment

|  |  | **Monobloc** |  |  | **Modular** |  |  |
| --- | --- | --- | --- | --- | --- | --- | --- |
|  |  | **N** | **Revision(n)** | **Revision(per 10,000)** | **N** | **Revision(n)** | **Revision(per 10,000)** |
| **Year of primary surgery** | **<2010** | 111781 | 165 | 14.8 | 111028 | 335 | 30.2 |
|  | **2010-2014** | 127413 | 114 | 8.9 | 228155 | 393 | 17.2 |
|  | **2015-219** | 139703 | 64 | 4.6 | 309018 | 238 | 7.7 |
| **Gender** | **Female** | 251218 | 288 | 11.5 | 377914 | 773 | 20.5 |
|  | **Male** | 127679 | 144 | 11.3 | 270287 | 435 | 16.1 |
| **Age at primary (years)** | **<55** | 14368 | 19 | 13.2 | 93190 | 259 | 27.8 |
|  | **55 to 64** | 48592 | 70 | 14.4 | 165336 | 380 | 23.0 |
|  | **65 to 74** | 141877 | 192 | 13.5 | 231077 | 381 | 16.5 |
|  | **>=75** | 174060 | 151 | 8.7 | 158598 | 188 | 11.9 |
| **Body Mass Index at primary** | **<18.5** | 2658 | 1 | 3.8 | 3682 | 4 | 10.9 |
|  | **[18.5-24.9]** | 51097 | 41 | 8.0 | 87542 | 120 | 13.7 |
|  | **[25-29.9]** | 92193 | 89 | 9.6 | 172498 | 281 | 16.3 |
|  | **>29.9** | 84542 | 78 | 9.2 | 177560 | 291 | 16.4 |
|  | **Unknown BMI** | 148407 | 223 | 15.0 | 206919 | 512 | 24.7 |
| **ASA grade** | **P1 - Fit and healthy** | 40275 | 72 | 17.9 | 104981 | 246 | 23.4 |
|  | **P2 - Mild disease not incapacitating** | 259297 | 276 | 10.6 | 446785 | 821 | 18.4 |
|  | **P3 - Incapacitating systemic disease** | 76495 | 83 | 10.9 | 93486 | 136 | 14.6 |
|  | **P4 - Life threatening disease** | 2783 | 1 | 3.6 | 2901 | 5 | 17.2 |
|  | **P5 - Expected to die within 24hrs with or without an operation** | 47 | 0 | 0.0 | 48 | 0 | 0.0 |
| **Fixation type** | **Cemented** | 345036 | 372 | 10.8 | 674 | 2 | 29.7 |
|  | **Hybrid** | 9 | 0 | 0.0 | 242042 | 282 | 11.6 |
|  | **Reverse hybrid** | 30394 | 48 | 15.8 | 89 | 0 | 0.0 |
|  | **Uncemented** | 35 | 0 | 0.0 | 395493 | 898 | 22.7 |
|  | **Unclassified** | 3423 | 12 | 35.1 | 9903 | 26 | 26.3 |
| **Stem composition** | **Stainless Steel** | 289364 | 303 | 10.5 | 178564 | 197 | 11.0 |
|  | **Cobalt chrome** | 54909 | 65 | 11.8 | 64626 | 93 | 14.4 |
|  | **Titanium** | 30680 | 52 | 17.0 | 391672 | 878 | 22.4 |
|  | **Other or unknown** | 3944 | 12 | 30.4 | 13339 | 40 | 30.0 |
| **Head size (mm)** | **22.25** | 12893 | 14 | 10.9 | 1021 | 0 | 0.0 |
|  | **26** | 18694 | 38 | 20.3 | 775 | 1 | 12.9 |
|  | **28** | 241452 | 298 | 12.3 | 144874 | 419 | 28.9 |
|  | **30-32** | 93203 | 63 | 6.8 | 268385 | 423 | 15.8 |
|  | **>=36** | 9232 | 7 | 7.6 | 223243 | 339 | 15.2 |
|  | **Unknown** | 3423 | 12 | 35.1 | 9903 | 26 | 26.3 |
| **Head Cup** | **Head: Alumina, Cup: HCLPE** | 6621 | 3 | 4.5 |  |  |  |
|  | **Head: Alumina, Cup: non-HCLPE** | 12255 | 15 | 12.2 |  |  |  |
|  | **Head: Cobalt Chrome, Cup: HCLPE** | 35069 | 22 | 6.3 |  |  |  |
|  | **Head: Cobalt Chrome,, Cup: non-HCLPE** | 69075 | 108 | 15.6 |  |  |  |
|  | **Head: Delta Ceramic, Cup: HCLPE** | 23279 | 22 | 9.4 |  |  |  |
|  | **Head: Delta Ceramic, Cup: non-HCLPE** | 14064 | 16 | 11.4 |  |  |  |
|  | **Head: Stainless Steel, Cup: HCLPE** | 35825 | 17 | 4.8 |  |  |  |
|  | **Head: Stainless Steel, Cup: non-HCLPE** | 182709 | 229 | 12.5 |  |  |  |
| **Shell composition** | **Cobalt Chrome** |  |  |  | 720 | 2 | 27.8 |
|  | **Stainless Steel** |  |  |  | 5273 | 7 | 13.3 |
|  | **Tantalum** |  |  |  | 5741 | 13 | 22.6 |
|  | **Titanium** |  |  |  | 629205 | 1171 | 18.6 |
|  | **Unknown** |  |  |  | 7262 | 15 | 20.6 |
| **Head Liner** | **Head: Alumina, Liner: Alumina** |  |  |  | 27602 | 107 | 38.8 |
|  | **Head: Alumina, Liner: HCLPE** |  |  |  | 25191 | 17 | 6.8 |
|  | **Head: Alumina, Liner: non-HCLPE** |  |  |  | 3281 | 12 | 36.6 |
|  | **Head: Cobalt Chrome, Liner: HCLPE** |  |  |  | 183106 | 293 | 16.0 |
|  | **Head: Cobalt Chrome, Liner: non-HCLPE** |  |  |  | 42389 | 168 | 39.6 |
|  | **Head: Delta Ceramic, Liner: Delta Ceramic** |  |  |  | 119558 | 295 | 24.7 |
|  | **Head: Delta Ceramic, Liner: HCLPE** |  |  |  | 140539 | 137 | 9.8 |
|  | **Head: Delta Ceramic, Liner: non-HCLPE** |  |  |  | 13448 | 55 | 40.9 |
|  | **Head: Delta Ceramic, Pre-assembled implant** |  |  |  | 4614 | 9 | 19.5 |
|  | **Head: Other head materials, Pre-assembled implant** |  |  |  | 2648 | 6 | 22.7 |
|  | **Head: Oxidised, Liner: HCLPE** |  |  |  | 21263 | 27 | 12.7 |
|  | **Head: Stainless Steel, Liner: HCLPE** |  |  |  | 45672 | 35 | 7.7 |
|  | **Head: Stainless Steel, Liner: non-HCLPE** |  |  |  | 18890 | 47 | 24.9 |

ASA: American Society Anaesthesiologists Physical Status Classification

HCLPE: Highly Cross-Linked Polyethylene

**Table E:** Description of the studied sample and revision rate for dislocation or subluxation

|  |  | **Monobloc** |  |  | **Modular** |  |  |
| --- | --- | --- | --- | --- | --- | --- | --- |
|  |  | **N** | **Revision(n)** | **Revision(per 10,000)** | **N** | **Revision(n)** | **Revision(per 10,000)** |
| **Year of primary surgery** | **<2010** | 111781 | 573 | 51.3 | 111028 | 772 | 69.5 |
|  | **2010-2014** | 127413 | 527 | 41.4 | 228155 | 839 | 36.8 |
|  | **2015-219** | 139703 | 332 | 23.8 | 309018 | 776 | 25.1 |
| **Gender** | **Female** | 251218 | 1285 | 51.2 | 377914 | 1860 | 49.2 |
|  | **Male** | 127679 | 529 | 41.4 | 270287 | 1126 | 41.7 |
| **Age at primary (years)** | **<55** | 14368 | 63 | 43.8 | 93190 | 419 | 45.0 |
|  | **55 to 64** | 48592 | 238 | 49.0 | 165336 | 713 | 43.1 |
|  | **65 to 74** | 141877 | 703 | 49.5 | 231077 | 1088 | 47.1 |
|  | **>=75** | 174060 | 810 | 46.5 | 158598 | 766 | 48.3 |
| **Body Mass Index at primary** | **<18.5** | 2658 | 12 | 45.2 | 3682 | 8 | 21.7 |
|  | **[18.5-24.9]** | 51097 | 215 | 42.1 | 87542 | 309 | 35.3 |
|  | **[25-29.9]** | 92193 | 360 | 39.0 | 172498 | 660 | 38.3 |
|  | **>29.9** | 84542 | 359 | 42.5 | 177560 | 757 | 42.6 |
|  | **Unknown BMI** | 148407 | 868 | 58.5 | 206919 | 1252 | 60.5 |
| **ASA grade** | **P1 - Fit and healthy** | 40275 | 181 | 44.9 | 104981 | 414 | 39.4 |
|  | **P2 - Mild disease not incapacitating** | 259297 | 1212 | 46.7 | 446785 | 1999 | 44.7 |
|  | **P3 - Incapacitating systemic disease** | 76495 | 406 | 53.1 | 93486 | 553 | 59.2 |
|  | **P4 - Life threatening disease** | 2783 | 15 | 53.9 | 2901 | 20 | 68.9 |
|  | **P5 - Expected to die within 24hrs with or without an operation** | 47 | 0 | 0.0 | 48 | 0 | 0.0 |
| **Fixation type** | **Cemented** | 345036 | 1642 | 47.6 | 674 | 4 | 59.3 |
|  | **Hybrid** | 9 | 0 | 0.0 | 242042 | 1069 | 44.2 |
|  | **Reverse hybrid** | 30394 | 142 | 46.7 | 89 | 0 | 0.0 |
|  | **Uncemented** | 35 | 2 | 571.4 | 395493 | 1852 | 46.8 |
|  | **Unclassified** | 3423 | 28 | 81.8 | 9903 | 61 | 61.6 |
| **Stem composition** | **Stainless Steel** | 289364 | 1349 | 46.6 | 178564 | 722 | 40.4 |
|  | **Cobalt chrome** | 54909 | 289 | 52.6 | 64626 | 365 | 56.5 |
|  | **Titanium** | 30680 | 143 | 46.6 | 391672 | 1808 | 46.2 |
|  | **Other or unknown** | 3944 | 33 | 83.7 | 13339 | 91 | 68.2 |
| **Head size (mm)** | **22.25** | 12893 | 145 | 112.5 | 1021 | 11 | 107.7 |
|  | **26** | 18694 | 117 | 62.6 | 775 | 12 | 154.8 |
|  | **28** | 241452 | 1228 | 50.9 | 144874 | 1282 | 88.5 |
|  | **30-32** | 93203 | 271 | 29.1 | 268385 | 968 | 36.1 |
|  | **>=36** | 9232 | 25 | 27.1 | 223243 | 652 | 29.2 |
|  | **Unknown** | 3423 | 28 | 81.8 | 9903 | 61 | 61.6 |
| **Head Cup** | **Head: Alumina, Cup: HCLPE** | 6621 | 17 | 25.7 |  |  |  |
|  | **Head: Alumina, Cup: non-HCLPE** | 12255 | 46 | 37.5 |  |  |  |
|  | **Head: Cobalt Chrome, Cup: HCLPE** | 35069 | 108 | 30.8 |  |  |  |
|  | **Head: Cobalt Chrome,, Cup: non-HCLPE** | 69075 | 411 | 59.5 |  |  |  |
|  | **Head: Delta Ceramic, Cup: HCLPE** | 23279 | 54 | 23.2 |  |  |  |
|  | **Head: Delta Ceramic, Cup: non-HCLPE** | 14064 | 78 | 55.5 |  |  |  |
|  | **Head: Stainless Steel, Cup: HCLPE** | 35825 | 102 | 28.5 |  |  |  |
|  | **Head: Stainless Steel, Cup: non-HCLPE** | 182709 | 998 | 54.6 |  |  |  |
| **Shell composition** | **Cobalt Chrome** |  |  |  | 720 | 5 | 69.4 |
|  | **Stainless Steel** |  |  |  | 5273 | 12 | 22.8 |
|  | **Tantalum** |  |  |  | 5741 | 67 | 116.7 |
|  | **Titanium** |  |  |  | 629205 | 2868 | 45.6 |
|  | **Unknown** |  |  |  | 7262 | 34 | 46.8 |
| **Head Liner** | **Head: Alumina, Liner: Alumina** |  |  |  | 27602 | 108 | 39.1 |
|  | **Head: Alumina, Liner: HCLPE** |  |  |  | 25191 | 88 | 34.9 |
|  | **Head: Alumina, Liner: non-HCLPE** |  |  |  | 3281 | 30 | 91.4 |
|  | **Head: Cobalt Chrome, Liner: HCLPE** |  |  |  | 183106 | 840 | 45.9 |
|  | **Head: Cobalt Chrome, Liner: non-HCLPE** |  |  |  | 42389 | 432 | 101.9 |
|  | **Head: Delta Ceramic, Liner: Delta Ceramic** |  |  |  | 119558 | 427 | 35.7 |
|  | **Head: Delta Ceramic, Liner: HCLPE** |  |  |  | 140539 | 456 | 32.5 |
|  | **Head: Delta Ceramic, Liner: non-HCLPE** |  |  |  | 13448 | 123 | 91.5 |
|  | **Head: Delta Ceramic, Pre-assembled implant** |  |  |  | 4614 | 8 | 17.3 |
|  | **Head: Other head materials, Pre-assembled implant** |  |  |  | 2648 | 26 | 98.2 |
|  | **Head: Oxidised, Liner: HCLPE** |  |  |  | 21263 | 73 | 34.3 |
|  | **Head: Stainless Steel, Liner: HCLPE** |  |  |  | 45672 | 182 | 39.8 |
|  | **Head: Stainless Steel, Liner: non-HCLPE** |  |  |  | 18890 | 193 | 102.2 |

ASA: American Society Anaesthesiologists Physical Status Classification

HCLPE: Highly Cross-Linked Polyethylene

**Table F:** Description of the studied sample and revision rate for pain

|  |  | **Monobloc** |  |  | **Modular** |  |  |
| --- | --- | --- | --- | --- | --- | --- | --- |
|  |  | **N** | **Revision(n)** | **Revision(per 10,000)** | **N** | **Revision(n)** | **Revision(per 10,000)** |
| **Year of primary surgery** | **<2010** | 111781 | 281 | 25.1 | 111028 | 490 | 44.1 |
|  | **2010-2014** | 127413 | 139 | 10.9 | 228155 | 488 | 21.4 |
|  | **2015-219** | 139703 | 35 | 2.5 | 309018 | 97 | 3.1 |
| **Gender** | **Female** | 251218 | 379 | 15.1 | 377914 | 742 | 19.6 |
|  | **Male** | 127679 | 194 | 15.2 | 270287 | 601 | 22.2 |
| **Age at primary (years)** | **<55** | 14368 | 57 | 39.7 | 93190 | 365 | 39.2 |
|  | **55 to 64** | 48592 | 124 | 25.5 | 165336 | 431 | 26.1 |
|  | **65 to 74** | 141877 | 253 | 17.8 | 231077 | 379 | 16.4 |
|  | **>=75** | 174060 | 139 | 8.0 | 158598 | 168 | 10.6 |
| **Body Mass Index at primary** | **<18.5** | 2658 | 4 | 15.1 | 3682 | 4 | 10.9 |
|  | **[18.5-24.9]** | 51097 | 45 | 8.8 | 87542 | 124 | 14.2 |
|  | **[25-29.9]** | 92193 | 96 | 10.4 | 172498 | 287 | 16.6 |
|  | **>29.9** | 84542 | 85 | 10.1 | 177560 | 334 | 18.8 |
|  | **Unknown BMI** | 148407 | 343 | 23.1 | 206919 | 594 | 28.7 |
| **ASA grade** | **P1 - Fit and healthy** | 40275 | 100 | 24.8 | 104981 | 293 | 27.9 |
|  | **P2 - Mild disease not incapacitating** | 259297 | 385 | 14.9 | 446785 | 919 | 20.6 |
|  | **P3 - Incapacitating systemic disease** | 76495 | 87 | 11.4 | 93486 | 129 | 13.8 |
|  | **P4 - Life threatening disease** | 2783 | 1 | 3.6 | 2901 | 2 | 6.9 |
|  | **P5 - Expected to die within 24hrs with or without an operation** | 47 | 0 | 0.0 | 48 | 0 | 0.0 |
| **Fixation type** | **Cemented** | 345036 | 510 | 14.8 | 674 | 1 | 14.8 |
|  | **Hybrid** | 9 | 0 | 0.0 | 242042 | 292 | 12.1 |
|  | **Reverse hybrid** | 30394 | 53 | 17.4 | 89 | 0 | 0.0 |
|  | **Uncemented** | 35 | 1 | 285.7 | 395493 | 1021 | 25.8 |
|  | **Unclassified** | 3423 | 9 | 26.3 | 9903 | 29 | 29.3 |
| **Stem composition** | **Stainless Steel** | 289364 | 396 | 13.7 | 178564 | 218 | 12.2 |
|  | **Cobalt chrome** | 54909 | 111 | 20.2 | 64626 | 87 | 13.5 |
|  | **Titanium** | 30680 | 55 | 17.9 | 391672 | 997 | 25.5 |
|  | **Other or unknown** | 3944 | 11 | 27.9 | 13339 | 41 | 30.7 |
| **Head size (mm)** | **22.25** | 12893 | 28 | 21.7 | 1021 | 5 | 49.0 |
|  | **26** | 18694 | 59 | 31.6 | 775 | 0 | 0.0 |
|  | **28** | 241452 | 398 | 16.5 | 144874 | 436 | 30.1 |
|  | **30-32** | 93203 | 68 | 7.3 | 268385 | 392 | 14.6 |
|  | **>=36** | 9232 | 11 | 11.9 | 223243 | 481 | 21.5 |
|  | **Unknown** | 3423 | 9 | 26.3 | 9903 | 29 | 29.3 |
| **Head Cup** | **Head: Alumina, Cup: HCLPE** | 6621 | 8 | 12.1 |  |  |  |
|  | **Head: Alumina, Cup: non-HCLPE** | 12255 | 28 | 22.9 |  |  |  |
|  | **Head: Cobalt Chrome, Cup: HCLPE** | 35069 | 20 | 5.7 |  |  |  |
|  | **Head: Cobalt Chrome,, Cup: non-HCLPE** | 69075 | 135 | 19.5 |  |  |  |
|  | **Head: Delta Ceramic, Cup: HCLPE** | 23279 | 18 | 7.7 |  |  |  |
|  | **Head: Delta Ceramic, Cup: non-HCLPE** | 14064 | 30 | 21.3 |  |  |  |
|  | **Head: Stainless Steel, Cup: HCLPE** | 35825 | 19 | 5.3 |  |  |  |
|  | **Head: Stainless Steel, Cup: non-HCLPE** | 182709 | 315 | 17.2 |  |  |  |
| **Shell composition** | **Cobalt Chrome** |  |  |  | 720 | 6 | 83.3 |
|  | **Stainless Steel** |  |  |  | 5273 | 9 | 17.1 |
|  | **Tantalum** |  |  |  | 5741 | 11 | 19.2 |
|  | **Titanium** |  |  |  | 629205 | 1297 | 20.6 |
|  | **Unknown** |  |  |  | 7262 | 20 | 27.5 |
| **Head Liner** | **Head: Alumina, Liner: Alumina** |  |  |  | 27602 | 161 | 58.3 |
|  | **Head: Alumina, Liner: HCLPE** |  |  |  | 25191 | 45 | 17.9 |
|  | **Head: Alumina, Liner: non-HCLPE** |  |  |  | 3281 | 15 | 45.7 |
|  | **Head: Cobalt Chrome, Liner: HCLPE** |  |  |  | 183106 | 249 | 13.6 |
|  | **Head: Cobalt Chrome, Liner: non-HCLPE** |  |  |  | 42389 | 213 | 50.3 |
|  | **Head: Delta Ceramic, Liner: Delta Ceramic** |  |  |  | 119558 | 395 | 33.0 |
|  | **Head: Delta Ceramic, Liner: HCLPE** |  |  |  | 140539 | 90 | 6.4 |
|  | **Head: Delta Ceramic, Liner: non-HCLPE** |  |  |  | 13448 | 45 | 33.5 |
|  | **Head: Delta Ceramic, Pre-assembled implant** |  |  |  | 4614 | 16 | 34.7 |
|  | **Head: Other head materials, Pre-assembled implant** |  |  |  | 2648 | 4 | 15.1 |
|  | **Head: Oxidised, Liner: HCLPE** |  |  |  | 21263 | 17 | 8.0 |
|  | **Head: Stainless Steel, Liner: HCLPE** |  |  |  | 45672 | 26 | 5.7 |
|  | **Head: Stainless Steel, Liner: non-HCLPE** |  |  |  | 18890 | 67 | 35.5 |

ASA: American Society Anaesthesiologists Physical Status Classification

HCLPE: Highly Cross-Linked Polyethylene

**Table G:** Description of the studied sample and revision rate for infection

|  |  | **Monobloc** |  |  | **Modular** |  |  |
| --- | --- | --- | --- | --- | --- | --- | --- |
|  |  | **N** | **Revision(n)** | **Revision(per 10,000)** | **N** | **Revision(n)** | **Revision(per 10,000)** |
| **Year of primary surgery** | **<2010** | 111781 | 460 | 41.2 | 111028 | 424 | 38.2 |
|  | **2010-2014** | 127413 | 393 | 30.8 | 228155 | 706 | 30.9 |
|  | **2015-219** | 139703 | 360 | 25.8 | 309018 | 732 | 23.7 |
| **Gender** | **Female** | 251218 | 811 | 32.3 | 377914 | 1018 | 26.9 |
|  | **Male** | 127679 | 724 | 56.7 | 270287 | 1308 | 48.4 |
| **Age at primary (years)** | **<55** | 14368 | 104 | 72.4 | 93190 | 394 | 42.3 |
|  | **55 to 64** | 48592 | 251 | 51.7 | 165336 | 682 | 41.3 |
|  | **65 to 74** | 141877 | 627 | 44.2 | 231077 | 776 | 33.6 |
|  | **>=75** | 174060 | 553 | 31.8 | 158598 | 474 | 29.9 |
| **Body Mass Index at primary** | **<18.5** | 2658 | 8 | 30.1 | 3682 | 13 | 35.3 |
|  | **[18.5-24.9]** | 51097 | 124 | 24.3 | 87542 | 175 | 20.0 |
|  | **[25-29.9]** | 92193 | 254 | 27.5 | 172498 | 476 | 27.6 |
|  | **>29.9** | 84542 | 453 | 53.6 | 177560 | 873 | 49.2 |
|  | **Unknown BMI** | 148407 | 696 | 46.9 | 206919 | 789 | 38.1 |
| **ASA grade** | **P1 - Fit and healthy** | 40275 | 145 | 36.0 | 104981 | 313 | 29.8 |
|  | **P2 - Mild disease not incapacitating** | 259297 | 1039 | 40.1 | 446785 | 1528 | 34.2 |
|  | **P3 - Incapacitating systemic disease** | 76495 | 336 | 43.9 | 93486 | 470 | 50.3 |
|  | **P4 - Life threatening disease** | 2783 | 15 | 53.9 | 2901 | 15 | 51.7 |
|  | **P5 - Expected to die within 24hrs with or without an operation** | 47 | 0 | 0.0 | 48 | 0 | 0.0 |
| **Fixation type** | **Cemented** | 345036 | 1397 | 40.5 | 674 | 2 | 29.7 |
|  | **Hybrid** | 9 | 0 | 0.0 | 242042 | 925 | 38.2 |
|  | **Reverse hybrid** | 30394 | 117 | 38.5 | 89 | 1 | 112.4 |
|  | **Uncemented** | 35 | 1 | 285.7 | 395493 | 1351 | 34.2 |
|  | **Unclassified** | 3423 | 20 | 58.4 | 9903 | 47 | 47.5 |
| **Stem composition** | **Stainless Steel** | 289364 | 1145 | 39.6 | 178564 | 693 | 38.8 |
|  | **Cobalt chrome** | 54909 | 249 | 45.3 | 64626 | 242 | 37.5 |
|  | **Titanium** | 30680 | 115 | 37.5 | 391672 | 1318 | 33.7 |
|  | **Other or unknown** | 3944 | 26 | 65.9 | 13339 | 73 | 54.7 |
| **Head size (mm)** | **22.25** | 12893 | 65 | 50.4 | 1021 | 10 | 97.9 |
|  | **26** | 18694 | 84 | 44.9 | 775 | 4 | 51.6 |
|  | **28** | 241452 | 989 | 41.0 | 144874 | 546 | 37.7 |
|  | **30-32** | 93203 | 335 | 35.9 | 268385 | 828 | 30.9 |
|  | **>=36** | 9232 | 42 | 45.5 | 223243 | 891 | 39.9 |
|  | **Unknown** | 3423 | 20 | 58.4 | 9903 | 47 | 47.5 |
| **Head Cup** | **Head: Alumina, Cup: HCLPE** | 6621 | 23 | 34.7 |  |  |  |
|  | **Head: Alumina, Cup: non-HCLPE** | 12255 | 60 | 49.0 |  |  |  |
|  | **Head: Cobalt Chrome, Cup: HCLPE** | 35069 | 112 | 31.9 |  |  |  |
|  | **Head: Cobalt Chrome,, Cup: non-HCLPE** | 69075 | 296 | 42.8 |  |  |  |
|  | **Head: Delta Ceramic, Cup: HCLPE** | 23279 | 76 | 32.7 |  |  |  |
|  | **Head: Delta Ceramic, Cup: non-HCLPE** | 14064 | 59 | 42.0 |  |  |  |
|  | **Head: Stainless Steel, Cup: HCLPE** | 35825 | 94 | 26.2 |  |  |  |
|  | **Head: Stainless Steel, Cup: non-HCLPE** | 182709 | 815 | 44.6 |  |  |  |
| **Shell composition** | **Cobalt Chrome** |  |  |  | 720 | 3 | 41.7 |
|  | **Stainless Steel** |  |  |  | 5273 | 27 | 51.2 |
|  | **Tantalum** |  |  |  | 5741 | 31 | 54.0 |
|  | **Titanium** |  |  |  | 629205 | 2251 | 35.8 |
|  | **Unknown** |  |  |  | 7262 | 14 | 19.3 |
| **Head Liner** | **Head: Alumina, Liner: Alumina** |  |  |  | 27602 | 112 | 40.6 |
|  | **Head: Alumina, Liner: HCLPE** |  |  |  | 25191 | 97 | 38.5 |
|  | **Head: Alumina, Liner: non-HCLPE** |  |  |  | 3281 | 18 | 54.9 |
|  | **Head: Cobalt Chrome, Liner: HCLPE** |  |  |  | 183106 | 660 | 36.0 |
|  | **Head: Cobalt Chrome, Liner: non-HCLPE** |  |  |  | 42389 | 196 | 46.2 |
|  | **Head: Delta Ceramic, Liner: Delta Ceramic** |  |  |  | 119558 | 437 | 36.5 |
|  | **Head: Delta Ceramic, Liner: HCLPE** |  |  |  | 140539 | 404 | 28.8 |
|  | **Head: Delta Ceramic, Liner: non-HCLPE** |  |  |  | 13448 | 43 | 32.0 |
|  | **Head: Delta Ceramic, Pre-assembled implant** |  |  |  | 4614 | 8 | 17.3 |
|  | **Head: Other head materials, Pre-assembled implant** |  |  |  | 2648 | 6 | 22.7 |
|  | **Head: Oxidised, Liner: HCLPE** |  |  |  | 21263 | 52 | 24.5 |
|  | **Head: Stainless Steel, Liner: HCLPE** |  |  |  | 45672 | 169 | 37.0 |
|  | **Head: Stainless Steel, Liner: non-HCLPE** |  |  |  | 18890 | 124 | 65.6 |

ASA: American Society Anaesthesiologists Physical Status Classification

HCLPE: Highly Cross-Linked Polyethylene

**Table H:** Description of the studied sample and revision rate for any other reason(s)

|  |  | **Monobloc** |  |  | **Modular** |  |  |
| --- | --- | --- | --- | --- | --- | --- | --- |
|  |  | **N** | **Revision(n)** | **Revision(per 10,000)** | **N** | **Revision(n)** | **Revision(per 10,000)** |
| **Year of primary surgery** | **<2010** | 111781 | 474 | 42.5 | 111028 | 829 | 74.7 |
|  | **2010-2014** | 127413 | 241 | 18.9 | 228155 | 768 | 33.7 |
|  | **2015-219** | 139703 | 94 | 6.7 | 309018 | 345 | 11.2 |
| **Gender** | **Female** | 251218 | 580 | 23.1 | 377914 | 1343 | 35.5 |
|  | **Male** | 127679 | 439 | 34.4 | 270287 | 1096 | 40.5 |
| **Age at primary (years)** | **<55** | 14368 | 64 | 44.5 | 93190 | 540 | 58.0 |
|  | **55 to 64** | 48592 | 221 | 45.5 | 165336 | 829 | 50.1 |
|  | **65 to 74** | 141877 | 455 | 32.1 | 231077 | 749 | 32.4 |
|  | **>=75** | 174060 | 279 | 16.0 | 158598 | 321 | 20.2 |
| **Body Mass Index at primary** | **<18.5** | 2658 | 5 | 18.8 | 3682 | 10 | 27.2 |
|  | **[18.5-24.9]** | 51097 | 98 | 19.2 | 87542 | 236 | 27.0 |
|  | **[25-29.9]** | 92193 | 194 | 21.0 | 172498 | 476 | 27.6 |
|  | **>29.9** | 84542 | 178 | 21.0 | 177560 | 604 | 34.0 |
|  | **Unknown BMI** | 148407 | 544 | 36.7 | 206919 | 1113 | 53.8 |
| **ASA grade** | **P1 - Fit and healthy** | 40275 | 158 | 39.2 | 104981 | 544 | 51.8 |
|  | **P2 - Mild disease not incapacitating** | 259297 | 703 | 27.1 | 446785 | 1599 | 35.8 |
|  | **P3 - Incapacitating systemic disease** | 76495 | 153 | 20.0 | 93486 | 289 | 30.9 |
|  | **P4 - Life threatening disease** | 2783 | 5 | 18.0 | 2901 | 7 | 24.1 |
|  | **P5 - Expected to die within 24hrs with or without an operation** | 47 | 0 | 0.0 | 48 | 0 | 0.0 |
| **Fixation type** | **Cemented** | 345036 | 908 | 26.3 | 674 | 5 | 74.2 |
|  | **Hybrid** | 9 | 0 | 0.0 | 242042 | 623 | 25.7 |
|  | **Reverse hybrid** | 30394 | 91 | 29.9 | 89 | 2 | 224.7 |
|  | **Uncemented** | 35 | 0 | 0.0 | 395493 | 1759 | 44.5 |
|  | **Unclassified** | 3423 | 20 | 58.4 | 9903 | 50 | 50.5 |
| **Stem composition** | **Stainless Steel** | 289364 | 689 | 23.8 | 178564 | 467 | 26.1 |
|  | **Cobalt chrome** | 54909 | 210 | 38.3 | 64626 | 167 | 25.8 |
|  | **Titanium** | 30680 | 93 | 30.3 | 391672 | 1729 | 44.1 |
|  | **Other or unknown** | 3944 | 27 | 68.5 | 13339 | 76 | 57.0 |
| **Head size (mm)** | **22.25** | 12893 | 37 | 28.7 | 1021 | 2 | 19.6 |
|  | **26** | 18694 | 81 | 43.3 | 775 | 3 | 38.7 |
|  | **28** | 241452 | 693 | 28.7 | 144874 | 848 | 58.5 |
|  | **30-32** | 93203 | 162 | 17.4 | 268385 | 648 | 24.1 |
|  | **>=36** | 9232 | 26 | 28.2 | 223243 | 888 | 39.8 |
|  | **Unknown** | 3423 | 20 | 58.4 | 9903 | 50 | 50.5 |
| **Head Cup** | **Head: Alumina, Cup: HCLPE** | 6621 | 17 | 25.7 |  |  |  |
|  | **Head: Alumina, Cup: non-HCLPE** | 12255 | 40 | 32.6 |  |  |  |
|  | **Head: Cobalt Chrome, Cup: HCLPE** | 35069 | 56 | 16.0 |  |  |  |
|  | **Head: Cobalt Chrome,, Cup: non-HCLPE** | 69075 | 269 | 38.9 |  |  |  |
|  | **Head: Delta Ceramic, Cup: HCLPE** | 23279 | 33 | 14.2 |  |  |  |
|  | **Head: Delta Ceramic, Cup: non-HCLPE** | 14064 | 35 | 24.9 |  |  |  |
|  | **Head: Stainless Steel, Cup: HCLPE** | 35825 | 32 | 8.9 |  |  |  |
|  | **Head: Stainless Steel, Cup: non-HCLPE** | 182709 | 537 | 29.4 |  |  |  |
| **Shell composition** | **Cobalt Chrome** |  |  |  | 720 | 3 | 41.7 |
|  | **Stainless Steel** |  |  |  | 5273 | 17 | 32.2 |
|  | **Tantalum** |  |  |  | 5741 | 23 | 40.1 |
|  | **Titanium** |  |  |  | 629205 | 2372 | 37.7 |
|  | **Unknown** |  |  |  | 7262 | 24 | 33.1 |
| **Head Liner** | **Head: Alumina, Liner: Alumina** |  |  |  | 27602 | 240 | 86.9 |
|  | **Head: Alumina, Liner: HCLPE** |  |  |  | 25191 | 64 | 25.4 |
|  | **Head: Alumina, Liner: non-HCLPE** |  |  |  | 3281 | 30 | 91.4 |
|  | **Head: Cobalt Chrome, Liner: HCLPE** |  |  |  | 183106 | 478 | 26.1 |
|  | **Head: Cobalt Chrome, Liner: non-HCLPE** |  |  |  | 42389 | 358 | 84.5 |
|  | **Head: Delta Ceramic, Liner: Delta Ceramic** |  |  |  | 119558 | 706 | 59.0 |
|  | **Head: Delta Ceramic, Liner: HCLPE** |  |  |  | 140539 | 214 | 15.2 |
|  | **Head: Delta Ceramic, Liner: non-HCLPE** |  |  |  | 13448 | 85 | 63.2 |
|  | **Head: Delta Ceramic, Pre-assembled implant** |  |  |  | 4614 | 17 | 36.8 |
|  | **Head: Other head materials, Pre-assembled implant** |  |  |  | 2648 | 7 | 26.4 |
|  | **Head: Oxidised, Liner: HCLPE** |  |  |  | 21263 | 36 | 16.9 |
|  | **Head: Stainless Steel, Liner: HCLPE** |  |  |  | 45672 | 70 | 15.3 |
|  | **Head: Stainless Steel, Liner: non-HCLPE** |  |  |  | 18890 | 134 | 70.9 |

ASA: American Society Anaesthesiologists Physical Status Classification

HCLPE: Highly Cross-Linked Polyethylene

## **Supplementary 2:** Hazard Ratios (HR) and 95% Confidence Intervals (CI) of revision procedures

**Table I:** Monobloc acetabular component- All-cause revision Hazard Ratio (HR) and 95% Confidence Interval (CI) by time-point from primary procedure-Reference: Implant with cobalt chrome head and highly cross-linked polyethylene cup

|  | **6 months** | |  | **1 year** | |  | **2 years** | |  | **5 years** | |  | **10 years** | |  | **15 years** | |  |
| --- | --- | --- | --- | --- | --- | --- | --- | --- | --- | --- | --- | --- | --- | --- | --- | --- | --- | --- |
|  | **HR** | **95% CI** | **P-value** | **HR** | **95% CI** | **P-value** | **HR** | **95% CI** | **P-value** | **HR** | **95% CI** | **P-value** | **HR** | **95% CI** | **P-value** | **HR** | **95% CI** | **P-value** |
| **Head: Stainless Steel, Cup: HCLPE** | 0.81 | [0.70, 0.95] | 0.010 | 0.81 | [0.70, 0.95] | 0.010 | 0.81 | [0.69, 0.95] | 0.014 | 0.8 | [0.66, 0.97] | 0.030 | 0.8 | [0.66, 0.97] | 0.030 | 0.8 | [0.65, 0.98] | 0.041 |
| **Head: Delta Ceramic, Cup: HCLPE** | 0.66 | [0.55, 0.78] | <0.001 | 0.67 | [0.57, 0.79] | <0.001 | 0.65 | [0.54, 0.77] | <0.001 | 0.61 | [0.50, 0.75] | <0.001 | 0.61 | [0.50, 0.75] | <0.001 | 0.6 | [0.48, 0.74] | <0.001 |
| **Head: Alumina, Cup: HCLPE** | 0.62 | [0.48, 0.80] | <0.001 | 0.63 | [0.49, 0.81] | <0.001 | 0.67 | [0.52, 0.85] | 0.002 | 0.73 | [0.55, 0.99] | 0.044 | 0.75 | [0.55, 1.03] | 0.079 | 0.78 | [0.55, 1.09] | 0.145 |
| **Head: Stainless Steel, Cup: non-HCLPE** | 1.2 | [1.07, 1.36] | 0.005 | 1.2 | [1.07, 1.36] | 0.005 | 1.19 | [1.05, 1.36] | 0.012 | 1.18 | [1.02, 1.36] | 0.031 | 1.18 | [1.02, 1.36] | 0.031 | 1.17 | [1.00, 1.37] | 0.059 |
| **Head: Cobalt Chrome, Cup: non-HCLPE** | 1.32 | [1.17, 1.49] | <0.001 | 1.32 | [1.17, 1.49] | <0.001 | 1.31 | [1.15, 1.48] | <0.001 | 1.28 | [1.11, 1.48] | 0.001 | 1.28 | [1.10, 1.48] | 0.002 | 1.27 | [1.09, 1.49] | 0.004 |
| **Head: Delta Ceramic, Cup: non-HCLPE** | 0.88 | [0.75, 1.03] | 0.115 | 0.88 | [0.75, 1.03] | 0.115 | 0.87 | [0.74, 1.03] | 0.102 | 0.86 | [0.71, 1.04] | 0.120 | 0.86 | [0.71, 1.05] | 0.127 | 0.86 | [0.70, 1.05] | 0.138 |
| **Head: Alumina, Cup: non-HCLPE** | 0.84 | [0.70, 1.00] | 0.064 | 0.84 | [0.71, 1.00] | 0.054 | 0.87 | [0.73, 1.03] | 0.113 | 0.91 | [0.75, 1.11] | 0.256 | 0.92 | [0.76, 1.13] | 0.284 | 0.94 | [0.76, 1.16] | 0.338 |

Flexible parametric survival model adjusted for year of primary surgery, patient gender, age, Body Mass Index,  American Society of Anesthesiologists grade, implant fixation, stem composition and head size. HCLPE: Highly CrossLinked PolyethylenE.

Wald test

**Table J:** Monobloc acetabular component- Revision for aseptic loosening Hazard Ratio (HR) and 95% Confidence Interval (CI) by time-point from primary procedure-Reference: Implant with cobalt chrome head and highly cross-linked polyethylene cup

|  | **6 months** | |  | **1 year** | |  | **2 years** | |  | **5 years** | |  | **10 years** | |  | **15 years** | |  |
| --- | --- | --- | --- | --- | --- | --- | --- | --- | --- | --- | --- | --- | --- | --- | --- | --- | --- | --- |
|  | **HR** | **95% CI** | **P-value** | **HR** | **95% CI** | **P-value** | **HR** | **95% CI** | **P-value** | **HR** | **95% CI** | **P-value** | **HR** | **95% CI** | **P-value** | **HR** | **95% CI** | **P-value** |
| **Head: Stainless Steel, Cup: HCLPE** | 0.60 | [0.34, 1.06] | 0.085 | 0.69 | [0.45, 1.06] | 0.094 | 0.79 | [0.55, 1.13] | 0.175 | 0.94 | [0.62, 1.41] | 0.382 | 1.02 | [0.63, 1.67] | 0.398 | 1.11 | [0.63, 1.95] | 0.374 |
| **Head: Delta Ceramic, Cup: HCLPE** | 0.59 | [0.36, 0.97] | 0.045 | 0.63 | [0.42, 0.94] | 0.032 | 0.67 | [0.47, 0.96] | 0.036 | 0.74 | [0.48, 1.12] | 0.151 | 0.77 | [0.47, 1.25] | 0.231 | 0.8 | [0.46, 1.40] | 0.293 |
| **Head: Alumina, Cup: HCLPE** | 0.63 | [0.29, 1.38] | 0.203 | 0.65 | [0.35, 1.19] | 0.154 | 0.66 | [0.39, 1.13] | 0.124 | 0.69 | [0.37, 1.27] | 0.199 | 0.7 | [0.34, 1.43] | 0.248 | 0.71 | [0.31, 1.62] | 0.287 |
| **Head: Stainless Steel, Cup: non-HCLPE** | 1.52 | [1.08, 2.14] | 0.022 | 1.67 | [1.25, 2.23] | <0.001 | 1.82 | [1.39, 2.40] | <0.001 | 2.06 | [1.50, 2.81] | <0.001 | 2.18 | [1.54, 3.10] | <0.001 | 2.3 | [1.55, 3.41] | <0.001 |
| **Head: Cobalt Chrome, Cup: non-HCLPE** | 1.56 | [1.11, 2.20] | 0.016 | 1.6 | [1.20, 2.13] | 0.002 | 1.64 | [1.26, 2.14] | <0.001 | 1.69 | [1.24, 2.30] | 0.002 | 1.72 | [1.21, 2.42] | 0.004 | 1.74 | [1.18, 2.57] | 0.008 |
| **Head: Delta Ceramic, Cup: non-HCLPE** | 0.83 | [0.48, 1.43] | 0.319 | 0.93 | [0.61, 1.42] | 0.377 | 1.04 | [0.74, 1.47] | 0.389 | 1.2 | [0.84, 1.71] | 0.241 | 1.29 | [0.86, 1.93] | 0.186 | 1.38 | [0.86, 2.19] | 0.160 |
| **Head: Alumina, Cup: non-HCLPE** | 0.87 | [0.48, 1.57] | 0.359 | 0.98 | [0.62, 1.55] | 0.397 | 1.1 | [0.76, 1.59] | 0.351 | 1.29 | [0.90, 1.84] | 0.151 | 1.39 | [0.92, 2.09] | 0.116 | 1.49 | [0.93, 2.39] | 0.101 |

Flexible parametric survival model adjusted for year of primary surgery, patient gender, age, Body Mass Index,  American Society of Anesthesiologists grade, implant fixation, stem composition and head size. HCLPE: Highly CrossLinked PolyethylenE.
Wald test

**Table K:** Monobloc acetabular component- Revision for peri-prosthetic fracture Hazard Ratio (HR) and 95% Confidence Interval (CI) by time-point from primary procedure-Reference: Implant with cobalt chrome head and highly cross-linked polyethylene cup

|  | **6 months** | |  | **1 year** | |  | **2 years** | |  | **5 years** | |  | **10 years** | |  | **15 years** | |  |
| --- | --- | --- | --- | --- | --- | --- | --- | --- | --- | --- | --- | --- | --- | --- | --- | --- | --- | --- |
|  | **HR** | **95% CI** | **P-value** | **HR** | **95% CI** | **P-value** | **HR** | **95% CI** | **P-value** | **HR** | **95% CI** | **P-value** | **HR** | **95% CI** | **P-value** | **HR** | **95% CI** | **P-value** |
| **Head: Stainless Steel, Cup: HCLPE** | 0.66 | [0.46, 0.94] | 0.030 | 0.62 | [0.41, 0.94] | 0.031 | 0.61 | [0.40, 0.95] | 0.032 | 0.62 | [0.41, 0.94] | 0.031 | 0.61 | [0.38, 0.97] | 0.047 | 0.6 | [0.36, 1.00] | 0.058 |
| **Head: Delta Ceramic, Cup: HCLPE** | 0.43 | [0.22, 0.84] | 0.019 | 0.47 | [0.26, 0.85] | 0.018 | 0.49 | [0.26, 0.90] | 0.032 | 0.49 | [0.26, 0.92] | 0.034 | 0.51 | [0.24, 1.07] | 0.084 | 0.52 | [0.22, 1.22] | 0.130 |
| **Head: Alumina, Cup: HCLPE** | 0.54 | [0.42, 0.70] | <0.001 | 0.61 | [0.46, 0.80] | <0.001 | 0.63 | [0.47, 0.84] | 0.003 | 0.63 | [0.48, 0.84] | 0.002 | 0.67 | [0.49, 0.92] | 0.018 | 0.69 | [0.49, 0.98] | 0.044 |
| **Head: Stainless Steel, Cup: non-HCLPE** | 1.21 | [0.95, 1.55] | 0.124 | 1.14 | [0.87, 1.49] | 0.253 | 1.13 | [0.85, 1.49] | 0.277 | 1.13 | [0.86, 1.49] | 0.273 | 1.11 | [0.82, 1.51] | 0.319 | 1.09 | [0.78, 1.53] | 0.352 |
| **Head: Cobalt Chrome, Cup: non-HCLPE** | 0.59 | [0.39, 0.91] | 0.020 | 0.4 | [0.20, 0.80] | 0.014 | 0.41 | [0.23, 0.74] | 0.005 | 0.47 | [0.29, 0.74] | 0.003 | 0.42 | [0.26, 0.71] | 0.001 | 0.39 | [0.23, 0.68] | 0.001 |
| **Head: Delta Ceramic, Cup: non-HCLPE** | 0.47 | [0.30, 0.74] | 0.002 | 0.4 | [0.24, 0.66] | <0.001 | 0.39 | [0.23, 0.65] | <0.001 | 0.4 | [0.25, 0.65] | <0.001 | 0.38 | [0.23, 0.64] | <0.001 | 0.37 | [0.21, 0.64] | <0.001 |
| **Head: Alumina, Cup: non-HCLPE** | 0.66 | [0.46, 0.94] | 0.03 | 0.62 | [0.41, 0.94] | 0.031 | 0.61 | [0.40, 0.95] | 0.032 | 0.62 | [0.41, 0.94] | 0.031 | 0.61 | [0.38, 0.97] | 0.047 | 0.6 | [0.36, 1.00] | 0.058 |

Flexible parametric survival model adjusted for year of primary surgery, patient gender, age, Body Mass Index,  American Society of Anesthesiologists grade, implant fixation, stem composition and head size. HCLPE: Highly CrossLinked PolyethylenE.

Wald test

**Table L:** Monobloc acetabular component- Revision for implant wear Hazard Ratio (HR) and 95% Confidence Interval (CI) by time-point from primary procedure-Reference: Implant with cobalt chrome head and highly cross-linked polyethylene cup

|  | **6 months** | |  | **1 year** | |  | **2 years** | |  | **5 years** | |  | **10 years** | |  | **15 years** | |  |
| --- | --- | --- | --- | --- | --- | --- | --- | --- | --- | --- | --- | --- | --- | --- | --- | --- | --- | --- |
|  | **HR** | **95% CI** | **P-value** | **HR** | **95% CI** | **P-value** | **HR** | **95% CI** | **P-value** | **HR** | **95% CI** | **P-value** | **HR** | **95% CI** | **P-value** | **HR** | **95% CI** | **P-value** |
| **Head: Stainless Steel, Cup: HCLPE** | 1.00 | [0.42, 2.41] | 0.399 | 1.01 | [0.43, 2.38] | 0.399 | 1.01 | [0.37, 2.79] | 0.399 | 1.01 | [0.37, 2.77] | 0.399 | 1.01 | [0.37, 2.78] | 0.399 | 1.02 | [0.33, 3.09] | 0.399 |
| **Head: Delta Ceramic, Cup: HCLPE** | 0.61 | [0.23, 1.59] | 0.241 | 0.54 | [0.19, 1.51] | 0.202 | 0.48 | [0.14, 1.61] | 0.199 | 0.5 | [0.17, 1.52] | 0.185 | 0.51 | [0.17, 1.50] | 0.191 | 0.48 | [0.15, 1.55] | 0.187 |
| **Head: Stainless Steel, Cup: non-HCLPE** | 1.41 | [0.70, 2.83] | 0.251 | 1.48 | [0.74, 2.96] | 0.216 | 1.55 | [0.71, 3.38] | 0.218 | 1.56 | [0.72, 3.38] | 0.211 | 1.56 | [0.72, 3.39] | 0.212 | 1.6 | [0.69, 3.68] | 0.218 |
| **Head: Cobalt Chrome, Cup: non-HCLPE** | 2.17 | [1.11, 4.24] | 0.031 | 2.13 | [1.09, 4.15] | 0.034 | 2.09 | [0.98, 4.49] | 0.066 | 2.1 | [0.99, 4.46] | 0.062 | 2.1 | [0.99, 4.46] | 0.062 | 2.08 | [0.92, 4.71] | 0.085 |
| **Head: Delta Ceramic, Cup: non-HCLPE** | 1.4 | [0.61, 3.21] | 0.291 | 1.38 | [0.63, 3.04] | 0.289 | 1.37 | [0.57, 3.31] | 0.312 | 1.37 | [0.57, 3.28] | 0.311 | 1.37 | [0.57, 3.28] | 0.311 | 1.37 | [0.53, 3.52] | 0.323 |
| **Head: Alumina, Cup: non-HCLPE** | 0.29 | [0.04, 2.33] | 0.196 | 0.45 | [0.10, 2.07] | 0.234 | 0.67 | [0.21, 2.14] | 0.317 | 0.84 | [0.32, 2.20] | 0.375 | 0.95 | [0.36, 2.51] | 0.397 | 1.16 | [0.38, 3.52] | 0.386 |

Flexible parametric survival model adjusted for year of primary surgery, patient gender, age, Body Mass Index,  American Society of Anesthesiologists grade, implant fixation, stem composition and head size. HCLPE: Highly CrossLinked PolyethylenE.

Wald test

**Table M:** Monobloc acetabular component- Revision for malalignment Hazard Ratio (HR) and 95% Confidence Interval (CI) by time-point from primary procedure-Reference: Implant with cobalt chrome head and highly cross-linked polyethylene cup

|  | **6 months** | |  | **1 year** | |  | **2 years** | |  | **5 years** | |  | **10 years** | |  | **15 years** | |  |
| --- | --- | --- | --- | --- | --- | --- | --- | --- | --- | --- | --- | --- | --- | --- | --- | --- | --- | --- |
|  | **HR** | **95% CI** | **P-value** | **HR** | **95% CI** | **P-value** | **HR** | **95% CI** | **P-value** | **HR** | **95% CI** | **P-value** | **HR** | **95% CI** | **P-value** | **HR** | **95% CI** | **P-value** |
| **Head: Stainless Steel, Cup: HCLPE** | 0.78 | [0.37, 1.68] | 0.324 | 0.84 | [0.43, 1.67] | 0.351 | 0.81 | [0.40, 1.64] | 0.336 | 0.68 | [0.27, 1.72] | 0.286 | 0.63 | [0.21, 1.82] | 0.281 | 0.6 | [0.19, 1.91] | 0.274 |
| **Head: Delta Ceramic, Cup: HCLPE** | 1.01 | [0.47, 2.17] | 0.399 | 1.19 | [0.63, 2.25] | 0.346 | 1.13 | [0.59, 2.16] | 0.373 | 0.86 | [0.36, 2.03] | 0.376 | 0.77 | [0.29, 2.04] | 0.348 | 0.71 | [0.25, 2.06] | 0.326 |
| **Head: Alumina, Cup: HCLPE** |  |  |  | 0.47 | [0.11, 1.97] | 0.236 | 0.45 | [0.11, 1.77] | 0.211 | 0.27 | [0.04, 1.98] | 0.168 | 0.22 | [0.03, 1.99] | 0.147 | 0.2 | [0.02, 2.03] | 0.157 |
| **Head: Stainless Steel, Cup: non-HCLPE** | 1.43 | [0.84, 2.43] | 0.167 | 1.52 | [0.91, 2.54] | 0.111 | 1.46 | [0.86, 2.48] | 0.150 | 1.25 | [0.64, 2.46] | 0.323 | 1.16 | [0.53, 2.54] | 0.372 | 1.11 | [0.47, 2.60] | 0.388 |
| **Head: Cobalt Chrome, Cup: non-HCLPE** | 1.81 | [1.07, 3.06] | 0.034 | 1.93 | [1.17, 3.19] | 0.015 | 1.86 | [1.10, 3.12] | 0.026 | 1.58 | [0.80, 3.11] | 0.167 | 1.47 | [0.67, 3.23] | 0.252 | 1.41 | [0.59, 3.33] | 0.295 |
| **Head: Delta Ceramic, Cup: non-HCLPE** | 1.16 | [0.58, 2.30] | 0.365 | 1.18 | [0.60, 2.31] | 0.355 | 1.16 | [0.58, 2.31] | 0.365 | 1.09 | [0.45, 2.63] | 0.392 | 1.06 | [0.38, 2.97] | 0.396 | 1.04 | [0.34, 3.22] | 0.398 |
| **Head: Alumina, Cup: non-HCLPE** | 1.05 | [0.47, 2.34] | 0.396 | 1.04 | [0.48, 2.26] | 0.397 | 1.12 | [0.54, 2.33] | 0.381 | 1.34 | [0.56, 3.24] | 0.322 | 1.48 | [0.51, 4.30] | 0.308 | 1.58 | [0.48, 5.20] | 0.301 |

Flexible parametric survival model adjusted for year of primary surgery, patient gender, age, Body Mass Index,  American Society of Anesthesiologists grade, implant fixation, stem composition and head size. HCLPE: Highly CrossLinked PolyethylenE.

Wald test

**Table N:** Monobloc acetabular component- Revision for dislocation or subluxation Hazard Ratio (HR) and 95% Confidence Interval (CI) by time-point from primary procedure-Reference: Implant with cobalt chrome head and highly cross-linked polyethylene cup

|  | **6 months** | |  | **1 year** | |  | **2 years** | |  | **5 years** | |  | **10 years** | |  | **15 years** | |  |
| --- | --- | --- | --- | --- | --- | --- | --- | --- | --- | --- | --- | --- | --- | --- | --- | --- | --- | --- |
|  | **HR** | **95% CI** | **P-value** | **HR** | **95% CI** | **P-value** | **HR** | **95% CI** | **P-value** | **HR** | **95% CI** | **P-value** | **HR** | **95% CI** | **P-value** | **HR** | **95% CI** | **P-value** |
| **Head: Stainless Steel, Cup: HCLPE** | 0.8 | [0.58, 1.11] | 0.161 | 0.81 | [0.60, 1.11] | 0.162 | 0.78 | [0.56, 1.08] | 0.133 | 0.72 | [0.50, 1.04] | 0.085 | 0.69 | [0.47, 1.03] | 0.072 | 0.66 | [0.43, 1.01] | 0.065 |
| **Head: Delta Ceramic, Cup: HCLPE** | 0.5 | [0.32, 0.81] | 0.006 | 0.55 | [0.37, 0.82] | 0.005 | 0.52 | [0.35, 0.78] | 0.002 | 0.47 | [0.30, 0.72] | 0.001 | 0.44 | [0.28, 0.69] | <0.001 | 0.41 | [0.25, 0.67] | <0.001 |
| **Head: Alumina, Cup: HCLPE** | 0.72 | [0.42, 1.24] | 0.197 | 0.72 | [0.42, 1.24] | 0.197 | 0.71 | [0.40, 1.26] | 0.201 | 0.69 | [0.35, 1.33] | 0.220 | 0.67 | [0.32, 1.40] | 0.227 | 0.66 | [0.30, 1.48] | 0.237 |
| **Head: Stainless Steel, Cup: non-HCLPE** | 1 | [0.78, 1.27] | 0.399 | 1.02 | [0.79, 1.30] | 0.394 | 0.97 | [0.75, 1.26] | 0.389 | 0.9 | [0.67, 1.20] | 0.310 | 0.86 | [0.62, 1.17] | 0.259 | 0.82 | [0.58, 1.15] | 0.209 |
| **Head: Cobalt Chrome, Cup: non-HCLPE** | 1.12 | [0.87, 1.44] | 0.270 | 1.15 | [0.90, 1.47] | 0.214 | 1.1 | [0.85, 1.43] | 0.308 | 1.01 | [0.75, 1.35] | 0.398 | 0.96 | [0.70, 1.32] | 0.386 | 0.91 | [0.64, 1.28] | 0.346 |
| **Head: Delta Ceramic, Cup: non-HCLPE** | 1.01 | [0.73, 1.39] | 0.398 | 1.01 | [0.73, 1.39] | 0.398 | 0.99 | [0.71, 1.38] | 0.398 | 0.96 | [0.65, 1.40] | 0.390 | 0.94 | [0.62, 1.42] | 0.382 | 0.92 | [0.58, 1.45] | 0.374 |
| **Head: Alumina, Cup: non-HCLPE** | 0.73 | [0.51, 1.06] | 0.096 | 0.73 | [0.50, 1.06] | 0.104 | 0.72 | [0.49, 1.06] | 0.099 | 0.7 | [0.46, 1.09] | 0.107 | 0.7 | [0.43, 1.12] | 0.137 | 0.69 | [0.41, 1.16] | 0.15 |

Flexible parametric survival model adjusted for year of primary surgery, patient gender, age, Body Mass Index,  American Society of Anesthesiologists grade, implant fixation, stem composition and head size. HCLPE: Highly CrossLinked PolyethylenE.

Wald test

**Table O:** Monobloc acetabular component- Revision for pain Hazard Ratio (HR) and 95% Confidence Interval (CI) by time-point from primary procedure-Reference: Implant with cobalt chrome head and highly cross-linked polyethylene cup

|  | **6 months** | |  | **1 year** | |  | **2 years** | |  | **5 years** | |  | **10 years** | |  | **15 years** | |  |
| --- | --- | --- | --- | --- | --- | --- | --- | --- | --- | --- | --- | --- | --- | --- | --- | --- | --- | --- |
|  | **HR** | **95% CI** | **P-value** | **HR** | **95% CI** | **P-value** | **HR** | **95% CI** | **P-value** | **HR** | **95% CI** | **P-value** | **HR** | **95% CI** | **P-value** | **HR** | **95% CI** | **P-value** |
| **Head: Stainless Steel, Cup: HCLPE** | 1.26 | [0.54, 2.96] | 0.346 | 1.27 | [0.61, 2.66] | 0.326 | 1.29 | [0.66, 2.54] | 0.303 | 1.33 | [0.53, 3.33] | 0.332 | 1.39 | [0.20, 9.60] | 0.377 | 1.43 | [0.11, 18.65] | 0.384 |
| **Head: Delta Ceramic, Cup: HCLPE** | 1.05 | [0.45, 2.45] | 0.396 | 1.02 | [0.49, 2.12] | 0.398 | 0.99 | [0.51, 1.94] | 0.399 | 0.94 | [0.36, 2.47] | 0.396 | 0.83 | [0.09, 7.47] | 0.394 | 0.77 | [0.04, 15.84] | 0.393 |
| **Head: Alumina, Cup: HCLPE** | 1.71 | [0.55, 5.28] | 0.259 | 1.63 | [0.62, 4.28] | 0.244 | 1.51 | [0.64, 3.60] | 0.258 | 1.31 | [0.38, 4.56] | 0.364 | 0.94 | [0.03, 26.41] | 0.399 |  |  |  |
| **Head: Stainless Steel, Cup: non-HCLPE** | 2.23 | [1.12, 4.46] | 0.030 | 2.41 | [1.32, 4.38] | 0.006 | 2.66 | [1.54, 4.61] | <0.001 | 3.21 | [1.52, 6.79] | 0.004 | 4.4 | [0.90, 21.52] | 0.075 | 5.18 | [0.61, 43.64] | 0.128 |
| **Head: Cobalt Chrome, Cup: non-HCLPE** | 1.75 | [0.86, 3.58] | 0.122 | 1.98 | [1.08, 3.62] | 0.034 | 2.3 | [1.34, 3.96] | 0.004 | 3.05 | [1.45, 6.40] | 0.005 | 4.75 | [0.95, 23.69] | 0.065 | 5.94 | [0.68, 51.89] | 0.109 |
| **Head: Delta Ceramic, Cup: non-HCLPE** | 1.7 | [0.71, 4.06] | 0.196 | 1.81 | [0.87, 3.77] | 0.113 | 1.97 | [1.04, 3.74] | 0.046 | 2.32 | [1.02, 5.30] | 0.054 | 3.07 | [0.54, 17.43] | 0.179 | 3.55 | [0.35, 35.92] | 0.224 |
| **Head: Alumina, Cup: non-HCLPE** | 2.21 | [0.92, 5.31] | 0.083 | 2.23 | [1.05, 4.73] | 0.045 | 2.27 | [1.17, 4.40] | 0.021 | 2.34 | [1.00, 5.45] | 0.058 | 2.48 | [0.41, 14.92] | 0.244 | 2.57 | [0.23, 28.39] | 0.297 |

Flexible parametric survival model adjusted for year of primary surgery, patient gender, age, Body Mass Index,  American Society of Anesthesiologists grade, implant fixation, stem composition and head size. HCLPE: Highly CrossLinked PolyethylenE.

Wald test

**Table P:** Monobloc acetabular component- Revision for infection Hazard Ratio (HR) and 95% Confidence Interval (CI) by time-point from primary procedure-Reference: Implant with cobalt chrome head and highly cross-linked polyethylene cup

|  | **6 months** | |  | **1 year** | |  | **2 years** | |  | **5 years** | |  | **10 years** | |  | **15 years** | |  |
| --- | --- | --- | --- | --- | --- | --- | --- | --- | --- | --- | --- | --- | --- | --- | --- | --- | --- | --- |
|  | **HR** | **95% CI** | **P-value** | **HR** | **95% CI** | **P-value** | **HR** | **95% CI** | **P-value** | **HR** | **95% CI** | **P-value** | **HR** | **95% CI** | **P-value** | **HR** | **95% CI** | **P-value** |
| **Head: Stainless Steel, Cup: HCLPE** | 0.82 | [0.61, 1.10] | 0.167 | 0.84 | [0.61, 1.14] | 0.220 | 0.85 | [0.61, 1.19] | 0.253 | 0.91 | [0.57, 1.45] | 0.369 | 0.92 | [0.56, 1.49] | 0.377 | 0.93 | [0.55, 1.55] | 0.384 |
| **Head: Delta Ceramic, Cup: HCLPE** | 0.79 | [0.58, 1.09] | 0.136 | 0.77 | [0.55, 1.08] | 0.126 | 0.75 | [0.52, 1.08] | 0.121 | 0.68 | [0.40, 1.16] | 0.146 | 0.68 | [0.40, 1.16] | 0.146 | 0.67 | [0.38, 1.18] | 0.153 |
| **Head: Alumina, Cup: HCLPE** | 0.61 | [0.35, 1.04] | 0.082 | 0.73 | [0.45, 1.19] | 0.178 | 0.86 | [0.53, 1.41] | 0.332 | 1.29 | [0.66, 2.50] | 0.301 | 1.43 | [0.68, 3.03] | 0.257 | 1.58 | [0.69, 3.62] | 0.222 |
| **Head: Stainless Steel, Cup: non-HCLPE** | 1.22 | [0.96, 1.55] | 0.106 | 1.3 | [1.01, 1.67] | 0.049 | 1.36 | [1.04, 1.78] | 0.032 | 1.58 | [1.09, 2.27] | 0.020 | 1.61 | [1.11, 2.36] | 0.019 | 1.66 | [1.11, 2.48] | 0.019 |
| **Head: Cobalt Chrome, Cup: non-HCLPE** | 1.06 | [0.82, 1.36] | 0.360 | 1.16 | [0.89, 1.50] | 0.214 | 1.25 | [0.95, 1.65] | 0.114 | 1.55 | [1.06, 2.26] | 0.030 | 1.61 | [1.08, 2.39] | 0.025 | 1.69 | [1.10, 2.58] | 0.022 |
| **Head: Delta Ceramic, Cup: non-HCLPE** | 0.96 | [0.69, 1.35] | 0.388 | 0.98 | [0.69, 1.39] | 0.396 | 1 | [0.69, 1.45] | 0.399 | 1.05 | [0.63, 1.76] | 0.392 | 1.06 | [0.62, 1.81] | 0.390 | 1.07 | [0.60, 1.89] | 0.388 |
| **Head: Alumina, Cup: non-HCLPE** | 0.86 | [0.58, 1.27] | 0.3 | 0.98 | [0.68, 1.41] | 0.397 | 1.1 | [0.76, 1.59] | 0.351 | 1.48 | [0.92, 2.41] | 0.112 | 1.59 | [0.94, 2.68] | 0.089 | 1.7 | [0.96, 3.00] | 0.075 |

Flexible parametric survival model adjusted for year of primary surgery, patient gender, age, Body Mass Index,  American Society of Anesthesiologists grade, implant fixation, stem composition and head size. HCLPE: Highly CrossLinked PolyethylenE.

Wald test

**Table Q:** Monobloc acetabular component- Revision for any other reason(s) Hazard Ratio (HR) and 95% Confidence Interval (CI) by time-point from primary procedure-Reference: Implant with cobalt chrome head and highly cross-linked polyethylene cup

|  | **6 months** | |  | **1 year** | |  | **2 years** | |  | **5 years** | |  | **10 years** | |  | **15 years** | |  |
| --- | --- | --- | --- | --- | --- | --- | --- | --- | --- | --- | --- | --- | --- | --- | --- | --- | --- | --- |
|  | **HR** | **95% CI** | **P-value** | **HR** | **95% CI** | **P-value** | **HR** | **95% CI** | **P-value** | **HR** | **95% CI** | **P-value** | **HR** | **95% CI** | **P-value** | **HR** | **95% CI** | **P-value** |
| **Head: Stainless Steel, Cup: HCLPE** | 0.53 | [0.29, 0.97] | 0.048 | 0.64 | [0.39, 1.05] | 0.084 | 0.76 | [0.47, 1.24] | 0.216 | 0.87 | [0.50, 1.51] | 0.353 | 0.94 | [0.51, 1.75] | 0.391 | 1.03 | [0.51, 2.10] | 0.398 |
| **Head: Delta Ceramic, Cup: HCLPE** | 0.68 | [0.42, 1.11] | 0.119 | 0.71 | [0.45, 1.12] | 0.135 | 0.73 | [0.44, 1.20] | 0.187 | 0.74 | [0.43, 1.29] | 0.224 | 0.75 | [0.42, 1.35] | 0.250 | 0.77 | [0.40, 1.47] | 0.293 |
| **Head: Alumina, Cup: HCLPE** | 0.85 | [0.41, 1.80] | 0.364 | 0.96 | [0.52, 1.77] | 0.396 | 1.08 | [0.59, 1.96] | 0.387 | 1.17 | [0.60, 2.27] | 0.358 | 1.22 | [0.59, 2.55] | 0.346 | 1.3 | [0.57, 2.99] | 0.329 |
| **Head: Stainless Steel, Cup: non-HCLPE** | 1.36 | [0.94, 1.97] | 0.106 | 1.38 | [0.97, 1.97] | 0.082 | 1.4 | [0.96, 2.05] | 0.088 | 1.41 | [0.94, 2.12] | 0.101 | 1.42 | [0.93, 2.18] | 0.109 | 1.43 | [0.90, 2.28] | 0.128 |
| **Head: Cobalt Chrome, Cup: non-HCLPE** | 1.65 | [1.15, 2.37] | 0.010 | 1.66 | [1.17, 2.34] | 0.007 | 1.66 | [1.15, 2.41] | 0.011 | 1.67 | [1.12, 2.49] | 0.017 | 1.67 | [1.10, 2.55] | 0.023 | 1.68 | [1.06, 2.66] | 0.035 |
| **Head: Delta Ceramic, Cup: non-HCLPE** | 0.93 | [0.57, 1.52] | 0.383 | 0.88 | [0.55, 1.40] | 0.345 | 0.84 | [0.51, 1.37] | 0.314 | 0.82 | [0.48, 1.38] | 0.304 | 0.81 | [0.47, 1.40] | 0.300 | 0.79 | [0.43, 1.43] | 0.297 |
| **Head: Alumina, Cup: non-HCLPE** | 0.83 | [0.45, 1.50] | 0.332 | 0.88 | [0.53, 1.45] | 0.352 | 0.93 | [0.57, 1.51] | 0.382 | 0.97 | [0.58, 1.61] | 0.396 | 0.99 | [0.57, 1.70] | 0.399 | 1.02 | [0.56, 1.86] | 0.398 |

Flexible parametric survival model adjusted for year of primary surgery, patient gender, age, Body Mass Index,  American Society of Anesthesiologists grade, implant fixation, stem composition and head size. HCLPE: Highly CrossLinked PolyethylenE.

Wald test

**Table R:** Modular acetabular component- All-cause revision Hazard Ratio (HR) and 95% Confidence Interval (CI) by time-point from primary procedure-Reference: Implant with cobalt chrome head and highly cross-linked polyethylene liner

|  | **6 months** | |  | **1 year** | |  | **2 years** | |  | **5 years** | |  | **10 years** | |  | **15 years** | |  |
| --- | --- | --- | --- | --- | --- | --- | --- | --- | --- | --- | --- | --- | --- | --- | --- | --- | --- | --- |
|  | **HR** | **95% CI** | **P-value** | **HR** | **95% CI** | **P-value** | **HR** | **95% CI** | **P-value** | **HR** | **95% CI** | **P-value** | **HR** | **95% CI** | **P-value** | **HR** | **95% CI** | **P-value** |
| **Head: Alumina, Liner: Alumina** | 1.08 | [0.99, 1.18] | 0.091 | 1.09 | [1.00, 1.18] | 0.050 | 1.14 | [1.05, 1.24] | 0.003 | 1.24 | [1.13, 1.36] | <0.001 | 1.24 | [1.13, 1.36] | <0.001 | 1.28 | [1.16, 1.42] | <0.001 |
| **Head: Alumina, Liner: HCLPE** | 0.91 | [0.81, 1.02] | 0.110 | 0.91 | [0.81, 1.01] | 0.098 | 0.92 | [0.82, 1.03] | 0.143 | 0.94 | [0.83, 1.08] | 0.261 | 0.94 | [0.82, 1.08] | 0.271 | 0.95 | [0.83, 1.10] | 0.309 |
| **Head: Alumina, Liner: non-HCLPE** | 1.32 | [1.09, 1.59] | 0.006 | 1.33 | [1.11, 1.59] | 0.003 | 1.42 | [1.21, 1.68] | <0.001 | 1.59 | [1.32, 1.91] | <0.001 | 1.60 | [1.32, 1.93] | <0.001 | 1.67 | [1.36, 2.06] | <0.001 |
| **Head: Delta Ceramic, Liner: Delta Ceramic** | 0.92 | [0.86, 0.97] | 0.010 | 0.91 | [0.86, 0.97] | 0.004 | 0.93 | [0.88, 0.99] | 0.022 | 0.97 | [0.90, 1.04] | 0.284 | 0.96 | [0.90, 1.03] | 0.197 | 0.98 | [0.91, 1.05] | 0.342 |
| **Head: Delta Ceramic, Liner: HCLPE** | 0.78 | [0.73, 0.84] | <0.001 | 0.78 | [0.73, 0.84] | <0.001 | 0.79 | [0.73, 0.84] | <0.001 | 0.79 | [0.73, 0.86] | <0.001 | 0.79 | [0.73, 0.85] | <0.001 | 0.79 | [0.73, 0.86] | <0.001 |
| **Head: Delta Ceramic, Liner: non-HCLPE** | 0.82 | [0.74, 0.92] | <0.001 | 0.82 | [0.73, 0.92] | 0.001 | 0.84 | [0.75, 0.94] | 0.004 | 0.87 | [0.77, 1.00] | 0.045 | 0.87 | [0.77, 0.99] | 0.038 | 0.89 | [0.77, 1.02] | 0.107 |
| **Head: Oxidised Zirconium, Liner: HCLPE** | 0.66 | [0.57, 0.77] | <0.001 | 0.67 | [0.58, 0.77] | <0.001 | 0.66 | [0.57, 0.77] | <0.001 | 0.65 | [0.54, 0.77] | <0.001 | 0.65 | [0.55, 0.77] | <0.001 | 0.64 | [0.54, 0.76] | <0.001 |
| **Head: Cobalt Chrome, Liner: non-HCLPE** | 1.30 | [1.22, 1.39] | <0.001 | 1.30 | [1.22, 1.39] | <0.001 | 1.35 | [1.26, 1.45] | <0.001 | 1.44 | [1.33, 1.56] | <0.001 | 1.44 | [1.33, 1.55] | <0.001 | 1.48 | [1.36, 1.61] | <0.001 |
| **Head: Stainless Steel, Liner: HCLPE** | 0.98 | [0.88, 1.09] | 0.373 | 0.98 | [0.88, 1.09] | 0.373 | 0.98 | [0.87, 1.10] | 0.377 | 0.98 | [0.86, 1.12] | 0.381 | 0.98 | [0.87, 1.11] | 0.378 | 0.98 | [0.86, 1.12] | 0.381 |
| **Head: Stainless Steel, Liner: non-HCLPE** | 1.40 | [1.26, 1.55] | <0.001 | 1.40 | [1.26, 1.55] | <0.001 | 1.48 | [1.34, 1.64] | <0.001 | 1.62 | [1.44, 1.81] | <0.001 | 1.62 | [1.44, 1.82] | <0.001 | 1.68 | [1.48, 1.90] | <0.001 |
| **Head: Delta Ceramic, Pre-assembled implant** | 0.52 | [0.40, 0.68] | <0.001 | 0.52 | [0.40, 0.68] | <0.001 | 0.53 | [0.40, 0.70] | <0.001 | 0.54 | [0.39, 0.74] | <0.001 | 0.54 | [0.39, 0.73] | <0.001 | 0.54 | [0.39, 0.76] | <0.001 |
| **Head: Cobalt Chrome or Stainless Steel, Pre-assembled implant** | 1.20 | [0.85, 1.69] | 0.232 | 1.23 | [0.90, 1.67] | 0.169 | 1.2 | [0.87, 1.66] | 0.216 | 1.15 | [0.80, 1.65] | 0.300 | 1.17 | [0.83, 1.64] | 0.265 | 1.15 | [0.80, 1.64] | 0.298 |

Flexible parametric survival model adjusted for year of primary surgery, patient gender, age, Body Mass Index,  American Society of Anesthesiologists grade, implant fixation, shell composition, stem composition and head size. HCLPE: Highly CrossLinked PolyethylenE.

Wald test

**Table S:** Modular acetabular component- Revision for aseptic loosening Hazard Ratio (HR) and 95% Confidence Interval (CI) by time-point from primary procedure-Reference: Implant with cobalt chrome head and highly cross-linked polyethylene liner

|  | **6 months** | |  | **1 year** | |  | **2 years** | |  | **5 years** | |  | **10 years** | |  | **15 years** | |  |
| --- | --- | --- | --- | --- | --- | --- | --- | --- | --- | --- | --- | --- | --- | --- | --- | --- | --- | --- |
|  | **HR** | **95% CI** | **P-value** | **HR** | **95% CI** | **P-value** | **HR** | **95% CI** | **P-value** | **HR** | **95% CI** | **P-value** | **HR** | **95% CI** | **P-value** | **HR** | **95% CI** | **P-value** |
| **Head: Alumina, Liner: Alumina** | 1.31 | [1.03, 1.66] | 0.034 | 1.41 | [1.16, 1.71] | <0.001 | 1.53 | [1.29, 1.81] | <0.001 | 1.75 | [1.44, 2.14] | <0.001 | 1.79 | [1.45, 2.21] | <0.001 | 1.87 | [1.48, 2.36] | <0.001 |
| **Head: Alumina, Liner: HCLPE** | 1.01 | [0.75, 1.36] | 0.398 | 1.02 | [0.79, 1.31] | 0.394 | 1.02 | [0.79, 1.31] | 0.394 | 1.03 | [0.74, 1.44] | 0.393 | 1.03 | [0.73, 1.46] | 0.393 | 1.03 | [0.71, 1.51] | 0.394 |
| **Head: Alumina, Liner: non-HCLPE** | 1.43 | [0.75, 2.70] | 0.219 | 1.71 | [1.05, 2.80] | 0.040 | 2.09 | [1.45, 3.01] | <0.001 | 2.90 | [2.09, 4.00] | <0.001 | 3.13 | [2.17, 4.53] | <0.001 | 3.46 | [2.25, 5.31] | <0.001 |
| **Head: Delta Ceramic, Liner: Delta Ceramic** | 0.78 | [0.67, 0.91] | 0.003 | 0.86 | [0.76, 0.98] | 0.027 | 0.96 | [0.85, 1.09] | 0.324 | 1.16 | [0.99, 1.35] | 0.069 | 1.20 | [1.02, 1.41] | 0.035 | 1.27 | [1.06, 1.52] | 0.014 |
| **Head: Delta Ceramic, Liner: HCLPE** | 0.54 | [0.45, 0.64] | <0.001 | 0.59 | [0.50, 0.68] | <0.001 | 0.65 | [0.55, 0.76] | <0.001 | 0.76 | [0.61, 0.95] | 0.021 | 0.78 | [0.62, 0.99] | 0.046 | 0.82 | [0.63, 1.07] | 0.136 |
| **Head: Delta Ceramic, Liner: non-HCLPE** | 1.10 | [0.81, 1.49] | 0.331 | 1.12 | [0.87, 1.44] | 0.270 | 1.13 | [0.91, 1.42] | 0.223 | 1.17 | [0.90, 1.52] | 0.200 | 1.17 | [0.89, 1.54] | 0.212 | 1.18 | [0.87, 1.59] | 0.224 |
| **Head: Oxidised Zirconium, Liner: HCLPE** | 0.46 | [0.33, 0.65] | <0.001 | 0.42 | [0.30, 0.60] | <0.001 | 0.37 | [0.25, 0.55] | <0.001 | 0.30 | [0.18, 0.52] | <0.001 | 0.31 | [0.18, 0.51] | <0.001 | 0.29 | [0.17, 0.50] | <0.001 |
| **Head: Cobalt Chrome, Liner: non-HCLPE** | 1.61 | [1.32, 1.97] | <0.001 | 1.89 | [1.60, 2.22] | <0.001 | 2.24 | [1.94, 2.57] | <0.001 | 2.96 | [2.51, 3.48] | <0.001 | 3.15 | [2.63, 3.77] | <0.001 | 3.43 | [2.80, 4.19] | <0.001 |
| **Head: Stainless Steel, Liner: HCLPE** | 0.86 | [0.59, 1.23] | 0.289 | 0.84 | [0.60, 1.18] | 0.239 | 0.82 | [0.58, 1.17] | 0.216 | 0.79 | [0.49, 1.27] | 0.249 | 0.79 | [0.49, 1.28] | 0.251 | 0.78 | [0.46, 1.32] | 0.260 |
| **Head: Stainless Steel, Liner: non-HCLPE** | 2.27 | [1.59, 3.23] | <0.001 | 2.64 | [1.98, 3.52] | <0.001 | 3.12 | [2.46, 3.96] | <0.001 | 4.10 | [3.20, 5.26] | <0.001 | 4.36 | [3.33, 5.72] | <0.001 | 4.74 | [3.50, 6.41] | <0.001 |
| **Head: Delta Ceramic, Pre-assembled implant** | 0.47 | [0.26, 0.87] | 0.020 | 0.49 | [0.29, 0.82] | 0.011 | 0.51 | [0.31, 0.83] | 0.011 | 0.54 | [0.29, 1.01] | 0.061 | 0.54 | [0.28, 1.05] | 0.075 | 0.55 | [0.26, 1.14] | 0.114 |
| **Head: Cobalt Chrome or Stainless Steel, Pre-assembled implant** | 2.46 | [1.35, 4.48] | 0.005 | 2.51 | [1.48, 4.27] | 0.001 | 2.57 | [1.54, 4.30] | <0.001 | 2.68 | [1.40, 5.11] | 0.005 | 2.69 | [1.37, 5.28] | 0.006 | 2.72 | [1.30, 5.69] | 0.012 |

Flexible parametric survival model adjusted for year of primary surgery, patient gender, age, Body Mass Index,  American Society of Anesthesiologists grade, implant fixation, shell composition, stem composition and head size. HCLPE: Highly CrossLinked PolyethylenE.

Wald test

**Table T:** Modular acetabular component- Revision for peri-prosthetic fracture Hazard Ratio (HR) and 95% Confidence Interval (CI) by time-point from primary procedure-Reference: Implant with cobalt chrome head and highly cross-linked polyethylene liner

|  | **6 months** | |  | **1 year** | |  | **2 years** | |  | **5 years** | |  | **10 years** | |  | **15 years** | |  |
| --- | --- | --- | --- | --- | --- | --- | --- | --- | --- | --- | --- | --- | --- | --- | --- | --- | --- | --- |
|  | **HR** | **95% CI** | **P-value** | **HR** | **95% CI** | **P-value** | **HR** | **95% CI** | **P-value** | **HR** | **95% CI** | **P-value** | **HR** | **95% CI** | **P-value** | **HR** | **95% CI** | **P-value** |
| **Head: Alumina, Liner: Alumina** | 1.17 | [0.94, 1.45] | 0.146 | 1.19 | [0.98, 1.46] | 0.092 | 1.22 | [0.99, 1.49] | 0.065 | 1.25 | [0.99, 1.58] | 0.069 | 1.28 | [0.97, 1.68] | 0.085 | 1.30 | [0.96, 1.75] | 0.092 |
| **Head: Alumina, Liner: HCLPE** | 0.90 | [0.70, 1.16] | 0.286 | 0.89 | [0.69, 1.15] | 0.267 | 0.88 | [0.67, 1.16] | 0.263 | 0.87 | [0.62, 1.21] | 0.286 | 0.86 | [0.58, 1.26] | 0.298 | 0.85 | [0.56, 1.30] | 0.300 |
| **Head: Alumina, Liner: non-HCLPE** | 1.01 | [0.55, 1.85] | 0.399 | 1.12 | [0.68, 1.85] | 0.362 | 1.24 | [0.79, 1.96] | 0.259 | 1.43 | [0.87, 2.38] | 0.151 | 1.60 | [0.86, 2.97] | 0.132 | 1.70 | [0.85, 3.43] | 0.131 |
| **Head: Delta Ceramic, Liner: Delta Ceramic** | 0.80 | [0.69, 0.91] | 0.003 | 0.74 | [0.65, 0.85] | <0.001 | 0.69 | [0.60, 0.81] | <0.001 | 0.63 | [0.53, 0.75] | <0.001 | 0.59 | [0.48, 0.72] | <0.001 | 0.57 | [0.46, 0.70] | <0.001 |
| **Head: Delta Ceramic, Liner: HCLPE** | 0.79 | [0.70, 0.90] | <0.001 | 0.77 | [0.67, 0.88] | <0.001 | 0.75 | [0.65, 0.88] | <0.001 | 0.73 | [0.61, 0.88] | 0.001 | 0.71 | [0.57, 0.88] | 0.003 | 0.70 | [0.56, 0.88] | 0.003 |
| **Head: Delta Ceramic, Liner: non-HCLPE** | 0.59 | [0.42, 0.84] | 0.005 | 0.58 | [0.41, 0.80] | 0.002 | 0.56 | [0.40, 0.80] | 0.002 | 0.54 | [0.36, 0.82] | 0.005 | 0.53 | [0.33, 0.85] | 0.013 | 0.52 | [0.31, 0.87] | 0.018 |
| **Head: Oxidised Zirconium, Liner: HCLPE** | 0.66 | [0.49, 0.90] | 0.011 | 0.58 | [0.42, 0.80] | 0.002 | 0.51 | [0.36, 0.73] | <0.001 | 0.43 | [0.28, 0.65] | <0.001 | 0.37 | [0.24, 0.59] | <0.001 | 0.35 | [0.21, 0.56] | <0.001 |
| **Head: Cobalt Chrome, Liner: non-HCLPE** | 1.03 | [0.87, 1.22] | 0.376 | 1.15 | [0.99, 1.34] | 0.078 | 1.27 | [1.09, 1.47] | 0.003 | 1.46 | [1.23, 1.73] | <0.001 | 1.62 | [1.32, 1.99] | <0.001 | 1.72 | [1.38, 2.16] | <0.001 |
| **Head: Stainless Steel, Liner: HCLPE** | 0.80 | [0.64, 1.00] | 0.058 | 0.89 | [0.72, 1.11] | 0.229 | 0.99 | [0.78, 1.24] | 0.398 | 1.14 | [0.86, 1.51] | 0.263 | 1.27 | [0.90, 1.78] | 0.155 | 1.35 | [0.93, 1.96] | 0.115 |
| **Head: Stainless Steel, Liner: non-HCLPE** | 0.60 | [0.40, 0.90] | 0.019 | 0.85 | [0.62, 1.16] | 0.238 | 1.17 | [0.91, 1.51] | 0.191 | 1.83 | [1.43, 2.34] | <0.001 | 2.57 | [1.91, 3.47] | <0.001 | 3.13 | [2.22, 4.42] | <0.001 |
| **Head: Delta Ceramic, Pre-assembled implant** | 0.48 | [0.24, 0.98] | 0.049 | 0.45 | [0.21, 0.94] | 0.045 | 0.42 | [0.19, 0.94] | 0.042 | 0.38 | [0.15, 0.97] | 0.051 | 0.36 | [0.12, 1.03] | 0.070 | 0.34 | [0.11, 1.07] | 0.071 |
| **Head: Cobalt Chrome or Stainless Steel, Pre-assembled implant** | 1.34 | [0.74, 2.43] | 0.250 | 1.29 | [0.69, 2.39] | 0.289 | 1.24 | [0.63, 2.44] | 0.329 | 1.18 | [0.53, 2.63] | 0.368 | 1.14 | [0.46, 2.84] | 0.383 | 1.12 | [0.42, 3.00] | 0.389 |

Flexible parametric survival model adjusted for year of primary surgery, patient gender, age, Body Mass Index,  American Society of Anesthesiologists grade, implant fixation, shell composition, stem composition and head size. HCLPE: Highly CrossLinked PolyethylenE.

Wald test

**Table U:** Modular acetabular component- Revision for implant wear Hazard Ratio (HR) and 95% Confidence Interval (CI) by time-point from primary procedure-Reference: Implant with cobalt chrome head and highly cross-linked polyethylene liner

|  | **6 months** | |  | **1 year** | |  | **2 years** | |  | **5 years** | |  | **10 years** | |  | **15 years** | |  |
| --- | --- | --- | --- | --- | --- | --- | --- | --- | --- | --- | --- | --- | --- | --- | --- | --- | --- | --- |
|  | **HR** | **95% CI** | **P-value** | **HR** | **95% CI** | **P-value** | **HR** | **95% CI** | **P-value** | **HR** | **95% CI** | **P-value** | **HR** | **95% CI** | **P-value** | **HR** | **95% CI** | **P-value** |
| **Head: Alumina, Liner: Alumina** | 0.98 | [0.70, 1.37] | 0.396 | 0.99 | [0.69, 1.42] | 0.398 | 0.99 | [0.70, 1.41] | 0.398 | 0.99 | [0.69, 1.42] | 0.398 | 0.99 | [0.69, 1.42] | 0.398 | 0.99 | [0.69, 1.44] | 0.398 |
| **Head: Alumina, Liner: HCLPE** | 0.77 | [0.47, 1.27] | 0.235 | 0.77 | [0.44, 1.37] | 0.266 | 0.77 | [0.44, 1.36] | 0.264 | 0.77 | [0.44, 1.36] | 0.264 | 0.77 | [0.44, 1.36] | 0.264 | 0.77 | [0.43, 1.39] | 0.272 |
| **Head: Alumina, Liner: non-HCLPE** | 2.49 | [1.41, 4.41] | 0.003 | 2.81 | [1.77, 4.45] | <0.001 | 2.84 | [1.81, 4.47] | <0.001 | 2.90 | [1.82, 4.62] | <0.001 | 2.93 | [1.81, 4.73] | <0.001 | 3.00 | [1.80, 5.00] | <0.001 |
| **Head: Delta Ceramic, Liner: Delta Ceramic** | 0.66 | [0.52, 0.84] | 0.001 | 0.64 | [0.48, 0.84] | 0.003 | 0.64 | [0.49, 0.84] | 0.002 | 0.64 | [0.49, 0.84] | 0.002 | 0.64 | [0.49, 0.84] | 0.002 | 0.64 | [0.48, 0.84] | 0.003 |
| **Head: Delta Ceramic, Liner: HCLPE** | 0.74 | [0.56, 0.96] | 0.036 | 0.80 | [0.58, 1.09] | 0.153 | 0.80 | [0.58, 1.10] | 0.157 | 0.81 | [0.58, 1.12] | 0.181 | 0.81 | [0.58, 1.13] | 0.185 | 0.82 | [0.58, 1.17] | 0.216 |
| **Head: Delta Ceramic, Liner: non-HCLPE** | 0.55 | [0.23, 1.30] | 0.160 | 0.88 | [0.47, 1.64] | 0.368 | 1.08 | [0.69, 1.70] | 0.377 | 1.40 | [0.98, 1.99] | 0.070 | 1.62 | [1.10, 2.38] | 0.020 | 1.86 | [1.20, 2.89] | 0.009 |
| **Head: Oxidised Zirconium, Liner: HCLPE** | 0.47 | [0.24, 0.95] | 0.039 | 0.41 | [0.17, 1.03] | 0.061 | 0.43 | [0.20, 0.95] | 0.042 | 0.44 | [0.22, 0.91] | 0.031 | 0.45 | [0.22, 0.90] | 0.034 | 0.44 | [0.21, 0.90] | 0.035 |
| **Head: Cobalt Chrome, Liner: non-HCLPE** | 1.51 | [1.05, 2.16] | 0.032 | 2.22 | [1.64, 3.01] | <0.001 | 2.57 | [1.99, 3.31] | <0.001 | 3.08 | [2.40, 3.95] | <0.001 | 3.41 | [2.61, 4.46] | <0.001 | 3.79 | [2.84, 5.06] | <0.001 |
| **Head: Stainless Steel, Liner: HCLPE** | 0.76 | [0.43, 1.34] | 0.255 | 0.85 | [0.46, 1.55] | 0.348 | 0.85 | [0.46, 1.60] | 0.350 | 0.87 | [0.44, 1.69] | 0.367 | 0.87 | [0.44, 1.75] | 0.369 | 0.89 | [0.43, 1.86] | 0.380 |
| **Head: Stainless Steel, Liner: non-HCLPE** | 1.22 | [0.59, 2.54] | 0.346 | 1.99 | [1.14, 3.47] | 0.021 | 2.46 | [1.59, 3.82] | <0.001 | 3.22 | [2.20, 4.71] | <0.001 | 3.76 | [2.51, 5.62] | <0.001 | 4.33 | [2.79, 6.73] | <0.001 |
| **Head: Delta Ceramic, Pre-assembled implant** | 0.26 | [0.07, 0.98] | 0.054 | 0.21 | [0.04, 1.07] | 0.071 | 0.23 | [0.06, 0.93] | 0.044 | 0.24 | [0.07, 0.89] | 0.035 | 0.25 | [0.07, 0.88] | 0.040 | 0.24 | [0.07, 0.87] | 0.034 |
| **Head: Cobalt Chrome or Stainless Steel, Pre-assembled implant** | 0.26 | [0.01, 5.61] | 0.282 | 0.37 | [0.04, 3.62] | 0.274 | 0.42 | [0.05, 3.19] | 0.285 | 0.49 | [0.06, 4.11] | 0.321 | 0.53 | [0.05, 5.74] | 0.348 | 0.59 | [0.04, 8.12] | 0.370 |

Flexible parametric survival model adjusted for year of primary surgery, patient gender, age, Body Mass Index,  American Society of Anesthesiologists grade, implant fixation, shell composition, stem composition and head size. HCLPE: Highly CrossLinked PolyethylenE.

Wald test

**Table V:** Modular acetabular component- Revision for malalignment Hazard Ratio (HR) and 95% Confidence Interval (CI) by time-point from primary procedure-Reference: Implant with cobalt chrome head and highly cross-linked polyethylene liner

|  | **6 months** | |  | **1 year** | |  | **2 years** | |  | **5 years** | |  | **10 years** | |  | **15 years** | |  |
| --- | --- | --- | --- | --- | --- | --- | --- | --- | --- | --- | --- | --- | --- | --- | --- | --- | --- | --- |
|  | **HR** | **95% CI** | **P-value** | **HR** | **95% CI** | **P-value** | **HR** | **95% CI** | **P-value** | **HR** | **95% CI** | **P-value** | **HR** | **95% CI** | **P-value** | **HR** | **95% CI** | **P-value** |
| **Head: Alumina, Liner: Alumina** | 1.45 | [1.10, 1.91] | 0.012 | 1.35 | [1.04, 1.75] | 0.031 | 1.36 | [1.06, 1.76] | 0.024 | 1.75 | [1.29, 2.39] | <0.001 | 1.77 | [1.29, 2.44] | <0.001 | 1.88 | [1.33, 2.66] | <0.001 |
| **Head: Alumina, Liner: HCLPE** | 0.45 | [0.26, 0.78] | 0.007 | 0.43 | [0.26, 0.72] | 0.002 | 0.45 | [0.27, 0.74] | 0.003 | 0.67 | [0.35, 1.28] | 0.192 | 0.69 | [0.34, 1.43] | 0.239 | 0.77 | [0.34, 1.73] | 0.327 |
| **Head: Alumina, Liner: non-HCLPE** | 1.26 | [0.69, 2.29] | 0.300 | 1.18 | [0.65, 2.14] | 0.344 | 1.18 | [0.65, 2.14] | 0.344 | 1.41 | [0.73, 2.75] | 0.238 | 1.41 | [0.71, 2.78] | 0.245 | 1.47 | [0.71, 3.04] | 0.233 |
| **Head: Delta Ceramic, Liner: Delta Ceramic** | 1.02 | [0.82, 1.26] | 0.392 | 0.96 | [0.79, 1.17] | 0.367 | 0.96 | [0.79, 1.17] | 0.367 | 1.08 | [0.85, 1.38] | 0.329 | 1.08 | [0.85, 1.37] | 0.327 | 1.11 | [0.86, 1.42] | 0.286 |
| **Head: Delta Ceramic, Liner: HCLPE** | 0.74 | [0.58, 0.95] | 0.023 | 0.70 | [0.55, 0.87] | 0.004 | 0.70 | [0.55, 0.87] | 0.004 | 0.81 | [0.60, 1.09] | 0.153 | 0.81 | [0.60, 1.09] | 0.153 | 0.83 | [0.60, 1.15] | 0.212 |
| **Head: Delta Ceramic, Liner: non-HCLPE** | 1.32 | [0.96, 1.84] | 0.098 | 1.26 | [0.92, 1.72] | 0.140 | 1.25 | [0.92, 1.71] | 0.147 | 1.39 | [0.95, 2.04] | 0.096 | 1.39 | [0.95, 2.03] | 0.094 | 1.42 | [0.94, 2.13] | 0.097 |
| **Head: Oxidised Zirconium, Liner: HCLPE** | 0.77 | [0.47, 1.25] | 0.231 | 0.74 | [0.48, 1.14] | 0.157 | 0.74 | [0.48, 1.13] | 0.154 | 0.78 | [0.45, 1.38] | 0.273 | 0.78 | [0.45, 1.36] | 0.271 | 0.79 | [0.44, 1.43] | 0.293 |
| **Head: Cobalt Chrome, Liner: non-HCLPE** | 1.51 | [1.19, 1.92] | 0.001 | 1.46 | [1.17, 1.81] | 0.001 | 1.45 | [1.17, 1.81] | 0.002 | 1.55 | [1.19, 2.01] | 0.002 | 1.54 | [1.19, 1.99] | 0.002 | 1.56 | [1.19, 2.05] | 0.002 |
| **Head: Stainless Steel, Liner: HCLPE** | 0.81 | [0.51, 1.30] | 0.270 | 0.78 | [0.51, 1.19] | 0.206 | 0.78 | [0.51, 1.19] | 0.206 | 0.83 | [0.49, 1.43] | 0.316 | 0.83 | [0.49, 1.42] | 0.315 | 0.84 | [0.48, 1.49] | 0.333 |
| **Head: Stainless Steel, Liner: non-HCLPE** | 1.22 | [0.78, 1.93] | 0.276 | 1.31 | [0.90, 1.91] | 0.148 | 1.32 | [0.91, 1.92] | 0.138 | 1.22 | [0.79, 1.89] | 0.268 | 1.24 | [0.82, 1.88] | 0.238 | 1.22 | [0.79, 1.88] | 0.266 |
| **Head: Delta Ceramic, Pre-assembled implant** | 0.92 | [0.41, 2.08] | 0.391 | 0.86 | [0.38, 1.92] | 0.373 | 0.87 | [0.38, 1.96] | 0.377 | 1.10 | [0.41, 2.98] | 0.392 | 1.11 | [0.39, 3.13] | 0.391 | 1.17 | [0.38, 3.62] | 0.384 |
| **Head: Cobalt Chrome or Stainless Steel, Pre-assembled implant** | 1.75 | [0.57, 5.34] | 0.247 | 1.67 | [0.60, 4.66] | 0.247 | 1.66 | [0.59, 4.66] | 0.251 | 1.82 | [0.47, 7.06] | 0.274 | 1.81 | [0.47, 7.01] | 0.275 | 1.84 | [0.43, 7.93] | 0.285 |

Flexible parametric survival model adjusted for year of primary surgery, patient gender, age, Body Mass Index,  American Society of Anesthesiologists grade, implant fixation, shell composition, stem composition and head size. HCLPE: Highly CrossLinked PolyethylenE.

Wald test

**Table W:** Modular acetabular component- Revision for dislocation or subluxation Hazard Ratio (HR) and 95% Confidence Interval (CI) by time-point from primary procedure-Reference: Implant with cobalt chrome head and highly cross-linked polyethylene liner

|  | **6 months** | |  | **1 year** | |  | **2 years** | |  | **5 years** | |  | **10 years** | |  | **15 years** | |  |
| --- | --- | --- | --- | --- | --- | --- | --- | --- | --- | --- | --- | --- | --- | --- | --- | --- | --- | --- |
|  | **HR** | **95% CI** | **P-value** | **HR** | **95% CI** | **P-value** | **HR** | **95% CI** | **P-value** | **HR** | **95% CI** | **P-value** | **HR** | **95% CI** | **P-value** | **HR** | **95% CI** | **P-value** |
| **Head: Alumina, Liner: Alumina** | 0.64 | [0.51, 0.80] | <0.001 | 0.70 | [0.56, 0.87] | 0.003 | 0.76 | [0.60, 0.96] | 0.029 | 0.85 | [0.65, 1.12] | 0.201 | 0.92 | [0.67, 1.27] | 0.350 | 0.96 | [0.68, 1.37] | 0.389 |
| **Head: Alumina, Liner: HCLPE** | 0.88 | [0.70, 1.11] | 0.221 | 0.95 | [0.75, 1.21] | 0.365 | 1.03 | [0.78, 1.34] | 0.390 | 1.13 | [0.81, 1.57] | 0.307 | 1.21 | [0.82, 1.78] | 0.251 | 1.25 | [0.82, 1.90] | 0.232 |
| **Head: Alumina, Liner: non-HCLPE** | 1.03 | [0.69, 1.54] | 0.395 | 1.12 | [0.76, 1.63] | 0.337 | 1.21 | [0.81, 1.80] | 0.257 | 1.33 | [0.83, 2.15] | 0.200 | 1.43 | [0.82, 2.50] | 0.181 | 1.49 | [0.81, 2.74] | 0.175 |
| **Head: Delta Ceramic, Liner: Delta Ceramic** | 0.82 | [0.72, 0.94] | 0.006 | 0.82 | [0.71, 0.94] | 0.009 | 0.81 | [0.69, 0.95] | 0.014 | 0.80 | [0.66, 0.97] | 0.030 | 0.80 | [0.64, 0.99] | 0.053 | 0.79 | [0.63, 1.00] | 0.054 |
| **Head: Delta Ceramic, Liner: HCLPE** | 0.90 | [0.79, 1.02] | 0.108 | 0.90 | [0.79, 1.03] | 0.119 | 0.90 | [0.77, 1.06] | 0.173 | 0.91 | [0.75, 1.10] | 0.250 | 0.91 | [0.73, 1.14] | 0.283 | 0.91 | [0.72, 1.16] | 0.295 |
| **Head: Delta Ceramic, Liner: non-HCLPE** | 0.76 | [0.58, 0.98] | 0.049 | 0.98 | [0.79, 1.22] | 0.392 | 1.25 | [1.02, 1.55] | 0.045 | 1.74 | [1.36, 2.21] | <0.001 | 2.20 | [1.64, 2.95] | <0.001 | 2.51 | [1.81, 3.47] | <0.001 |
| **Head: Oxidised Zirconium, Liner: HCLPE** | 0.96 | [0.75, 1.22] | 0.378 | 1.07 | [0.83, 1.39] | 0.350 | 1.19 | [0.88, 1.61] | 0.211 | 1.37 | [0.94, 1.99] | 0.103 | 1.51 | [0.97, 2.35] | 0.075 | 1.59 | [0.99, 2.58] | 0.066 |
| **Head: Cobalt Chrome, Liner: non-HCLPE** | 1.06 | [0.93, 1.22] | 0.280 | 1.18 | [1.04, 1.35] | 0.018 | 1.31 | [1.13, 1.51] | <0.001 | 1.49 | [1.26, 1.78] | <0.001 | 1.64 | [1.34, 2.01] | <0.001 | 1.73 | [1.39, 2.14] | <0.001 |
| **Head: Stainless Steel, Liner: HCLPE** | 1.04 | [0.86, 1.26] | 0.368 | 1.10 | [0.90, 1.34] | 0.257 | 1.15 | [0.92, 1.45] | 0.193 | 1.23 | [0.94, 1.62] | 0.131 | 1.29 | [0.94, 1.76] | 0.112 | 1.32 | [0.94, 1.84] | 0.107 |
| **Head: Stainless Steel, Liner: non-HCLPE** | 1.14 | [0.93, 1.40] | 0.181 | 1.29 | [1.05, 1.57] | 0.018 | 1.44 | [1.17, 1.77] | 0.001 | 1.67 | [1.32, 2.13] | <0.001 | 1.86 | [1.42, 2.45] | <0.001 | 1.98 | [1.47, 2.66] | <0.001 |
| **Head: Delta Ceramic, Pre-assembled implant** | 0.28 | [0.13, 0.63] | 0.003 | 0.25 | [0.10, 0.61] | 0.004 | 0.21 | [0.07, 0.62] | 0.008 | 0.18 | [0.05, 0.65] | 0.013 | 0.16 | [0.04, 0.68] | 0.016 | 0.15 | [0.03, 0.69] | 0.024 |
| **Head: Cobalt Chrome or Stainless Steel, Pre-assembled implant** | 1.42 | [0.87, 2.33] | 0.151 | 1.39 | [0.82, 2.37] | 0.190 | 1.36 | [0.75, 2.49] | 0.241 | 1.33 | [0.65, 2.73] | 0.295 | 1.31 | [0.58, 2.94] | 0.323 | 1.3 | [0.55, 3.07] | 0.334 |

Flexible parametric survival model adjusted for year of primary surgery, patient gender, age, Body Mass Index,  American Society of Anesthesiologists grade, implant fixation, shell composition, stem composition and head size. HCLPE: Highly CrossLinked PolyethylenE.

Wald test

**Table X:** Modular acetabular component- Revision for pain Hazard Ratio (HR) and 95% Confidence Interval (CI) by time-point from primary procedure-Reference: Implant with cobalt chrome head and highly cross-linked polyethylene liner

|  | **6 months** | |  | **1 year** | |  | **2 years** | |  | **5 years** | |  | **10 years** | |  | **15 years** | |  |
| --- | --- | --- | --- | --- | --- | --- | --- | --- | --- | --- | --- | --- | --- | --- | --- | --- | --- | --- |
|  | **HR** | **95% CI** | **P-value** | **HR** | **95% CI** | **P-value** | **HR** | **95% CI** | **P-value** | **HR** | **95% CI** | **P-value** | **HR** | **95% CI** | **P-value** | **HR** | **95% CI** | **P-value** |
| **Head: Alumina, Liner: Alumina** | 1.46 | [1.03, 2.07] | 0.042 | 1.73 | [1.31, 2.30] | <0.001 | 2.05 | [1.61, 2.60] | <0.001 | 2.97 | [2.25, 3.93] | <0.001 | 4.17 | [2.70, 6.43] | <0.001 | 4.81 | [2.89, 7.98] | <0.001 |
| **Head: Alumina, Liner: HCLPE** | 1.06 | [0.67, 1.66] | 0.386 | 1.13 | [0.78, 1.63] | 0.323 | 1.20 | [0.86, 1.68] | 0.226 | 1.40 | [0.88, 2.24] | 0.147 | 1.63 | [0.79, 3.38] | 0.167 | 1.73 | [0.74, 4.04] | 0.179 |
| **Head: Alumina, Liner: non-HCLPE** | 1.88 | [0.85, 4.13] | 0.117 | 1.89 | [0.98, 3.62] | 0.064 | 1.90 | [1.09, 3.32] | 0.031 | 1.93 | [1.01, 3.71] | 0.056 | 1.96 | [0.68, 5.66] | 0.184 | 1.98 | [0.57, 6.88] | 0.224 |
| **Head: Delta Ceramic, Liner: Delta Ceramic** | 0.98 | [0.78, 1.23] | 0.393 | 1.02 | [0.84, 1.24] | 0.391 | 1.06 | [0.88, 1.27] | 0.329 | 1.17 | [0.91, 1.50] | 0.187 | 1.28 | [0.87, 1.89] | 0.183 | 1.33 | [0.85, 2.09] | 0.184 |
| **Head: Delta Ceramic, Liner: HCLPE** | 0.47 | [0.35, 0.62] | <0.001 | 0.47 | [0.36, 0.60] | <0.001 | 0.46 | [0.36, 0.60] | <0.001 | 0.46 | [0.30, 0.69] | <0.001 | 0.45 | [0.24, 0.87] | 0.021 | 0.45 | [0.21, 0.95] | 0.046 |
| **Head: Delta Ceramic, Liner: non-HCLPE** | 1.33 | [0.86, 2.07] | 0.177 | 1.31 | [0.90, 1.91] | 0.148 | 1.30 | [0.92, 1.82] | 0.128 | 1.25 | [0.82, 1.91] | 0.234 | 1.21 | [0.62, 2.36] | 0.341 | 1.19 | [0.54, 2.61] | 0.363 |
| **Head: Oxidised Zirconium, Liner: HCLPE** | 0.53 | [0.31, 0.90] | 0.026 | 0.49 | [0.30, 0.81] | 0.008 | 0.46 | [0.27, 0.78] | 0.007 | 0.37 | [0.15, 0.94] | 0.042 | 0.30 | [0.06, 1.48] | 0.135 | 0.27 | [0.04, 1.81] | 0.161 |
| **Head: Cobalt Chrome, Liner: non-HCLPE** | 1.89 | [1.42, 2.51] | <0.001 | 2.07 | [1.63, 2.63] | <0.001 | 2.26 | [1.83, 2.81] | <0.001 | 2.80 | [2.14, 3.66] | <0.001 | 3.42 | [2.26, 5.17] | <0.001 | 3.71 | [2.29, 6.01] | <0.001 |
| **Head: Stainless Steel, Liner: HCLPE** | 0.86 | [0.52, 1.42] | 0.336 | 0.85 | [0.55, 1.34] | 0.309 | 0.85 | [0.54, 1.32] | 0.309 | 0.83 | [0.43, 1.60] | 0.342 | 0.82 | [0.30, 2.25] | 0.370 | 0.81 | [0.25, 2.61] | 0.375 |
| **Head: Stainless Steel, Liner: non-HCLPE** | 2.12 | [1.32, 3.42] | 0.003 | 2.39 | [1.60, 3.55] | <0.001 | 2.69 | [1.90, 3.80] | <0.001 | 3.51 | [2.40, 5.15] | <0.001 | 4.51 | [2.60, 7.84] | <0.001 | 5.01 | [2.64, 9.48] | <0.001 |
| **Head: Delta Ceramic, Pre-assembled implant** | 1.04 | [0.50, 2.14] | 0.397 | 0.99 | [0.50, 1.92] | 0.399 | 0.93 | [0.48, 1.80] | 0.390 | 0.81 | [0.35, 1.88] | 0.354 | 0.69 | [0.19, 2.54] | 0.341 | 0.65 | [0.14, 2.93] | 0.342 |
| **Head: Cobalt Chrome or Stainless Steel, Pre-assembled implant** | 1.52 | [0.38, 6.15] | 0.335 | 1.57 | [0.49, 5.02] | 0.299 | 1.63 | [0.56, 4.73] | 0.267 | 1.77 | [0.39, 7.98] | 0.303 | 1.92 | [0.18, 20.10] | 0.344 | 1.99 | [0.13, 30.31] | 0.353 |

Flexible parametric survival model adjusted for year of primary surgery, patient gender, age, Body Mass Index,  American Society of Anesthesiologists grade, implant fixation, shell composition, stem composition and head size. HCLPE: Highly CrossLinked PolyethylenE.

Wald test

**Table Y:** Modular acetabular component- Revision for infection Hazard Ratio (HR) and 95% Confidence Interval (CI) by time-point from primary procedure-Reference: Implant with cobalt chrome head and highly cross-linked polyethylene liner

|  | **6 months** | |  | **1 year** | |  | **2 years** | |  | **5 years** | |  | **10 years** | |  | **15 years** | |  |
| --- | --- | --- | --- | --- | --- | --- | --- | --- | --- | --- | --- | --- | --- | --- | --- | --- | --- | --- |
|  | **HR** | **95% CI** | **P-value** | **HR** | **95% CI** | **P-value** | **HR** | **95% CI** | **P-value** | **HR** | **95% CI** | **P-value** | **HR** | **95% CI** | **P-value** | **HR** | **95% CI** | **P-value** |
| **Head: Alumina, Liner: Alumina** | 0.70 | [0.56, 0.87] | 0.003 | 0.71 | [0.57, 0.88] | 0.003 | 0.76 | [0.60, 0.96] | 0.029 | 0.81 | [0.62, 1.05] | 0.117 | 0.85 | [0.63, 1.15] | 0.228 | 0.88 | [0.63, 1.22] | 0.299 |
| **Head: Alumina, Liner: HCLPE** | 0.80 | [0.62, 1.03] | 0.090 | 0.80 | [0.63, 1.03] | 0.082 | 0.77 | [0.58, 1.03] | 0.081 | 0.75 | [0.55, 1.04] | 0.083 | 0.73 | [0.52, 1.05] | 0.085 | 0.72 | [0.50, 1.06] | 0.092 |
| **Head: Alumina, Liner: non-HCLPE** | 0.85 | [0.46, 1.57] | 0.349 | 0.92 | [0.54, 1.58] | 0.381 | 1.12 | [0.69, 1.82] | 0.359 | 1.38 | [0.80, 2.35] | 0.201 | 1.61 | [0.85, 3.06] | 0.138 | 1.78 | [0.86, 3.66] | 0.118 |
| **Head: Delta Ceramic, Liner: Delta Ceramic** | 0.76 | [0.66, 0.88] | <0.001 | 0.77 | [0.67, 0.89] | <0.001 | 0.82 | [0.70, 0.96] | 0.019 | 0.86 | [0.72, 1.03] | 0.102 | 0.90 | [0.73, 1.11] | 0.245 | 0.93 | [0.74, 1.15] | 0.324 |
| **Head: Delta Ceramic, Liner: HCLPE** | 0.73 | [0.62, 0.85] | <0.001 | 0.73 | [0.63, 0.85] | <0.001 | 0.69 | [0.58, 0.83] | <0.001 | 0.67 | [0.55, 0.82] | <0.001 | 0.65 | [0.52, 0.81] | <0.001 | 0.63 | [0.50, 0.80] | <0.001 |
| **Head: Delta Ceramic, Liner: non-HCLPE** | 0.67 | [0.47, 0.95] | 0.033 | 0.70 | [0.50, 0.98] | 0.046 | 0.81 | [0.58, 1.12] | 0.181 | 0.93 | [0.64, 1.37] | 0.372 | 1.04 | [0.67, 1.63] | 0.393 | 1.12 | [0.68, 1.83] | 0.361 |
| **Head: Oxidised Zirconium, Liner: HCLPE** | 0.69 | [0.50, 0.96] | 0.033 | 0.69 | [0.50, 0.95] | 0.031 | 0.67 | [0.46, 0.98] | 0.046 | 0.66 | [0.43, 1.02] | 0.067 | 0.65 | [0.41, 1.05] | 0.080 | 0.65 | [0.39, 1.07] | 0.098 |
| **Head: Cobalt Chrome, Liner: non-HCLPE** | 1.07 | [0.89, 1.29] | 0.309 | 1.10 | [0.91, 1.32] | 0.241 | 1.21 | [0.99, 1.46] | 0.063 | 1.32 | [1.05, 1.66] | 0.024 | 1.42 | [1.09, 1.84] | 0.013 | 1.49 | [1.12, 1.97] | 0.009 |
| **Head: Stainless Steel, Liner: HCLPE** | 0.91 | [0.72, 1.14] | 0.289 | 0.92 | [0.73, 1.14] | 0.305 | 0.86 | [0.67, 1.11] | 0.201 | 0.82 | [0.62, 1.09] | 0.154 | 0.80 | [0.59, 1.08] | 0.140 | 0.78 | [0.56, 1.07] | 0.129 |
| **Head: Stainless Steel, Liner: non-HCLPE** | 1.17 | [0.89, 1.53] | 0.209 | 1.26 | [0.98, 1.61] | 0.075 | 1.51 | [1.18, 1.92] | 0.002 | 1.82 | [1.38, 2.40] | <0.001 | 2.11 | [1.53, 2.90] | <0.001 | 2.31 | [1.63, 3.29] | <0.001 |
| **Head: Delta Ceramic, Pre-assembled implant** | 0.28 | [0.13, 0.59] | 0.002 | 0.29 | [0.13, 0.61] | 0.003 | 0.32 | [0.14, 0.72] | 0.010 | 0.35 | [0.14, 0.91] | 0.036 | 0.38 | [0.13, 1.12] | 0.085 | 0.40 | [0.12, 1.30] | 0.128 |
| **Head: Cobalt Chrome or Stainless Steel, Pre-assembled implant** | 0.64 | [0.24, 1.71] | 0.268 | 0.65 | [0.25, 1.65] | 0.267 | 0.6 | [0.21, 1.71] | 0.253 | 0.57 | [0.18, 1.77] | 0.251 | 0.54 | [0.16, 1.83] | 0.244 | 0.53 | [0.15, 1.89] | 0.246 |

Flexible parametric survival model adjusted for year of primary surgery, patient gender, age, Body Mass Index,  American Society of Anesthesiologists grade, implant fixation, shell composition, stem composition and head size. HCLPE: Highly CrossLinked PolyethylenE.

Wald test

**Table Z:** Modular acetabular component- Revision for any other reason(s) Hazard Ratio (HR) and 95% Confidence Interval (CI) by time-point from primary procedure-Reference: Implant with cobalt chrome head and highly cross-linked polyethylene liner

|  | **6 months** | |  | **1 year** | |  | **2 years** | |  | **5 years** | |  | **10 years** | |  | **15 years** | |  |
| --- | --- | --- | --- | --- | --- | --- | --- | --- | --- | --- | --- | --- | --- | --- | --- | --- | --- | --- |
|  | **HR** | **95% CI** | **P-value** | **HR** | **95% CI** | **P-value** | **HR** | **95% CI** | **P-value** | **HR** | **95% CI** | **P-value** | **HR** | **95% CI** | **P-value** | **HR** | **95% CI** | **P-value** |
| **Head: Alumina, Liner: Alumina** | 1.36 | [1.10, 1.67] | 0.006 | 1.35 | [1.10, 1.64] | 0.005 | 1.46 | [1.22, 1.76] | <0.001 | 1.65 | [1.35, 2.01] | <0.001 | 1.67 | [1.36, 2.05] | <0.001 | 1.74 | [1.39, 2.17] | <0.001 |
| **Head: Alumina, Liner: HCLPE** | 0.87 | [0.66, 1.14] | 0.242 | 0.87 | [0.66, 1.14] | 0.242 | 0.86 | [0.65, 1.14] | 0.229 | 0.85 | [0.62, 1.18] | 0.244 | 0.85 | [0.62, 1.17] | 0.24 | 0.85 | [0.61, 1.19] | 0.25 |
| **Head: Alumina, Liner: non-HCLPE** | 0.45 | [0.13, 1.57] | 0.181 | 0.55 | [0.21, 1.47] | 0.193 | 0.84 | [0.42, 1.65] | 0.352 | 1.49 | [1.00, 2.22] | 0.058 | 1.84 | [1.20, 2.81] | 0.01 | 2.26 | [1.33, 3.85] | 0.00 |
| **Head: Delta Ceramic, Liner: Delta Ceramic** | 1.10 | [0.96, 1.26] | 0.155 | 1.10 | [0.96, 1.26] | 0.155 | 1.10 | [0.96, 1.26] | 0.155 | 1.10 | [0.94, 1.29] | 0.199 | 1.10 | [0.94, 1.29] | 0.20 | 1.10 | [0.93, 1.30] | 0.21 |
| **Head: Delta Ceramic, Liner: HCLPE** | 0.61 | [0.51, 0.73] | <0.001 | 0.62 | [0.52, 0.73] | <0.001 | 0.60 | [0.50, 0.73] | <0.001 | 0.59 | [0.47, 0.73] | <0.001 | 0.59 | [0.47, 0.73] | <0.001 | 0.58 | [0.46, 0.73] | <0.001 |
| **Head: Delta Ceramic, Liner: non-HCLPE** | 0.93 | [0.71, 1.22] | 0.347 | 0.99 | [0.77, 1.27] | 0.398 | 0.94 | [0.73, 1.21] | 0.356 | 0.87 | [0.65, 1.15] | 0.252 | 0.88 | [0.67, 1.15] | 0.26 | 0.85 | [0.64, 1.14] | 0.22 |
| **Head: Oxidised Zirconium, Liner: HCLPE** | 0.53 | [0.35, 0.81] | 0.005 | 0.57 | [0.39, 0.81] | 0.004 | 0.54 | [0.36, 0.79] | 0.004 | 0.50 | [0.32, 0.77] | 0.003 | 0.50 | [0.33, 0.77] | 0.00 | 0.49 | [0.31, 0.76] | 0.00 |
| **Head: Cobalt Chrome, Liner: non-HCLPE** | 1.46 | [1.23, 1.74] | <0.001 | 1.45 | [1.22, 1.72] | <0.001 | 1.54 | [1.31, 1.81] | <0.001 | 1.67 | [1.39, 2.00] | <0.001 | 1.68 | [1.39, 2.02] | <0.001 | 1.73 | [1.41, 2.11] | <0.001 |
| **Head: Stainless Steel, Liner: HCLPE** | 0.77 | [0.54, 1.10] | 0.141 | 0.85 | [0.63, 1.15] | 0.228 | 0.79 | [0.58, 1.08] | 0.132 | 0.71 | [0.50, 1.01] | 0.064 | 0.73 | [0.52, 1.01] | 0.07 | 0.70 | [0.50, 0.99] | 0.05 |
| **Head: Stainless Steel, Liner: non-HCLPE** | 1.51 | [1.13, 2.03] | 0.009 | 1.51 | [1.14, 2.00] | 0.006 | 1.66 | [1.30, 2.12] | <0.001 | 1.89 | [1.46, 2.45] | <0.001 | 1.93 | [1.47, 2.52] | <0.001 | 2.01 | [1.51, 2.70] | <0.001 |
| **Head: Delta Ceramic, Pre-assembled implant** | 0.80 | [0.43, 1.50] | 0.312 | 0.83 | [0.46, 1.50] | 0.330 | 0.80 | [0.43, 1.47] | 0.310 | 0.76 | [0.38, 1.49] | 0.293 | 0.76 | [0.39, 1.47] | 0.29 | 0.75 | [0.37, 1.49] | 0.29 |
| **Head: Cobalt Chrome or Stainless Steel, Pre-assembled implant** | 1.26 | [0.49, 3.20] | 0.355 | 1.29 | [0.54, 3.09] | 0.339 | 1.25 | [0.50, 3.13] | 0.356 | 1.19 | [0.41, 3.44] | 0.379 | 1.19 | [0.42, 3.36] | 0.378 | 1.17 | [0.39, 3.51] | 0.384 |

Flexible parametric survival model adjusted for year of primary surgery, patient gender, age, Body Mass Index,  American Society of Anesthesiologists grade, implant fixation, shell composition, stem composition and head size. HCLPE: Highly CrossLinked PolyethylenE.

Wald test

## Supplementary 3: Study flow diagram


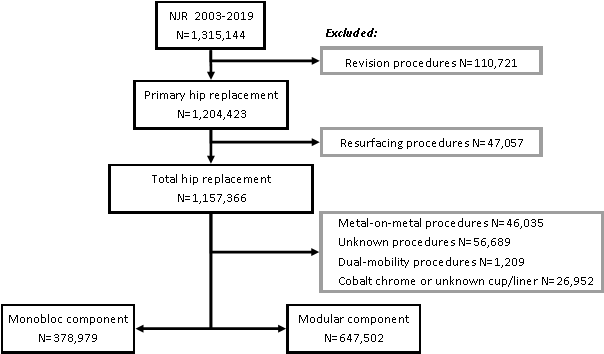


**Figure A:** PRISMA flow diagram

## Supplementary 4: Aseptic loosening

## Monobloc acetabular component


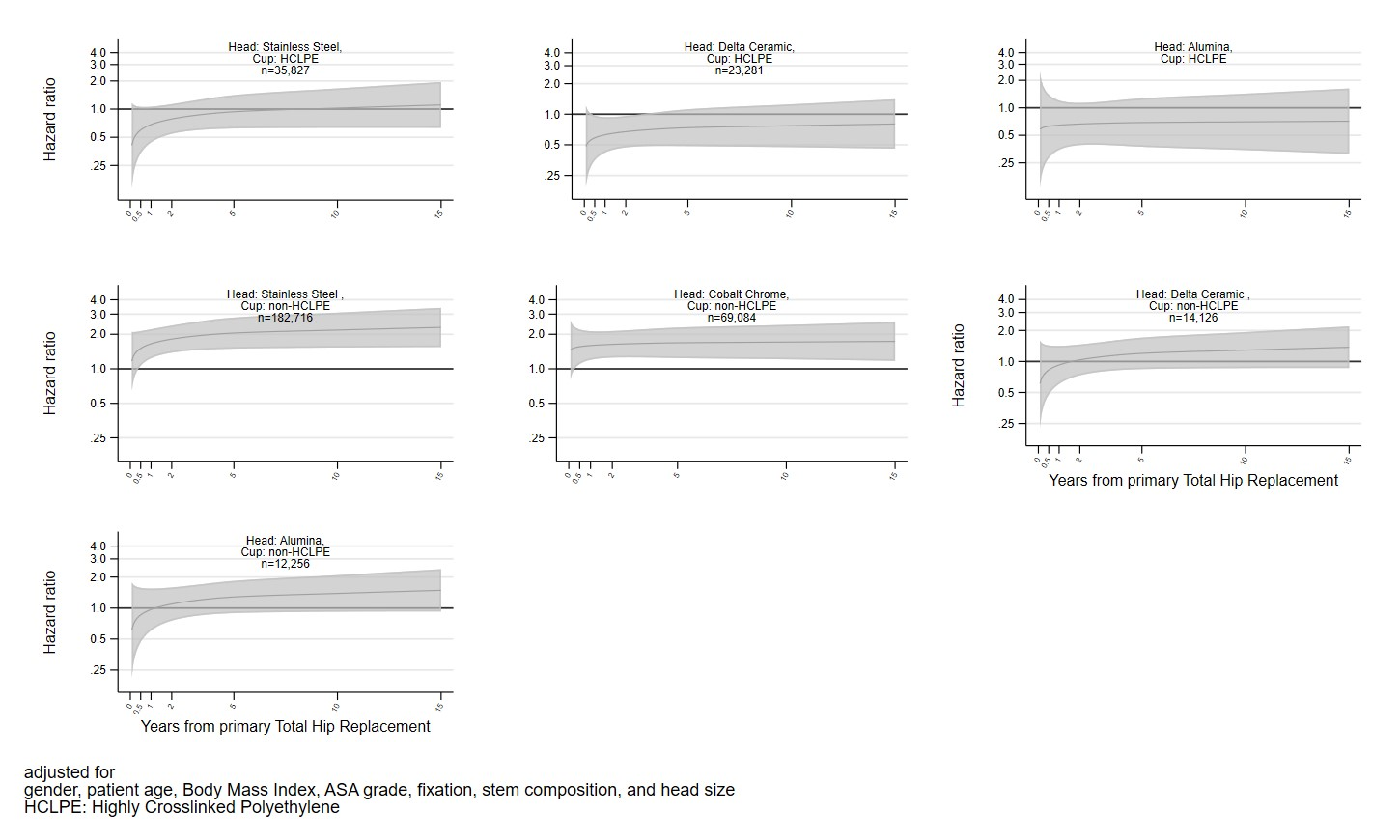


**Figure B:** Risk of revision for aseptic loosening by head and cup types (Reference: Cobalt chrome head with highly crosslinked polyethylene cup). Flexible parametric survival model adjusted for year of primary surgery, patient gender, age, Body Mass Index,  American Society of Anesthesiologists grade, implant fixation, stem composition and head size. HCLPE: Highly CrossLinked PolyethylenE.

## Modular acetabular component


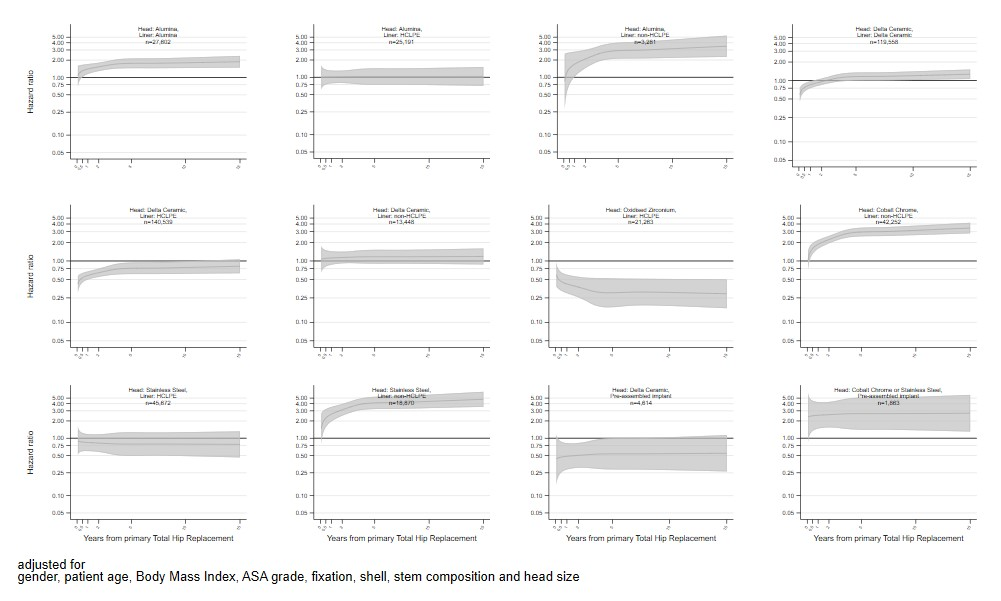


**Figure C:** Risk of revision for aseptic loosening by head and liner types (Reference: Cobalt chrome head with highly crosslinked polyethylene liner). Flexible parametric survival model adjusted for year of primary surgery, patient gender, age, Body Mass Index,  American Society of Anesthesiologists grade, implant fixation, shell composition, stem composition and head size. HCLPE: Highly CrossLinked PolyethylenE.

### Supplementary 5: Periprosthetic fracture

## Monobloc acetabular component


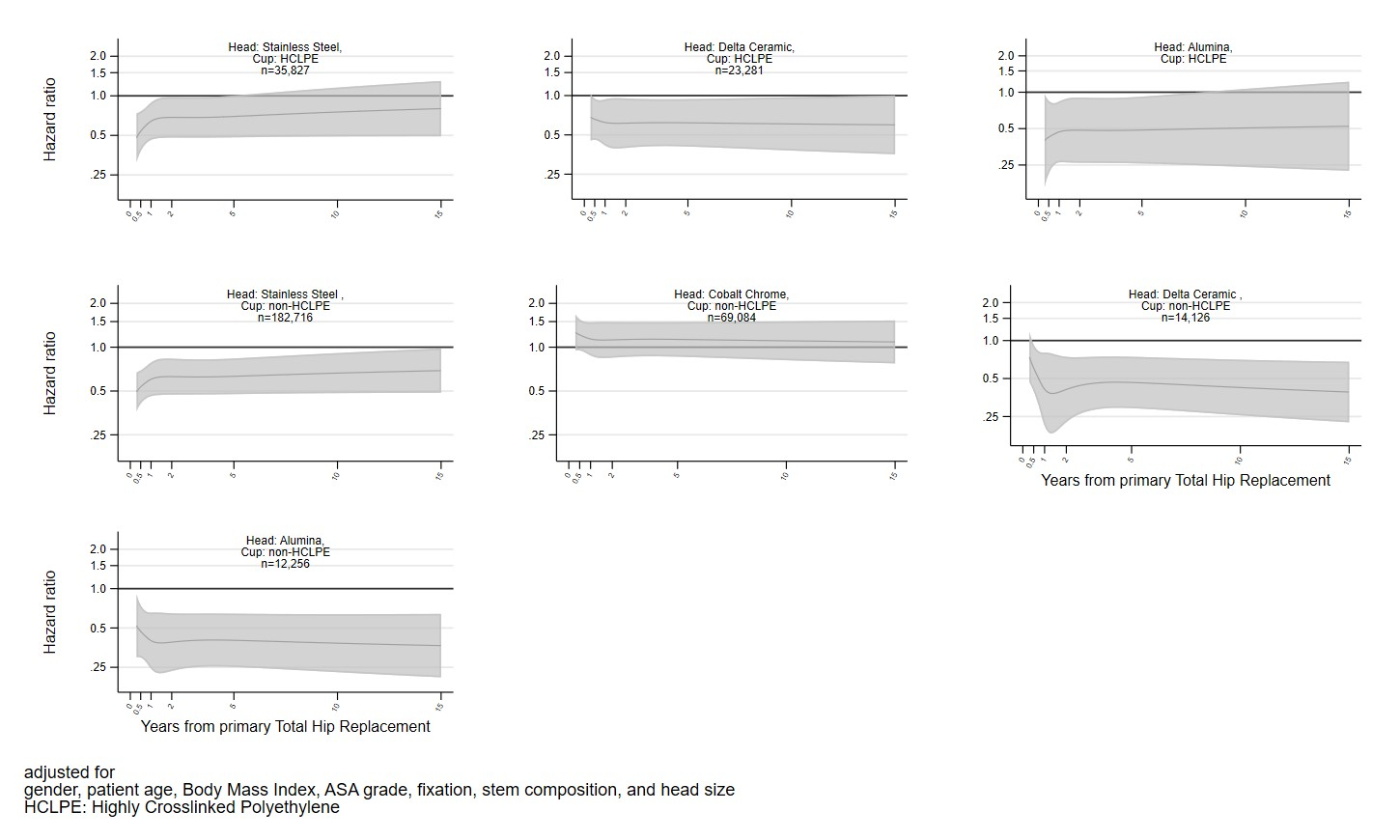


**Figure D:** Risk of revision for periprosthetic fracture by head and cup types (Reference: Cobalt chrome head with highly crosslinked polyethylene cup). Flexible parametric survival model adjusted for year of primary surgery, patient gender, age, Body Mass Index,  American Society of Anesthesiologists grade, implant fixation, stem composition and head size. HCLPE: Highly CrossLinked PolyethylenE.

## Modular acetabular component


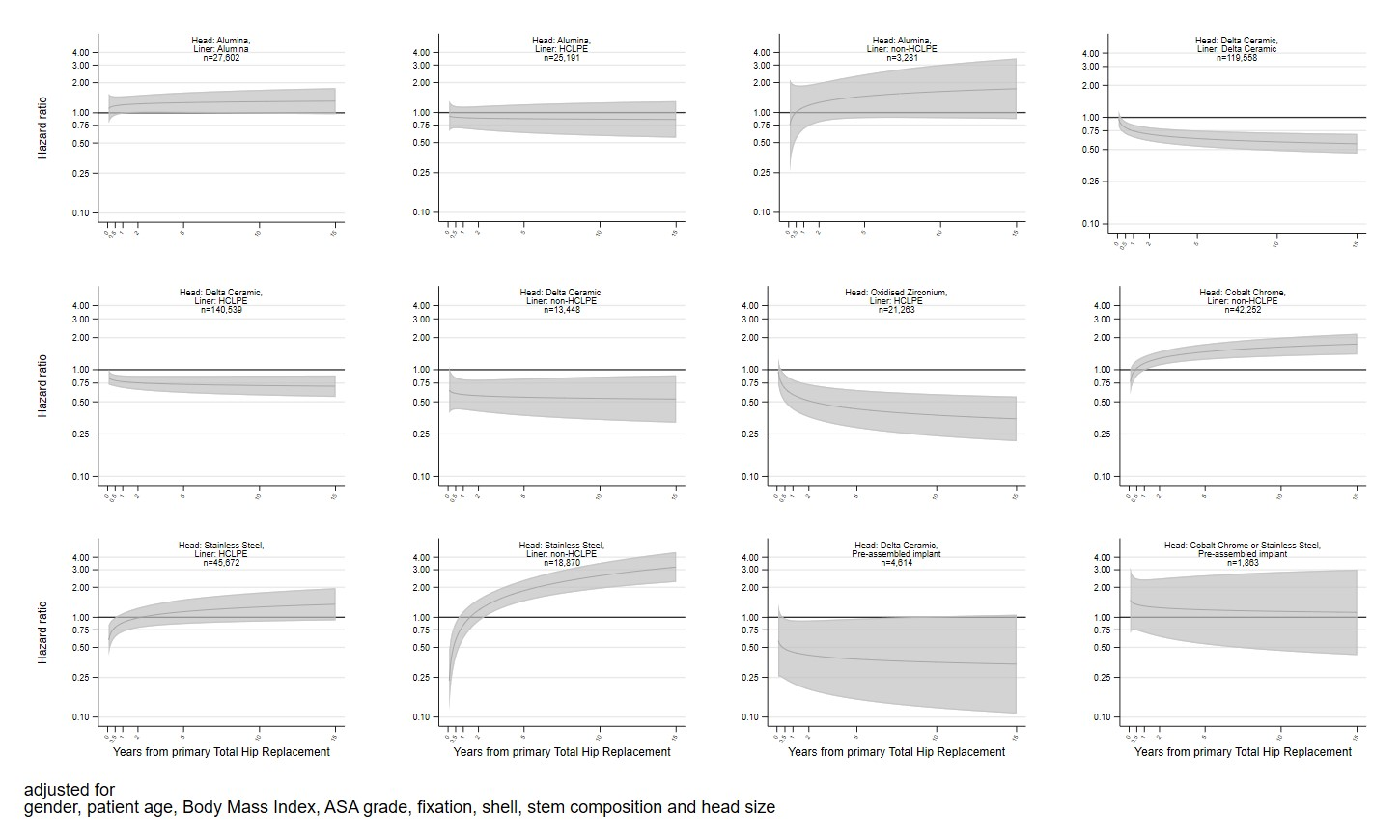
**Figure E:** Risk of revision for periprosthetic fracture by head and liner types (Reference: Cobalt chrome head with highly crosslinked polyethylene liner). Flexible parametric survival model adjusted for year of primary surgery, patient gender, age, Body Mass Index,  American Society of Anesthesiologists grade, implant fixation, shell composition, stem composition and head size. HCLPE: Highly CrossLinked PolyethylenE.

### Supplementary 6: Implant wear

## Monobloc acetabular component


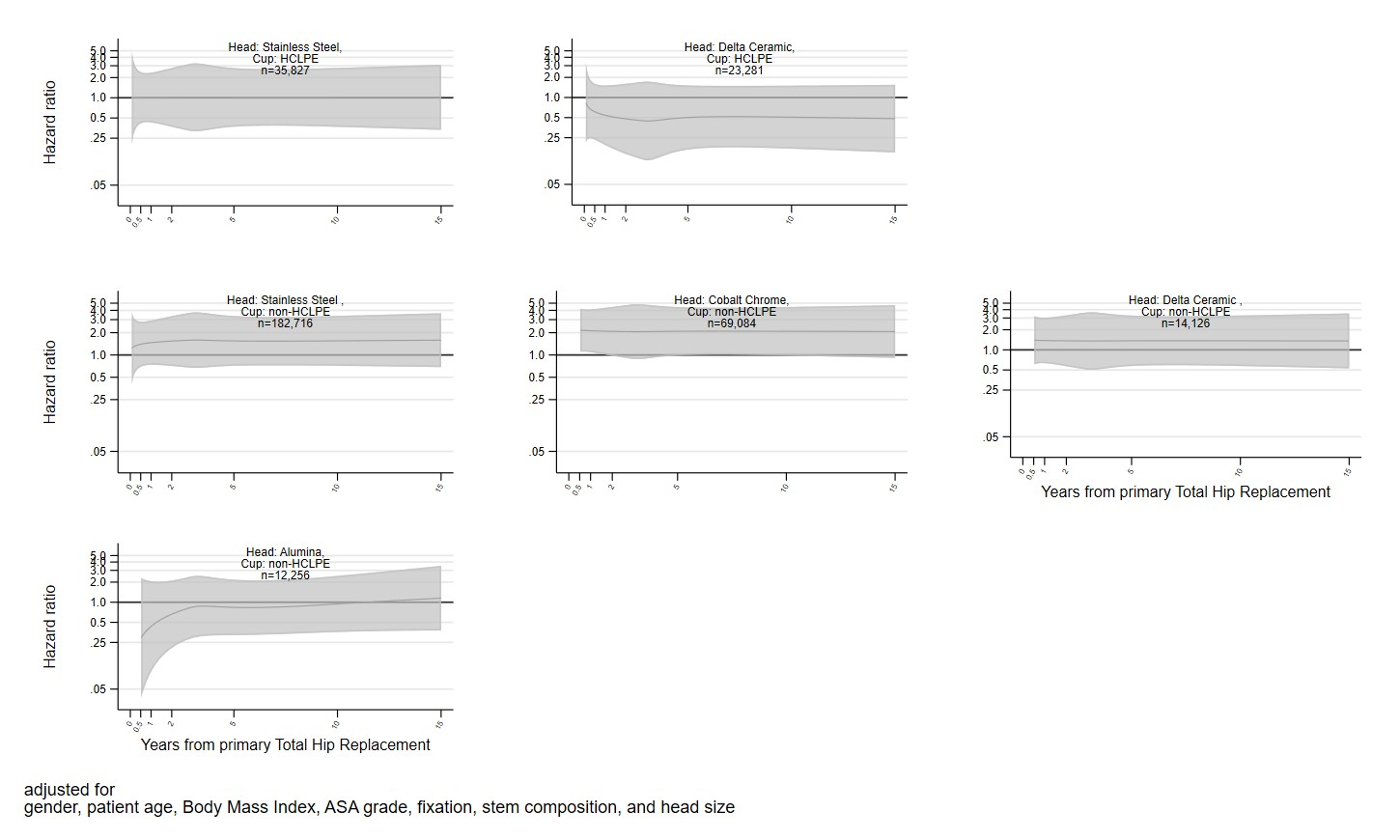


**Figure F:** Risk of revision for implant wear by head and cup types (Reference: Cobalt chrome head with highly crosslinked polyethylene cup). Flexible parametric survival model adjusted for year of primary surgery, patient gender, age, Body Mass Index,  American Society of Anesthesiologists grade, implant fixation, stem composition and head size. HCLPE: Highly CrossLinked PolyethylenE.

## Modular acetabular component


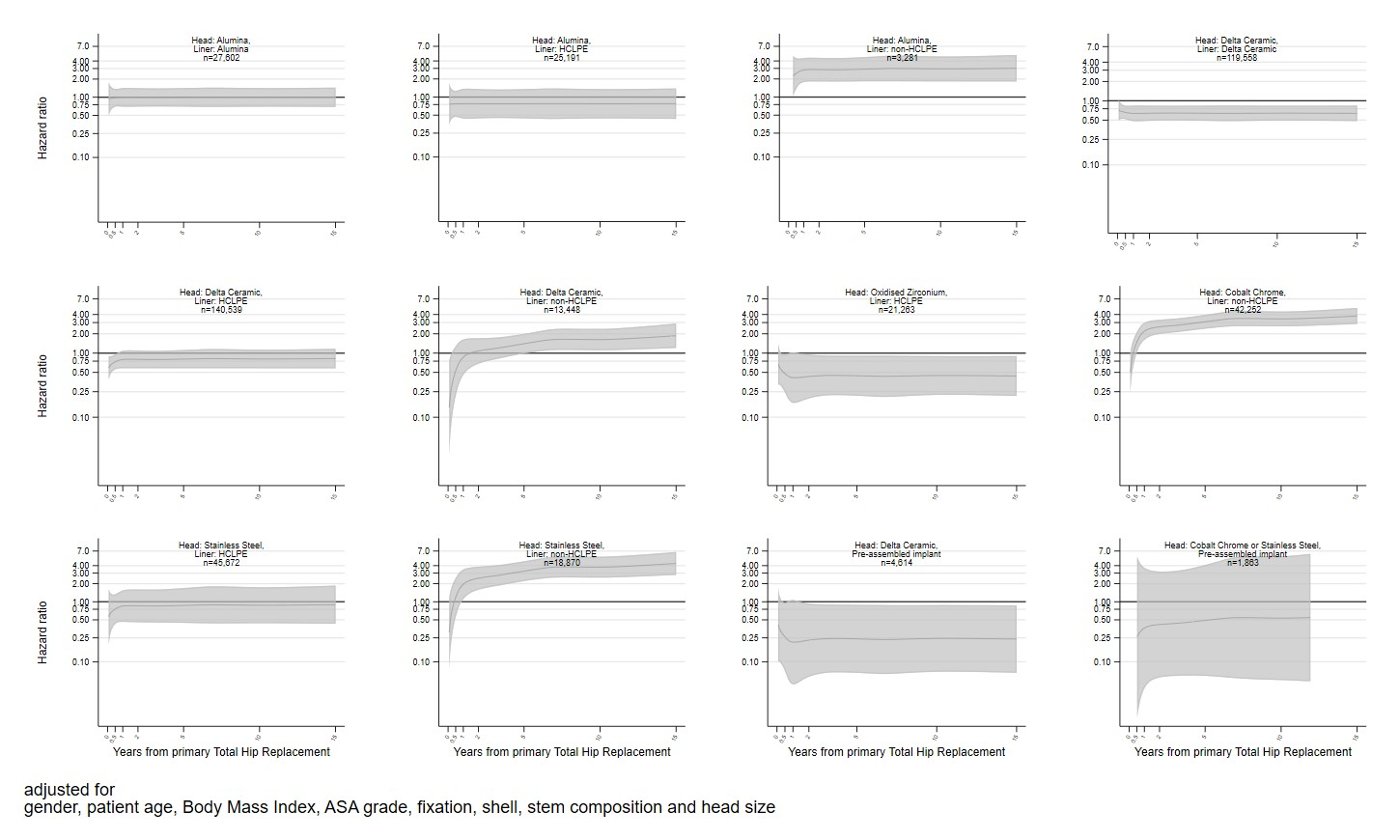
**Figure G:** Risk of revision for implant wear by head and liner types (Reference: Cobalt chrome head with highly crosslinked polyethylene liner). Flexible parametric survival model adjusted for year of primary surgery, patient gender, age, Body Mass Index,  American Society of Anesthesiologists grade, implant fixation, shell composition, stem composition and head size. HCLPE: Highly CrossLinked PolyethylenE.

### Supplementary 7: Malalignment

## Monobloc acetabular component


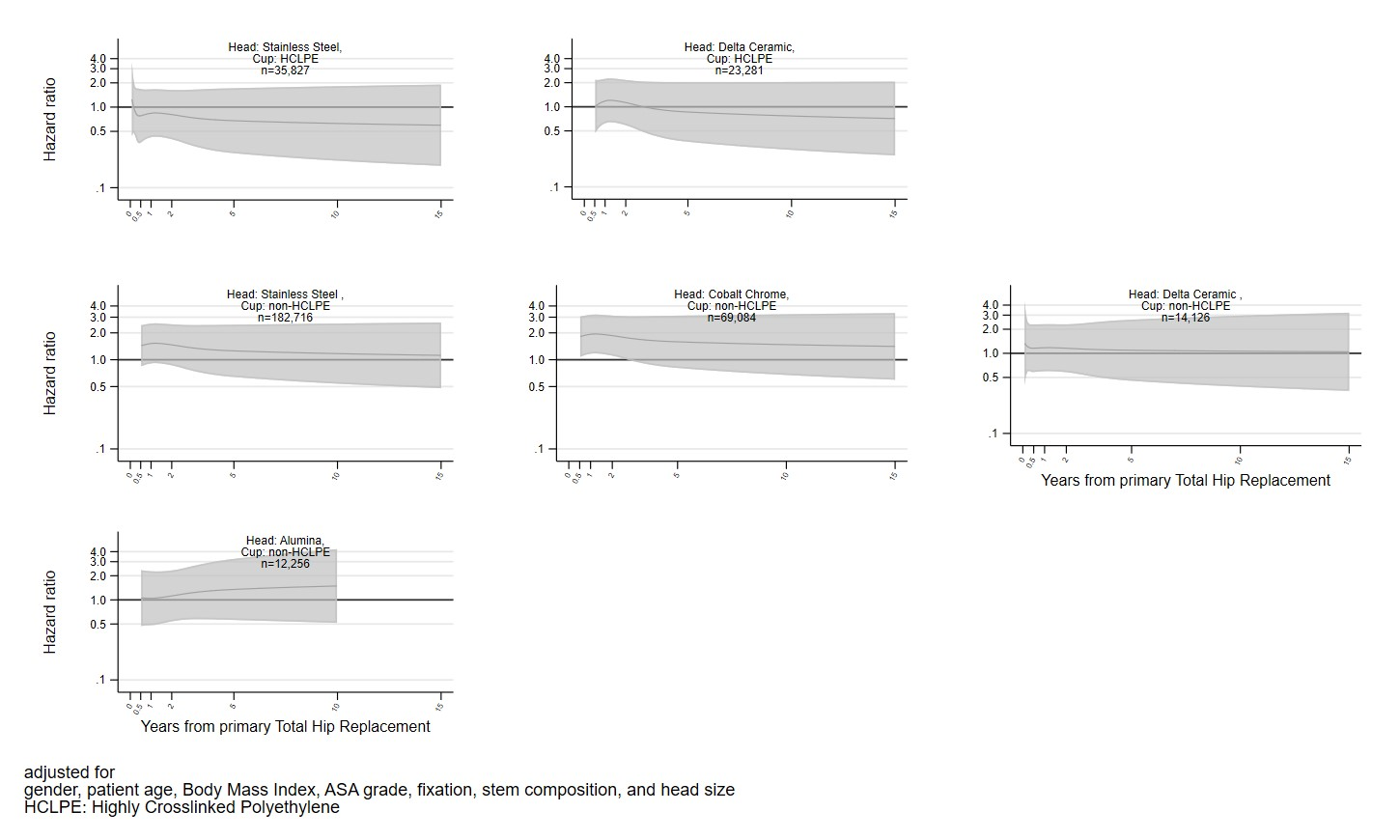
**Figure H:** Risk of revision for malignment by head and cup types (Reference: Cobalt chrome head with highly crosslinked polyethylene cup). Flexible parametric survival model adjusted for year of primary surgery, patient gender, age, Body Mass Index,  American Society of Anesthesiologists grade, implant fixation, stem composition and head size. HCLPE: Highly CrossLinked PolyethylenE.

## Modular acetabular component
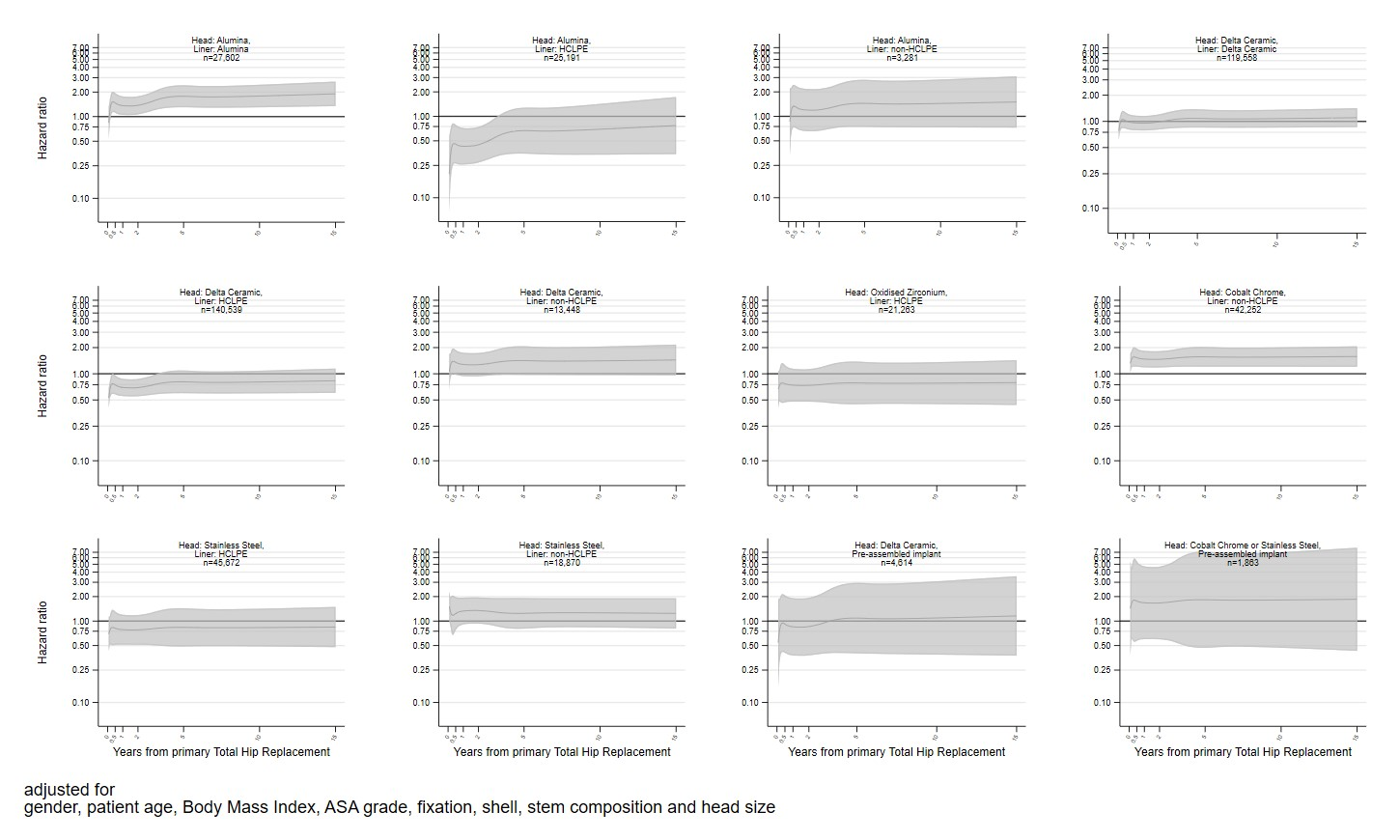


**Figure I:** Risk of revision for malignment by head and liner types (Reference: Cobalt chrome head with highly crosslinked polyethylene liner). Flexible parametric survival model adjusted for year of primary surgery, patient gender, age, Body Mass Index,  American Society of Anesthesiologists grade, implant fixation, shell composition, stem composition and head size. HCLPE: Highly CrossLinked PolyethylenE.

### Supplementary 8: Dislocation or subluxation

## Monobloc acetabular component


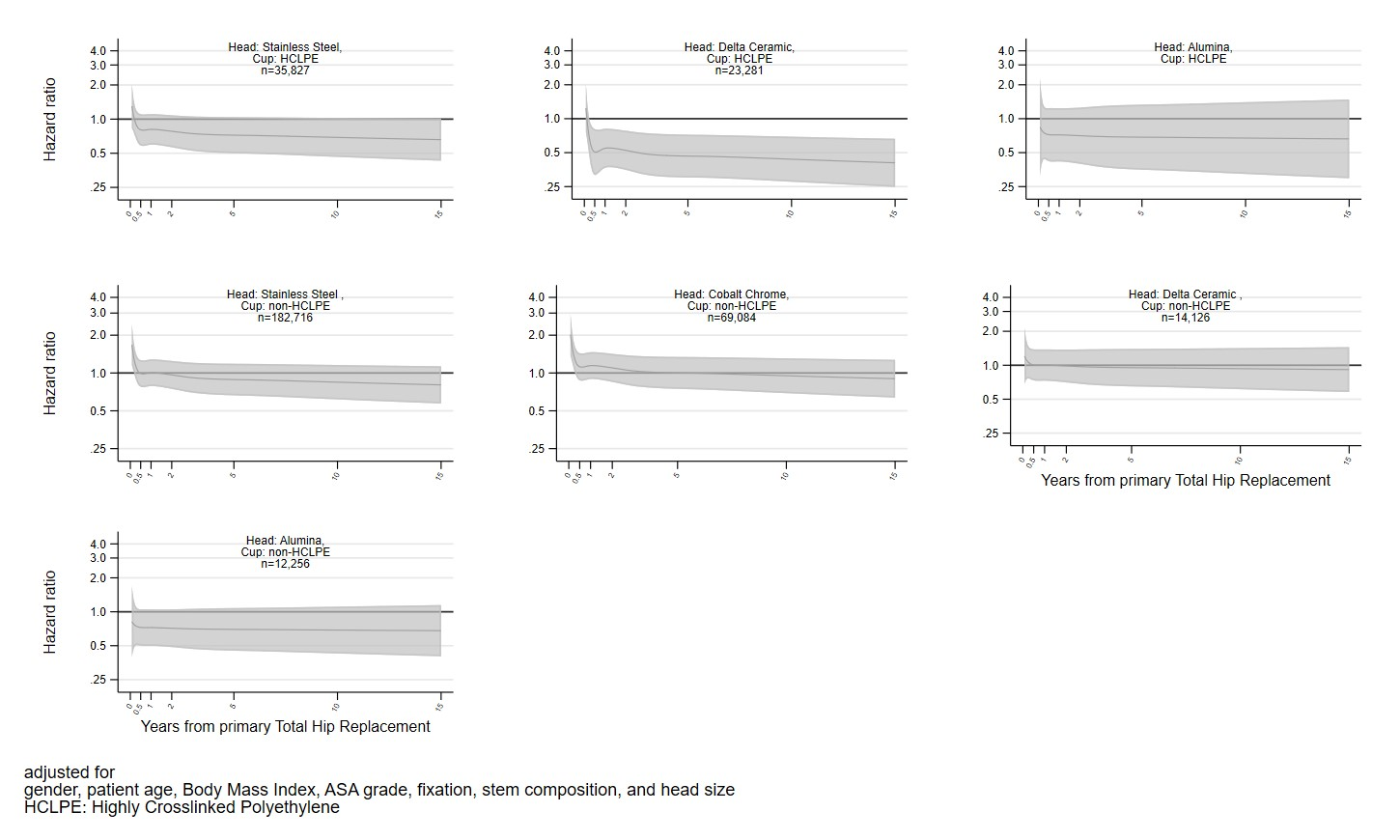


**Figure J:** Risk of revision for dislocation or subluxation by head and cup types (Reference: Cobalt chrome head with highly crosslinked polyethylene cup). Flexible parametric survival model adjusted for year of primary surgery, patient gender, age, Body Mass Index,  American Society of Anesthesiologists grade, implant fixation, stem composition and head size. HCLPE: Highly CrossLinked PolyethylenE.

## Modular acetabular component


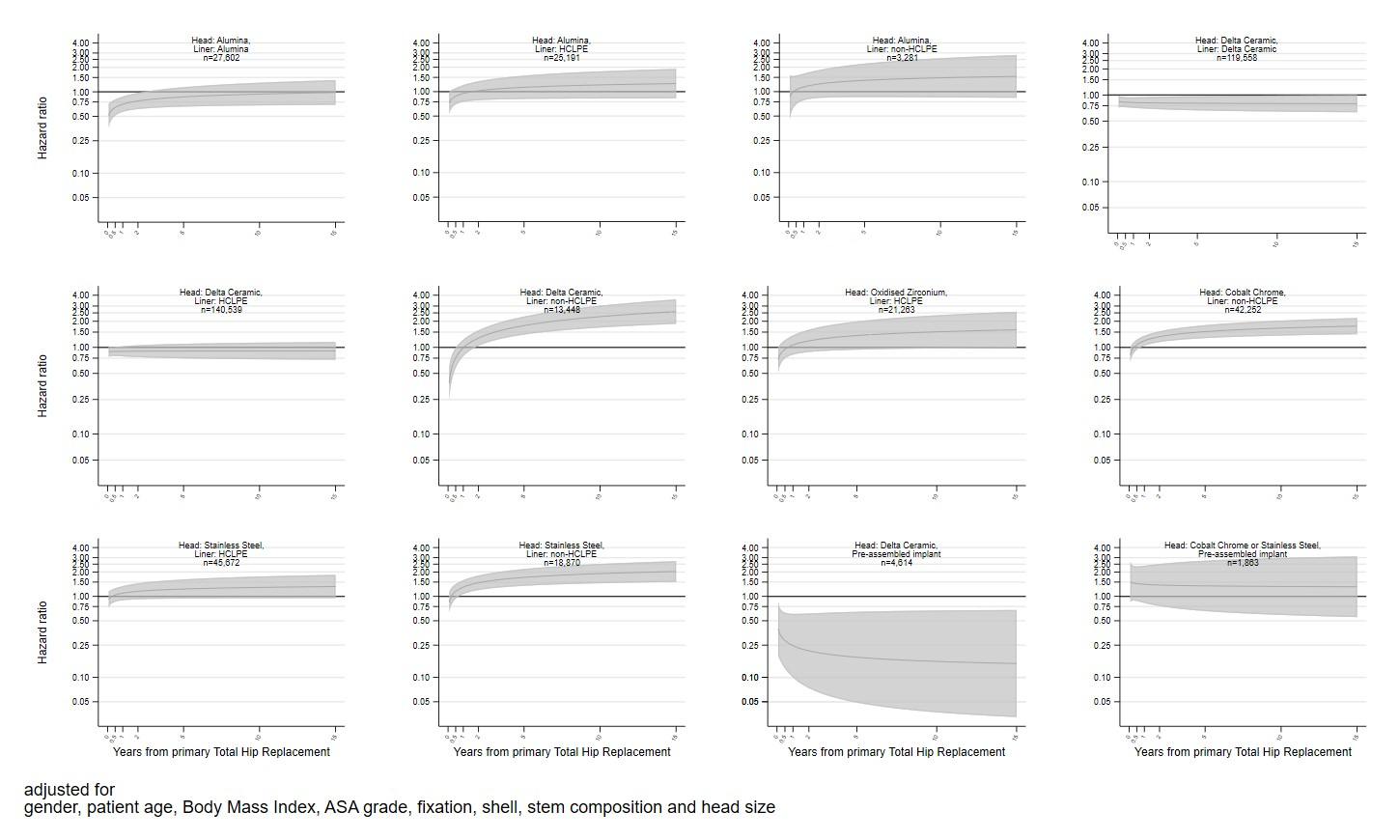


**Figure K:** Risk of revision for dislocation or subluxation by head and liner types (Reference: Cobalt chrome head with highly crosslinked polyethylene liner). Flexible parametric survival model adjusted for year of primary surgery, patient gender, age, Body Mass Index,  American Society of Anesthesiologists grade, implant fixation, shell composition, stem composition and head size. HCLPE: Highly CrossLinked PolyethylenE.

### Supplementary 9Pain

## Monobloc acetabular component


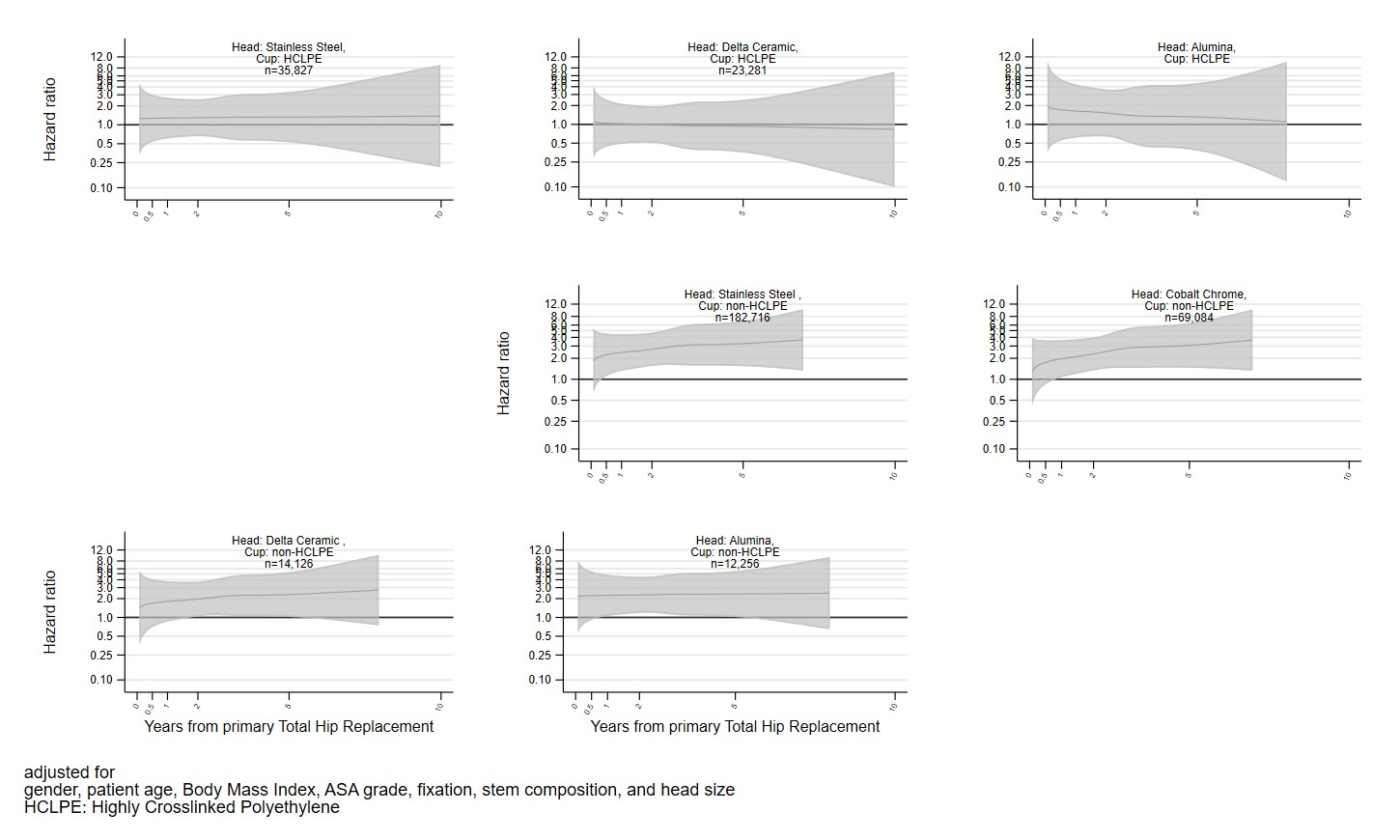


**Figure L:** Risk of revision for pain by head and cup types (Reference: Cobalt chrome head with highly crosslinked polyethylene cup). Flexible parametric survival model adjusted for year of primary surgery, patient gender, age, Body Mass Index,  American Society of Anesthesiologists grade, implant fixation, stem composition and head size. HCLPE: Highly CrossLinked PolyethylenE.

## Modular acetabular component


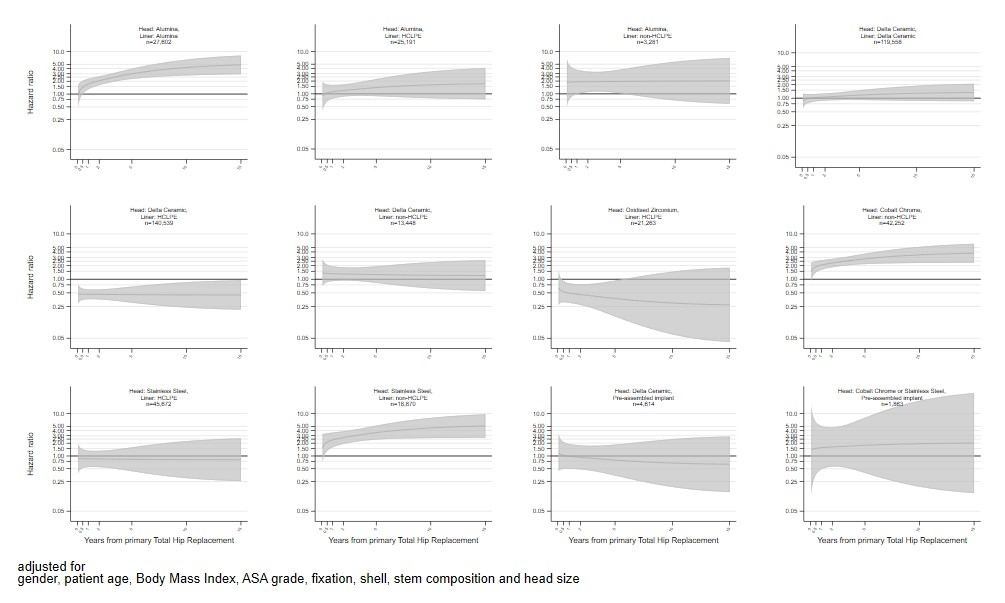


**Figure M:** Risk of revision for pain by head and liner types (Reference: Cobalt chrome head with highly crosslinked polyethylene liner). Flexible parametric survival model adjusted for year of primary surgery, patient gender, age, Body Mass Index,  American Society of Anesthesiologists grade, implant fixation, shell composition, stem composition and head size. HCLPE: Highly CrossLinked PolyethylenE.

### Supplementary 10: Infection

## Monobloc acetabular component


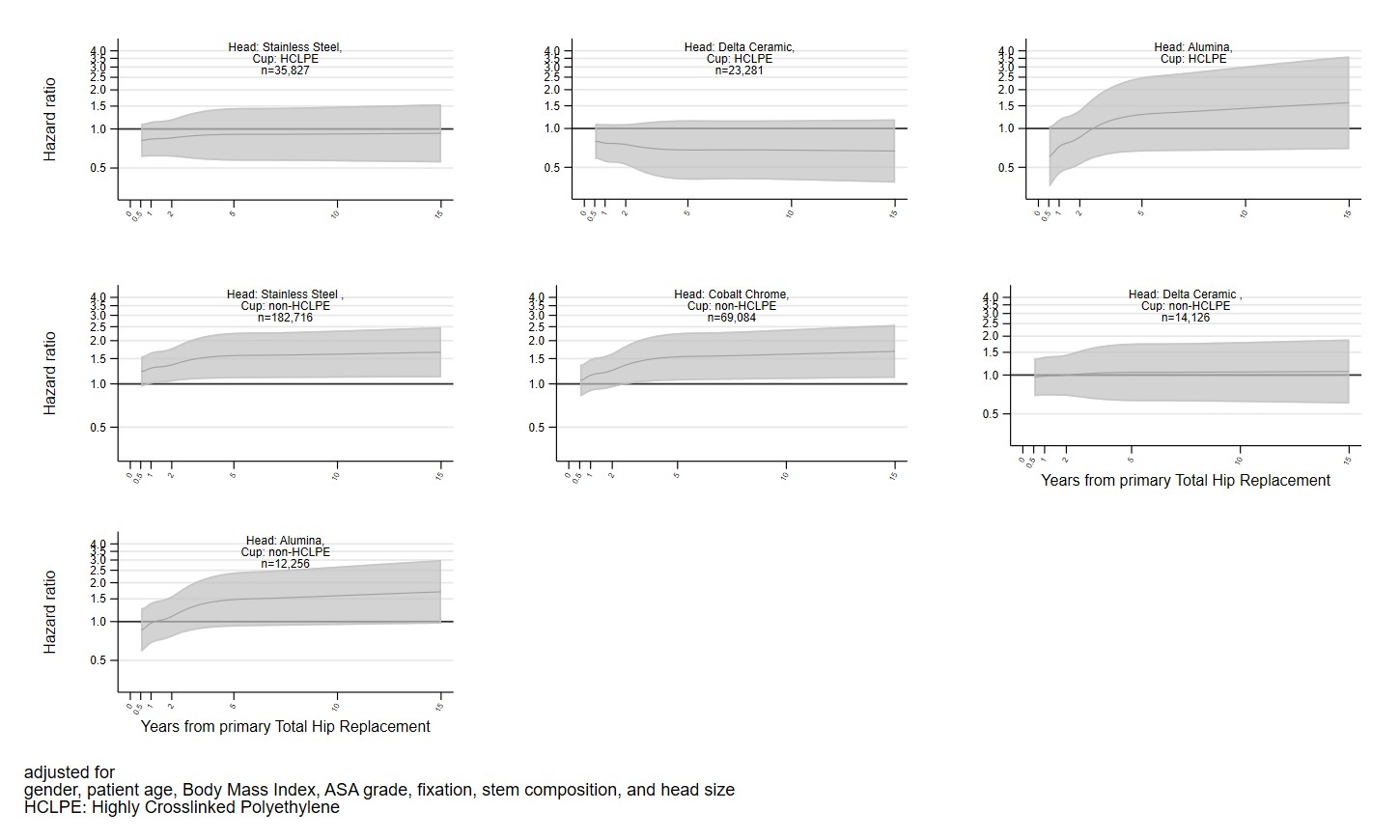


**Figure N:** Risk of revision for infection by head and cup types (Reference: Cobalt chrome head with highly crosslinked polyethylene cup). Flexible parametric survival model adjusted for year of primary surgery, patient gender, age, Body Mass Index,  American Society of Anesthesiologists grade, implant fixation, stem composition and head size. HCLPE: Highly CrossLinked PolyethylenE.

## Modular acetabular component


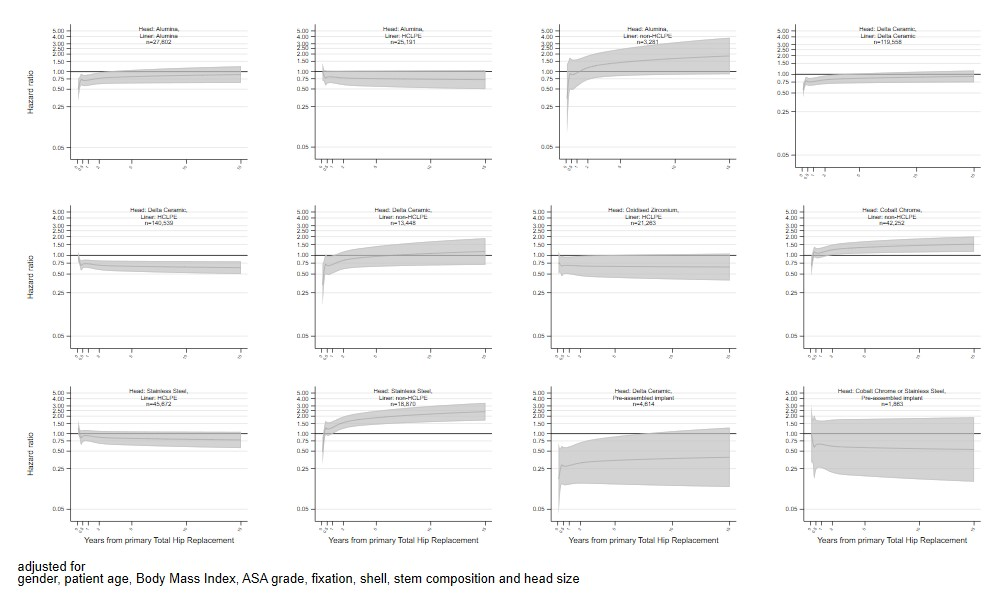


**Figure O:** Risk of revision for infection by head and liner types (Reference: Cobalt chrome head with highly crosslinked polyethylene liner). Flexible parametric survival model adjusted for year of primary surgery, patient gender, age, Body Mass Index,  American Society of Anesthesiologists grade, implant fixation, shell composition, stem composition and head size. HCLPE: Highly CrossLinked PolyethylenE.

### Supplementary 11: Any other reasons

## Monobloc acetabular component


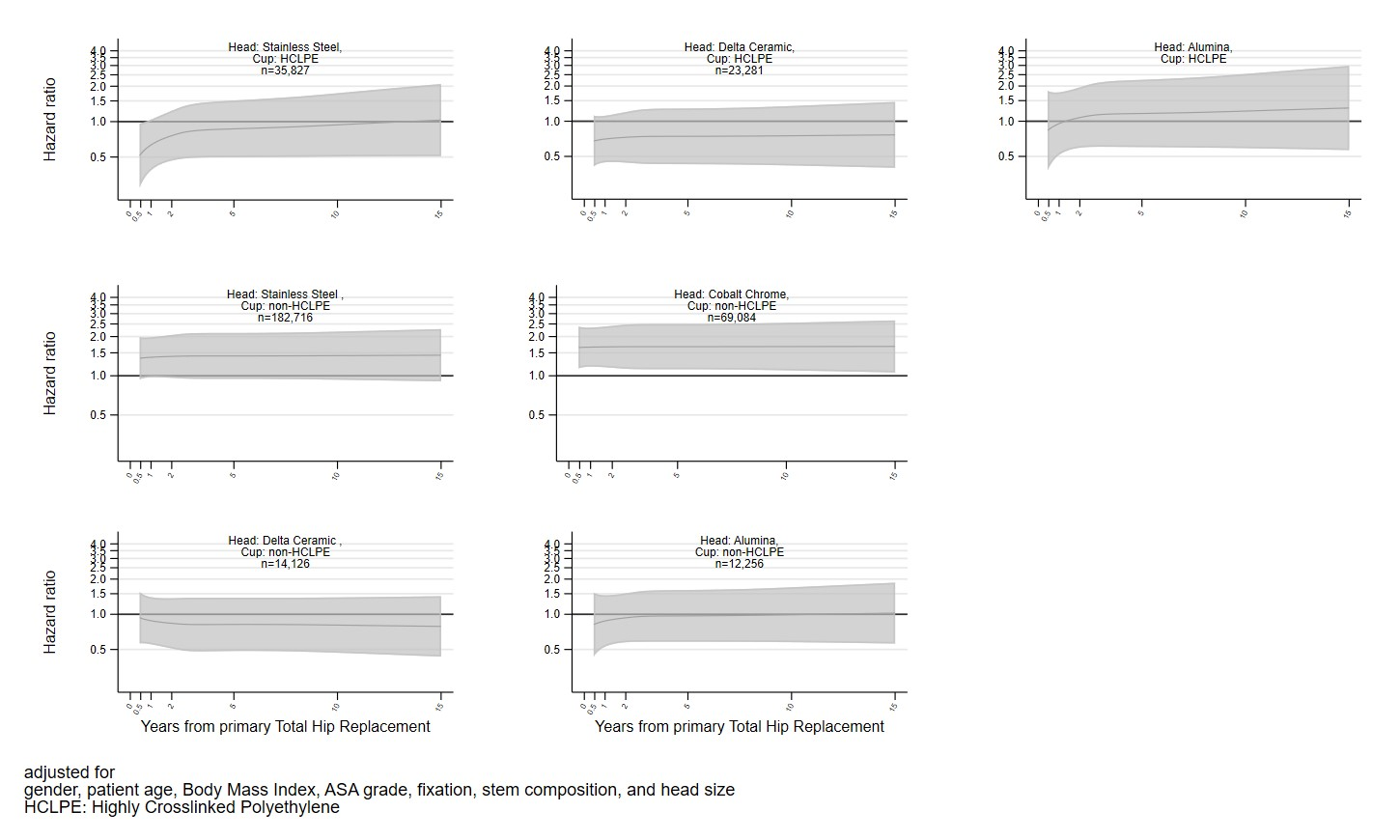


**Figure P:** Risk of revision for any other reason(s) by head and cup types (Reference: Cobalt chrome head with highly crosslinked polyethylene cup). Flexible parametric survival model adjusted for year of primary surgery, patient gender, age, Body Mass Index,  American Society of Anesthesiologists grade, implant fixation, stem composition and head size. HCLPE: Highly CrossLinked PolyethylenE.

## Modular acetabular component


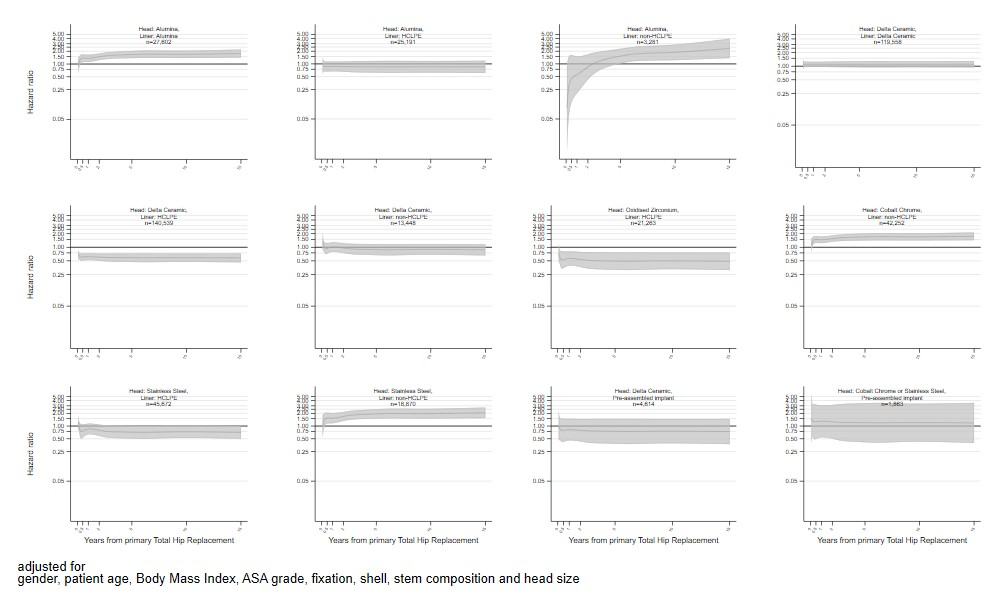
**Figure Q:** Risk of revision for any other reason(s) by head and liner types (Reference: Cobalt chrome head with highly crosslinked polyethylene liner). Flexible parametric survival model adjusted for year of primary surgery, patient gender, age, Body Mass Index,  American Society of Anesthesiologists grade, implant fixation, shell composition, stem composition and head size. HCLPE: Highly CrossLinked PolyethylenE.

### Supplementary 12: All-cause risk of revision for implants with delta ceramic head and Highly Cross-Linked PolyethylenE (HCLPE) or delta ceramic liner and oxidised zirconium head and HCPLE liner


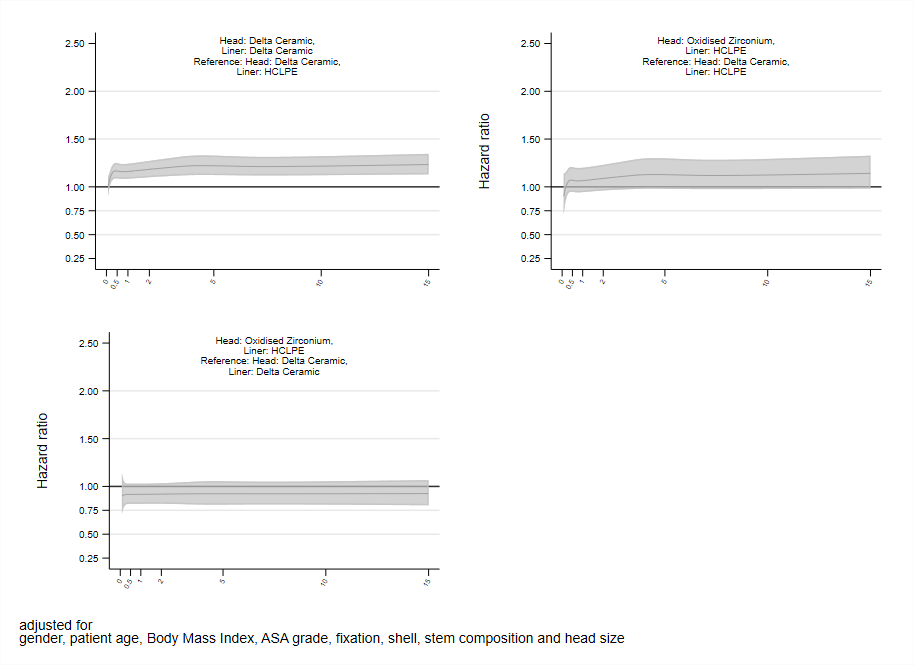


**Figure R:** Modular acetabular component-All-cause risk of revision for implants with delta ceramic head and Highly Cross-Linked PolyethylenE (HCLPE) and oxidised zirconium head and HCPLE liner. Flexible parametric survival model adjusted for year of primary surgery, patient gender, age, Body Mass Index,  American Society of Anesthesiologists grade, implant fixation, shell composition, stem composition and head size. HCLPE: Highly CrossLinked PolyethylenE.

### Supplementary 13

### **Table AA:** Registry studies identified in MEDLINE and Embase search on 1st March 2023

| **Study** | **Country** | **Metal** | **Ceramic** | **HCLPE** | **Other PE** | **Relevant to aim** | **Quality rating** |
| --- | --- | --- | --- | --- | --- | --- | --- |
| Babazadeh et al. 2021^1^ | Australia | Metal |  | HCLPE |  | No | 2 |
| Boyle et al. 2022^2^ | New Zealand |  |  |  |  | No | 2 |
| Castagnini et al. 2021^3^ | Italy | Metal | Ceramic | HCLPE | Other PE | Revision for dislocation only | 2 |
| Davis et al. 2020^4^ | NJR | Metal | Ceramic | HCLPE | Other PE | No | 2 |
| de Steiger et al. 2020^5^ | Australia | Metal | Ceramic | HCLPE |  | No | 2 |
| Deere et al. 2019^6^ | United Kingdom |  |  |  |  | No | 2 |
| Epinette et al. 2016^7^ | United Kingdom | Metal | Ceramic | HCLPE | Other PE | No | 2 |
| Etter et al. 2015^8^ | USA | Metal | Ceramic | PE |  | No | 2 |
| Gioe et al. 2011^9^ | USA | Metal | Ceramic | HCLPE | Other PE | No | 2 |
| Hallan et al. 2020^10^ | Norway | Metal | Ceramic | PE |  | Revision for fracture | 2 |
| Holleyman et al. 2021^11^ | United Kingdom | Metal | Ceramic | PE |  | No | 2 |
| Howard et al. 2017^12^ | United Kingdom |  | Ceramic |  |  | No | 2 |
| Jameson et al. 2015^13^ | United Kingdom | Metal | Ceramic | PE |  | No | 2 |
| Johanson et al. 2017^14^ | Nordic countries | Metal | Ceramic | HCLPE | Other PE | No | 2 |
| Junnila et al. 2016^15^ | Nordic countries |  |  |  |  | No | 2 |
| Kadar et al. 2012^16^ | Norway |  | Ceramic |  |  | No | 2 |
| Kendall et al. 2022^17^ | USA |  |  | HCLPE | Other PE | No | 2 |
| Khatod et al. 2006^18^ | USA |  |  |  |  | No | 2 |
| Kjaergaard et al. 2022^19^ | Denmark |  |  | HCLPE | Other PE | No | 2 |
| Krull et al. 2022^20^ | Germany |  |  | PE |  | No | 2 |
| McCalden et al. 2015^21^ | USA | Metal |  | HCLPE | Other PE | No | 2 |
| Necas et al. 2013^22^ | Slovakia |  |  |  |  | No | 2 |
| Partridge et al. 2020^23^ | United Kingdom | Metal |  | HCLPE | Other PE | No | 2 |
| Paxton et al. 2012^24^ | USA | Metal | Ceramic | HCLPE | Other PE | No | 2 |
| Paxton et al. 2015^25^ | USA |  |  | HCLPE | Other PE | No | 2 |
| Peters et al. 2018^26^ | The Netherlands | Metal | Ceramic | HCLPE | Other PE | Yes, but limited breakdown of material type | 2 |
| Sharplin et al. 2018^27^ | New Zealand | Metal | Ceramic | HCLPE | Other PE | Yes but no details of material type | 2 |
| Sheridan et al. 2019^28^ | Ireland | Metal | Ceramic | PE |  | No | 2 |
| van Loon et al. 2023^29^ | The Netherlands | Metal | Ceramic | PE |  | No | 2 |

NJR: National Joint Registry; USA: United States of America; HCLPE: Highly Cross-Linked PolyEthylene; PE: PolyEthylene

References

1. Babazadeh S, de Steiger RN, Peng Y, van Bavel D. Stainless Steel Femoral Heads Reduce Rate of Revision When Compared to Ion-Implanted Chromium-Cobalt Heads With a Single Cemented Femoral Design: An Analysis of 40,468 Total Hip Replacements From the Australian Orthopedic Association National Joint Replacement Registry. J Arthroplasty 2021; 36(12): 3945-9.

2. Boyle AB, Zhu M, Frampton C, Poutawera V, Vane A. Comparing modern uncemented, hybrid and cemented implant combinations in older patients undergoing primary total hip arthroplasty, a New Zealand Joint Registry study. Arch Orthop Trauma Surg 2022.

3. Castagnini F, Bordini B, Cosentino M, et al. The influence of bearing surfaces on revisions due to dislocations in total hip arthroplasty. J Mater Sci Mater Med 2021; 32(9): 123.

4. Davis ET, Pagkalos J, Kopjar B. Effect of Bearing Surface on Survival of Cementless and Hybrid Total Hip Arthroplasty: Study of Data in the National Joint Registry for England, Wales, Northern Ireland and the Isle of Man. JB JS Open Access 2020; 5(2): e0075.

5. de Steiger RN, Hatton A, Peng Y, Graves S. What Is the Risk of THA Revision for ARMD in Patients with Non-metal-on-metal Bearings? A Study from the Australian National Joint Replacement Registry. Clin Orthop Relat Res 2020; 478(6): 1244-53.

6. Deere KC, Whitehouse MR, Porter M, Blom AW, Sayers A. Assessing the non-inferiority of prosthesis constructs used in hip replacement using data from the National Joint Registry of England, Wales, Northern Ireland and the Isle of Man: a benchmarking study. BMJ Open 2019; 9(4): e026685.

7. Epinette JA, Jolles-Haeberli BM. Comparative Results From a National Joint Registry Hip Data Set of a New Cross-Linked Annealed Polyethylene vs Both Conventional Polyethylene and Ceramic Bearings. J Arthroplasty 2016; 31(7): 1483-91.

8. Etter K, Sedrakyan A, Rubin LE, Resnik L, Wilson I, Dore DD. Effect of Bearing Surface Material on Surgical Revision Rates in Total Hip Replacements: A Propensity Score-matched Cohort from the Veterans Health Administration. Pharmacoepidemiology and Drug Safety 2015; 24: 298-.

9. Gioe TJ, Sharma A, Tatman P, Mehle S. Do "premium" joint implants add value?: analysis of high cost joint implants in a community registry. Clin Orthop Relat Res 2011; 469(1): 48-54.

10. Hallan G, Fenstad AM, Furnes O. What Is the Frequency of Fracture of Ceramic Components in THA? Results from the Norwegian Arthroplasty Register from 1997 to 2017. Clin Orthop Relat Res 2020; 478(6): 1254-61.

11. Holleyman RJ, Critchley RJ, Mason JM, Jameson SS, Reed MR, Malviya A. Ceramic Bearings Are Associated With a Significantly Reduced Revision Rate in Primary Hip Arthroplasty: An Analysis From the National Joint Registry for England, Wales, Northern Ireland, and the Isle of Man. J Arthroplasty 2021; 36(10): 3498-506.

12. Howard DP, Wall PDH, Fernandez MA, Parsons H, Howard PW. Ceramic-on-ceramic bearing fractures in total hip arthroplasty: an analysis of data from the National Joint Registry. Bone Joint J 2017; 99-B(8): 1012-9.

13. Jameson SS, Mason J, Baker PN, Gregg PJ, Deehan DJ, Reed MR. Implant Optimisation for Primary Hip Replacement in Patients over 60 Years with Osteoarthritis: A Cohort Study of Clinical Outcomes and Implant Costs Using Data from England and Wales. PloS one 2015; 10(11): e0140309.

14. Johanson PE, Furnes O, Ivar Havelin L, et al. Outcome in design-specific comparisons between highly crosslinked and conventional polyethylene in total hip arthroplasty. Acta Orthop 2017; 88(4): 363-9.

15. Junnila M, Laaksonen I, Eskelinen A, et al. Implant survival of the most common cemented total hip devices from the Nordic Arthroplasty Register Association database. Acta Orthop 2016; 87(6): 546-53.

16. Kadar T, Dybvik E, Hallan G, Furnes O, Havelin LI. Head material influences survival of a cemented total hip prosthesis in the Norwegian Arthroplasty Register. Clin Orthop Relat Res 2012; 470(11): 3007-13.

17. Kendall J, Pelt CE, Imlay B, Yep P, Mullen K, Kagan R. No Reduction in Revision Risk Associated With Highly Cross-linked Polyethylene With or Without Antioxidants Over Conventional Polyetheylene in TKA: An Analysis From the American Joint Replacement Registry. Clin Orthop Relat Res 2022; 480(10): 1929-36.

18. Khatod M, Barber T, Paxton E, Namba R, Fithian D. An analysis of the risk of hip dislocation with a contemporary total joint registry. Clin Orthop Relat Res 2006; 447: 19-23.

19. Kjaergaard K, Varnum C, Ding M, Overgaard S. Revision Risk of Total Hip Arthroplasty With Vitamin E Doped Liners: Results From the Danish Hip Arthroplasty Register. J Arthroplasty 2022; 37(6): 1136-42.

20. Krull P, Steinbruck A, Grimberg AW, Melsheimer O, Morlock M, Perka C. Modified acetabular component liner designs are not superior to standard liners at reducing the risk of revision : an analysis of 151,096 cementless total hip arthroplasties from the German Arthroplasty Registry. Bone Joint J 2022; 104-B(7): 801-10.

21. McCalden RW. CORR Insights (R): Metal-on-conventional total hip arthroplasty bearing surfaces have a higher risk of revision than metal-on-highly crosslinked polyethylene: results from a US registry. Clin Orthop Relat Res 2015; 473(4): 1449-50.

22. Necas L, Katina S, Uhlarova J, Colton CL. Slovakian arthroplasty register: Survival analysis of total hip and knee replacement in Slovakia 2003-2011. Acta Chirurgiae Orthopaedicae et Traumatologiae Cechoslovaca 2013; 80(SUPPL.): 1-85.

23. Partridge TCJ, Baker PN, Jameson SS, Mason J, Reed MR, Deehan DJ. Conventional Versus Highly Cross-Linked Polyethylene in Primary Total Knee Replacement: A Comparison of Revision Rates Using Data from the National Joint Registry for England, Wales, and Northern Ireland. J Bone Joint Surg Am 2020; 102(2): 119-27.

24. Paxton EW, Ake CF, Inacio MC, Khatod M, Marinac-Dabic D, Sedrakyan A. Evaluation of total hip arthroplasty devices using a total joint replacement registry. Pharmacoepidemiol Drug Saf 2012; 21 Suppl 2: 53-9.

25. Paxton EW, Inacio MC, Namba RS, Love R, Kurtz SM. Metal-on-conventional polyethylene total hip arthroplasty bearing surfaces have a higher risk of revision than metal-on-highly crosslinked polyethylene: results from a US registry. Clin Orthop Relat Res 2015; 473(3): 1011-21.

26. Peters RM, Van Steenbergen LN, Stevens M, Rijk PC, Bulstra SK, Zijlstra WP. The effect of bearing type on the outcome of total hip arthroplasty. Acta Orthop 2018; 89(2): 163-9.

27. Sharplin P, Wyatt MC, Rothwell A, Frampton C, Hooper G. Which is the best bearing surface for primary total hip replacement? A New Zealand Joint Registry study. Hip int 2018; 28(4): 352-62.

28. Sheridan GA, Kelly RM, McDonnell SM, Walsh F, O'Byrne JM, Kenny PJ. Primary total hip arthroplasty: registry data for fixation methods and bearing options at a minimum of 10 years. Ir J Med Sci 2019; 188(3): 873-7.

29. van Loon J, Sierevelt IN, Spekenbrink-Spooren A, et al. Higher risk of 2-year cup revision of ceramic-on-ceramic versus ceramic-on-polyethylene bearing: analysis of 33,454 primary press-fit total hip arthroplasties registered in the Dutch Arthroplasty Register (LROI). Hip int 2023; 33(2): 280-7.
