# Supplementary material for: The association of bearing surface materials with the risk of revision following primary total hip replacement: A cohort analysis of 1,026,481 hip replacements from the National Joint Registry
Source: PLoS Med. 2024 Nov 7;21(11):e1004478. doi: 10.1371/journal.pmed.1004478 (PMC11542800; doi:10.1371/journal.pmed.1004478)
Supplement: Attachment — Submitted filename: response to reviewers final.docx [file pmed.1004478.s003.docx]

Editorial comments:

The editorial team all agree that this is an interesting piece of work that could be very impactful, and we are grateful that you chose to submit this to PLOS Medicine. However, we agree with the reviewers’ comments and have some concerns that will need to be addressed in full before your paper can be taken forward.

Thank you for the opportunity to revise our manuscript. We believe we have amended it as per the reviewer comments and the manuscript is clearer, better presented and therefore more likely to be impactful.

1) Thank you for stating that the study was funded by CeramTec. To ensure transparency and satisfy the team that the function of the sponsor does not preclude publication, it would be useful to add some detail on what the funding was used for, specifically, in the completion of this study.

Following the signature of a research contract between the University of Bristol and Ceramtec, Ceramtec paid a grant to the University of Bristol that was used to conduct the presented research. Like a public grant, it:

-has been used to pay the data access fees;

-is received by the University of Bristol and used by this institution to pay the researcher salaries;

-will be used to pay the publication cost, including dissemination to conferences

None of the involved researchers has received direct funding from Ceramtec, or any other advantages, or by a third party related to Ceramtec. This work, including the research output, has been approved, by the HQIP-NJR board, ie the data provider. The research team has total freedom to publish the results and no restrictions to the data selected, analysed and to the output have been applied. The function of the funder has not precluded publication.

2) Please revise your statistical analyses to address the influence of confounding variables more robustly, e.g. using rigorous sensitivity analyses. Note the comments and suggestions offered by Reviewer 3, who is a statistician.
We have revised the manuscript as recommended by the reviewers, in particular conducted further adjustments. The results have remained similar. We have also answered reviewer 3 comments. Please let us know if further amendments were required.

3) Please provide a description of the formal or informal processes that might go into the decision-making process when bearing materials are chosen, with an accompanying critical discussion. This would help to address any concerns that readers might have about confounding by indication here. In your discussion, it would be useful to hear what factors specifically could have influenced decision-making – could this have been influenced by patient age or the life expectancy of the implant, for example? Our readership is global, so it would also be useful to state whether patients in the UK might have any choice over the materials used in their implants.
Thank you for this recommendation. We have added further details in the discussion that should clarify those points. In short, patient characteristics and, very rarely, preferences, as well as surgeons preferences, play very little role in the “decision-making” process: this later is typically made, independently, by hospital board for economic reasons, loosely influenced by research evidence. Our analyses were adjusted for patient age and this characteristics is therefore controlled for in the results outputted.

“In the United Kingdom, surgeon choice is heavily constrained at unit level due to units carrying a selected range of implants imposed by hospital board regulations, mostly for economic reasons. The indication specific revision analyses (sFigure 2-17 ) is giving further insight into the role of each bearing surface materials on the considered outcome, and were aligned with the overall revision analyses (Figures 1 and 2)”

4) Data Availability:
The Data Availability Statement (DAS) requires some revision – please review the first sentence. Also, please describe briefly the ethical, legal, or contractual restriction that prevents you from sharing deidentified data.

These regulations are those of the data providers and not ours, and applied to all projects irrespectively of their fundings. These data are available to all those who will satisfy HQIP-NJR regulations but will have to be managed in their data “safe heaven”: the concept of “deidentified” dataset is not yet available and it is mandatory to use this safe heaven. As data user we have no say in this process. We have modified the statement to clarify the statements:

The research project was reviewed and approved by the HQIP-National Joint Registry board and the data were analysed in a data safe heaven approved by this data provider. The studied data and related statistical codes are archived on the University of Bristol network. The related files remains accessible to all those with due HQIP-National Joint Registry permissions that are beyond our control. Further information provided by the data controller can be accessed here:https://www.njrcentre.org.uk/research/research-requests/ contains information on research data access request to the National Joint Registry.
The authors will share all codes and data with those who hold permission from the National Joint Registry, as per this data controller requirements.

5) Reporting guidance:
Thank you for including the STROBE checklist as Supporting Information. Please add the following statement, or similar, to the Methods: "This study is reported as per the ‘Strengthening the Reporting of Observational Studies in Epidemiology (STROBE): guidelines for reporting observational studies’ Statement” and refer to the checklist’s location.

When completing the checklist, please use section and paragraph numbers, rather than page numbers.

Further guidance can be found here: <https://www.equator-network.org/reporting-guidelines/>

Completed as recommended

6) Statistical reporting:
Please quantify the main results with 95% CIs and p values.
When reporting p values please report as <0.001 and where higher as p=0.002, for example. When reporting 95% CIs please separate upper and lower bounds with commas instead of hyphens as the latter can be confused with reporting of negative values.
Please include the actual amounts and/or absolute risk(s) of relevant outcomes (including NNT or NNH where appropriate), not just relative risks or correlation coefficients. (example for absolute risks: PMID: 28399126).
Please include any important dependent variables that are adjusted for in the analyses.

We have now added the p values in all tables according the above formatting.

7) Prespecified analysis plan/study protocol:
Did your study have a prospective protocol or analysis plan? Please state this (either way) early in the Methods section.

a) If a prospective analysis plan (from your funding proposal, IRB or other ethics committee submission, study protocol, or other planning document written before analyzing the data) was used in designing the study, please include the relevant prospectively written document with your revised manuscript as a Supporting Information file to be published alongside your study, and cite it in the Methods section. A legend for this file should be included at the end of your manuscript.

b) If no such document exists, please make sure that the Methods section transparently describes when analyses were planned, and when/why any data-driven changes to analyses took place.

c) In either case, changes in the analysis-- including those made in response to peer review comments-- should be identified as such in the Methods section of the paper, with rationale.

Added as per above requirements.

For all observational studies, in the manuscript text, please indicate: (i) the specific hypotheses you intended to test, (ii) the analytical methods by which you planned to test them, (iii) the analyses you actually performed, and (iv) when reported analyses differ from those that were planned, transparent explanations for differences that affect the reliability of the study's results. If a reported analysis was performed based on an interesting but unanticipated pattern in the data, please be clear that the analysis was data-driven.

We believe our reporting is congruent with these requirements

8) Author summary:
At this stage, we ask that you include a short, non-technical Author Summary of your research to make findings accessible to a wide audience that includes both scientists and non-scientists. The authors summary should consist of 2-3 succinct bullet points under each of the following headings:
• Why Was This Study Done? Authors should reflect on what was known about the topic before the research was published and why the research was needed.
• What Did the Researchers Do and Find? Authors should briefly describe the study design that was used and the study’s major findings. Do include the headline numbers from the study, such as the sample size and key findings.
• What Do These Findings Mean? Authors should reflect on the new knowledge generated by the research and the implications for practice, research, policy, or public health. Authors should also consider how the interpretation of the study’s findings may be affected by the study limitations. In the final bullet point of ‘What Do These Findings Mean?’, please describe the main limitations of the study in non-technical language.
The Author Summary should immediately follow the Abstract in your revised manuscript. This text is subject to editorial change and should be distinct from the scientific abstract. Please see our author guidelines for more information: https://journals.plos.org/plosmedicine/s/revising-your-manuscript#loc-author-summary

Added
-----------------------------------------------------------
Comments from the reviewers:

REVIEWER #1:

Thank you for your comments.

"unit of analysis for the consideration of revision outcomes is the implant (rather than patient) so we included 260,830 primary procedures performed on contralateral sides of the same patient but on different dates. "
Clustering by patient considered?

The data in the National Joint Registry (NJR) are clustered within patients (prostheses (left or right side), surgeons, and hospitals. When performing survival analysis, accounting for this data structure, or a simpler clustering structure such as prosthesis surgery within hospital is currently impossible: The NRJ dataset is too large (over 1 million records) and such modelling is never converging, leading to system crash. This is particularly true for the modelling that we had to use in this output: multilevel flexible survival analysis statistical commands do not yet allow the modelling of datasets of this size.
Moreover, around 84% of the patients had received only one primary hip prosthesis, and the remaining 16% had received two primary prosthesis on either side, ie there were two observations per patient maximum. Irrespectively of the outcome distribution, with such a “weak” clustering structure, modelling these data with a multilevel model is highly computer-intensive with very little improvement compared to a model ignoring such clustering structure (see my publication [Modelling the hierarchical structure of road crash data—Application to severity analysis - ScienceDirect](https://www.sciencedirect.com/science/article/pii/S000145750500117X)).
In previous studies of the NJR data, not using survival modelling, we attempted to model the clustering of patients within surgeons and the resulting estimations were similar to the non-clustered analyses results ([Risk factors associated with revision for prosthetic joint infection following knee replacement: an observational cohort study from England and Wales (thelancet.com)](https://www.thelancet.com/action/showPdf?pii=S1473-3099%2818%2930755-2)). There we modelled a simpler clustering structure, i.e. implants within surgical units. It was only possible because we had not used a survival modelling and a non-informative Bayesian structure, that required several weeks per model to compute. Very little difference compared to ignoring the multilevel structure of the NJR data was observed.

For all these reasons, and that survival analysis, and specifically flexible survival analysis, is the most appropriate modelling strategy for the presented research, we have not modelled the NJR clustering structure in the analyses. We have already acknowledged the potential limitation of the modelling strategy we have had to selected:
“Shared operative strategies, or bearing surface selection by surgeons, within the same surgical unit, may generate some clustering at unit level. Previous investigations on the same dataset accounting for unit level clustering added little value to the analyses and their modelling is currently challenging with large dataset in the context of survival analysis.(22)”

REVIEWER #2:

Thank you for your comments.
"Restricted cubic spline function used to model the baseline function" - suggest changing "baseline function" to "baseline hazard" for clarity…"the time-dependent effects associated with bearing materials combination only were determined using the smallest Akaike Information Criterion (AIC) and Bayesian Information Criterion (BIC)." - I do not completely understand what you mean here - did you investigate different forms of the restricted cubic splines (e.g., number/placement of knots) and then use AIC/BIC to determine best fit?...

Thank you for these recommendations. We have now modified the text to clarify those points: “Restricted cubic splines were used to model the baseline hazard function and the time-dependent effects associated with the bearing materials combination. For each model, the best fitting model with the most parsimonious number of knots was determined using the smallest Akaike Information Criterion (AIC) and Bayesian Information Criterion (‎BIC).”

Did you consider adjusting for year of primary THA? It would seem that trends in implant type would have changed from 2003 - 2019, with some implant types more likely to be used in earlier years and other in later years.

As recommended the models have been adjusted for year of primary surgery. The year of primary surgery had no effect on the studied outcomes and therefore the adjusted associations between these outcomes and bearing materials: The results are unchanged. We thank you for this recommendation as this gives more robustness to the results and conclusions.

How did the models account for the clustered nature of the data (potential for multiple implants per patient)?

Please see above comment in response to reviewer 1

Did the analyses for specific revision reasons include the competing risk of other revision reasons?
As shown by some of the co-authors in [Are competing risks models appropriate to describe implant failure? (tandfonline.com)](https://www.tandfonline.com/doi/pdf/10.1080/17453674.2018.1444876), the use of competing risks is inappropriate when the perspective is on surgeons who are attempting to select the best implants with the lowest failure rates for their patients. Competing risks should be used when adopting a patient perspective who needs to consider the different risk they may face over the life-course, in addition to the hip replacement.
As this paper is focussing on the choice of prosthesis materials at the time of surgery, irrespectively of what may occur to patient thereafter, that cannot be known at this stage, the modelling of the competing risk has been deemed inappropriate and was not considered in the presented results.

REVIEWER#3:
This is an interesting study on the association of bearing surface materials with the risk of revision following primary total hip replacement using data from a National Joint Registry. However, there are a few major issues needing attention.

1. The paper is difficult to follow especially for non-expert in the hip replacement field. With so many sub-categories and combinations between them (table 1), it is easy to get lost as it's not clear why the reference was chosen and why those comparisons were chosen (table 2).

Unfortunately, there are many different bearing combinations in regular use for total hip replacement. We have minimised the combinations as far as possible to the traditional and most common categories used by the international orthopaedic community, strictly reflecting the current clinical practices. Orthopaedic surgery is one of the most, if not the most common elective surgery, in developed countries. We believe the current results presentation will be directly relevant to this large audience, as well as orthopaedic ward and hospital management groups and broader area care commissioners/organisers. Reducing the number of categories may simplify the results outputting but would lead to groupings 1. that do not reflect real-life clinical practice and 2. would put together constructs that have important differences, thus reducing the validity of comparisons.

As presented our results do strictly mirror the range of options that surgeons have to choose from, and any further groupings would provide conclusions with very little practical application to the practitioner and care organiser communities. We would therefore prefer to stick to the current reporting formatting which, we believe, produce findings that will be directly translatable, and therefore highly citable, in the clinical community.

The reference category, cobalt chrome head with highly-crosslinked polyethylene cup/liner, has been selected because this was the most commonly implant bearing surface combination used over the studied period. It is one of the most popular implant constructs in numerous national health organisation, such as the United Kingdom National Health Service or the care organisations in the Nordic countries.

2. Study design. Ideally RCTs are perferred to cohort studies for this type of study as there are many confounding factors assiciated the risk of revision following THR. Unless adjusted comprehensively for all the potential confounders, the results are subject to scrutiny. For example, year of hip replacement, ethnicity, socioeconomic status, drinking, smoking, multimorbidity, hip replacement procedures, medication and treatment in the follow-ups, all these factors could contribute to the revision but unfortunately not included in the analyses.

On one hand RCT are at a higher position in the pyramid of evidence, providing stronger results than observational studies. On the other hand, RCT produces results with the highest internal validity but with limited external validity: the results can only be generalised to the site(s) involved in the (multicentre-) trial, but it is not guaranteed that they would be straightforwardly generalisable to the other sites of the concerned health care system. Moreover, there is limited clinical equipoise when it comes to hip implants component choice as choices are typically constrained by hospital board, on financial/contractual reason, and surgeon preference. Although revision surgery is a significant event both economically and for patients, on an individual level it remains rare with small absolute risk differences between groups, observable over long years or decades. Designing an appropriately powered RCT would require huge numbers and resource and is unlikely to be cost-effective, despite this being a vital question with large benefits seen in the long term by reducing revision rates on a population/commissioner level. An RCT is therefore extremely unlikely to be feasible in this context, with debateable clinical equipoise, less generalisable results that do not reflect real-world practice, with results not seen for a decade or more.

Our results are obtained from readily-available, prospectively collected data from one of the largest national arthroplasty registries in the world, providing information across two whole health systems (England and Wales), including public and private sectors, spanning over 15 years, allowing for comparison of all types of implanted hip prostheses. This has produced findings with high generalisability, reflecting the actual effectiveness of these implants over a long time period, over an RCT which would be far more constrained in terms of time and surgeon/hospital engagement.

Research conducted on the National Joint Registry (NJR), and therefore the data used in this research, has lead to the worldwide decommissioning of the dangerous hip metal-on-metal implant, and allowed us to also produce world-leading results on the provision of hip and knee joint replacement and social inequalities in this provision, the duration of hip implants or the risk factors of prosthesis hip infection to cite a few of the NJR outputs with international impact.

Whilst the analyses conducted in this particular article have been revised to adjust for year of hip replacement, with no change in the results, and are adjusted for the American Society of Anesthesiologists (ASA) grade, a measure of comorbidities, not all other factors are captured in the NJR and residual confounding cannot be ruled out. We have already acknowledged this limitation in the discussion: “However, these results could be influenced by residual confounding as not all factors that could influence implant selection or the risk of revision are captured in the NJR. “

3. Statistical analyses. Flexible parametric survival models were used to reflect the time-dependant nature of HRs, which is mostly adequate. However, as the outcome is revision rather than all-cause mortality, there is a competing risk issue (eg. from death or not qualified for a revision due to deteriorating health) which was not addressed in the paper.

As shown by some of the co-authors in [Are competing risks models appropriate to describe implant failure? (tandfonline.com)](https://www.tandfonline.com/doi/pdf/10.1080/17453674.2018.1444876), the use of competing risks models is inappropriate when the aim is to select the best implants with the lowest failure rates for patients. Competing risks models are appropriate, for example, on an individual-patient level where the aim is to estimate the chance of a revision within that patient’s lifetime. As this paper is focussing on how the choice of prosthesis materials at the time of surgery influences implant longevity, irrespective of what may occur to patient thereafter (and as far as we know mortality satisfies the non-informative censoring assumption), competing risks models are inappropriate and were not considered in the presented results.

4. The abstract was poorly written. There is no HRs or P-values in the findings when higher or lower was claimed. As there were so many comparisons, it became difficult to follow what the findings are, what the conclusions are and what the interpretations are. Basically, it is difficult to find and understand key messages from the paper.

Please accept our apologies for these issues. We hope the abstract is now clearer.

REVIEWER #4:
Thank you for letting me review this interesting paper. The authors have compared the risk of revision between different articulations in a national registry setting. Extensive statistical analyses have been carried out. The paper is well written, the aim is well described, the subject is timely and the material is substantial. Main findings were that cross-linked PE was better than non-crosslinked PE, delta ceramic and Oxinium was superior to CoCr, and so was Alumina and stainless steel. Differences were found for 'all revisions' and for 'indication-specific revisions' and were mostly observed in the first years post implantation.

The manuscript leaves me with some questions: Is a difference in dislocation, loosening, fracture or infection rate the first few years after THR caused by the articulating materials? From a clinical point of view, I would be surprised to find that the materials were responsible. In this paper no possible explanations for the findings are discussed. The reader is left with the conclusion that ceramics lower the risk of early dislocation, infection and so on. This could of course be the case, but other factors, such as time dependent factors that may or may not be adjusted for, could also affect the findings. Could poor stem or cup makes be distributed disproportionally between the groups? Differences in median follow-up could affect the risk of revision for infection, and maybe other causes also. I believe this should be explored. A critical discussion of findings that are not easily explainable from a clinical point of view would be appreciated. This is maybe particularly important because the study is sponsored by the biggest player in the ceramic industry. I suggest doing some kind of sensitivity analysis, for instance the authors may try to study a subset of patients operated in the same time period, with the same implants, in similar patients (i.e. OA, ASA 1-2, same age distribution) but with different articulating surfaces. If the findings are replicated in such sub-analyses this would certainly strengthen the results.

Thank you for highlighting the lack of discussion around the potential mechanisms by which the articulating materials can influence implant survival. We have now added a paragraph to address this; “The mechanisms by which bearing materials might influence implant longevity are multifaceted. For the acetabular component, HCLPE has been demonstrated to have significantly reduced wear compared to non-HCLPE, reducing late failure by mitigation of particle-induced periprosthetic osteolysis which can lead to implant instability and pain.(58, 59) Ceramic materials have been shown in vitro to have reduced bacterial adhesion and slower biofilm development compared to metals due to their surface properties(60). This could influence earlier revision rates both for infection and other causes as low-grade infection can masquerade as aseptic loosening and instability.(61, 62) Use of a ceramic head reduces corrosion at the trunnion (the modular interface between the head and the stem), which can cause adverse reaction to metal debris requiring revision surgery.(63)”

Primary and revision hip arthroplasty performed in England and Wales are recorded in the National Joint Registry (NJR). We have therefore a record of the follow-up of each participant starting from the primary hip procedure and finishing at the time of the revision procedure, time of death also recorded in the NJR, or at the end of the study observation if the patient has not been revised or is alive. To account for the variation in patient follow-ups, we have used survival analysis. Our modelling approach has therefore accounted for the difference in patient (median) follow-up.

We have chosen to analyse the whole dataset rather than to conduct a matched study design for a number of reasons. Practically, as touched on above there are hundreds of different component combinations in use, which potentially different methods of fixation (cemented/uncemented/hybrid/reverse hybrid) that we have consolidated into groups as much as possible to retain practical application whilst remaining digestible to the reader. Taking subsets of patients and implant combinations might appear on the surface to offer direct comparisons, but actually will a) limit comparisons and therefore results and the scope of the paper b) reduce generalisability and c) exaggerate potential bias (please see <https://gking.harvard.edu/publications/why-propensity-scores-should-not-be-used-formatching>). We have therefore instead approached the analysis by including the whole study population and adjusting for potential confounders including age, year of operation, ASA grade, BMI, fixation type, stem composition, femoral head size, and acetabular shell composition rather than aiming to control for some of these factors via a matching process. This has allowed us to analyse the whole registry dataset and perform comparisons between the different bearing surface materials groups with appropriate adjustment.

Page 4, line 11. Needs a reference
Thank you – references added.

Page 4, line 15. Please see NARA study. Mikkelsen RT et al, Acta Orthop 2023

Thanks reference added.

Page 5, line 3-7. See Mikkelsen study

While the Mikkelsen study has definitively added to the field, this research is not breaking down the traditional construct grouping used in arthroplasty research: metal or ceramic head with metal, ceramic or highly crosslinked-polyethylene liner/cup versus the finer grouping conducted in our research in which the metal group is broken down into stainless steel head, less commonly used in numerous countries, and cobalt chrome head, the most common head materials in the United Kingdom or other Nordic countries; the ceramic group is broken down between alumina, the older ceramic material materials, and delta ceramic, the contemporaneous one with different properties. For these reasons, we believe it would be inappropriate to cite this reference here.

Page 7, line. I am a bit confused about the pre-assembled cups; where they grouped with the non-HCLPE liners as stated in line 3, 4?

Please accept our apologies for the lack of clarity in the methods section, and table 1. The pre-assembled cups were modelled as part of the modular implants and in two categories depending on the materials used for the head: the pre-assembled delta ceramic head category or the pre-assembled cobalt-chrome/stainless-steel head category. This latter group was not split because the related groups were not large enough to be considered on their own. We have now clarified the text in table 1 to mimic the labelling in table 3 and clarify the methods section as follows:
“In the case of the modular acetabular components, the exposure of interest is the liner, grouped as HCLPE, non-HCLPE, alumina, delta ceramic, or pre-assembled implant.”

Page 7, lines 15-17. Some factors tend to vary with time over two decades, for instance the risk of revision for infection. Did you consider adjusting for year of operation or stratified time periods? What about make of cup and stem? Poorly performing implants could skew the results, and such implants were maybe more frequent in the beginning of the study period?

Thank you for this comment. We have now further adjusted the analysed for year of primary surgery. This variable had no association with the considered outcomes, for both monobloc and modular acetabular components. The results and conclusions previously presented have been unchanged suggesting that the results shown have not changed over time. The analyses were adjusted for detailed stem and shells materials.

Table 1. DM cups were excluded. Still some 7-800 modular cups were cemented. What were these?

This is a registry and we have analysed the data as they were reported by the operating team. However, the components used were not those used in dual-mobility implants as double-checked by the surgical co-authors. The numbers are low; 674 procedures of nearly 650,000 modular hip replacements, and it is likely that a proportion of these do represent surgeons that have tried to use cemented non-DM cups. We cannot be absolutely sure of this, but even if these had been misclassified it is extremely unlikely that it would bias the results as it represents only 0.1% of the modular components.

Page 18, Line 23-25. Reference 31. The authors studied fractures, they did not report on dislocations.

Thank you for pointing this out – it was an error on our part. Previously a meta-analysis of RCTs generally comparing ceramic-on-ceramic bearings with metal-on-polyethylene bearings showed a lower late dislocation rate for the ceramic-on-ceramic group (<https://link.springer.com/article/10.1186/s13018-015-0163-2>), likely due to reduced sequalae of particulate wear debris as discussed above, likely attributable to the use of non-HCLPE cups in these older, long term studies included in that analysis. Rather than discuss HCLPE further, which has been covered extensively elsewhere and is now briefly included in the clinical explanation part of the discussion, we have instead removed this sentence.
Also I miss information on median follow-up for the study groups.
As previously discussed we used survival analysis to account for differences in follow-up time. Therefore whilst we agree that this would add an extra level of detail, we believe that the tables are already large and “heavy”, and adding such information would add minimum value to the manuscript compared to the risk of detracting from the key messages. For this reason we would prefer not too complexify the tables but would add this information if still deemed essential for the integrity of this research.

I am not familiar with all the tests that were used. It seems that the risks varied with time, and I do not know if the chosen tests handle non-proportionality properly. A statistical review will probably be called for.

The modelling framework used, the flexible survival analysis, is purposely designed to handle non-proportionality to allow appropriate comparisons at various post-operative time-points. We are absolutely certain that the proposed statistical modelling is the most appropriate method for the presented results to perform appropriate statistical testing/comparison in the context of non-proportionality.

REVIEWER#5:
This research study reports a cohort analysis of hip replacements reported in the UK NJR about the association of bearing surfaces materials with the risk of revision following primary total hip replacement. It is a very interesting topic of research since it contributes significantly to understanding the best option for the tribology of primary hip replacements. Moreover, this paper takes into consideration a remarkable amount of data that can be easily generalized. However, some minor changes are needed to be suitable for pubblication.

Introduction. This section can be shortened and focused more on the topic of research, that is the relationship between the tribology of the prosthesis and the risk of revision. Some lines do not give added value to the topic (ex: 3-4, 9-11, 21-23).

Thank you for highlighting this. We have removed lines 3-4.

Lines 9-11 have been kept as we feel it is important to highlight the potential crossover between reasons for revision and the underlying mechanism. As commented by reviewer 4, previously there was no discussion around the potential causative pathway between the association between bearing materials and risk of revision which we have now addressed by adding a paragraph to the discussion. This introductory sentence is of benefit to the reader as it links to this and describes the potential interplay between wear, debris, inflammatory reaction, loosening, low-grade infection, and instability as a prelude to our findings where all-cause and cause-specific revision risks were found to follow similar patterns in relation to bearing materials.

Our opinion is that lines 21-23 are crucial to the justification for the paper. Implant performance by broad bearing surface combination is already regularly reported in most national joint registry annual reports. The uniqueness of this analysis is that we have further subdivided these broad categories by making important distinctions within these groups (as well as performing adjusted statistical modelling including time-varying effects); this sentence provides examples of the subgroups used here which we feel is useful especially for the non-subspecialist reader.

We hope you accept our justification for these introductory points but are willing to remove them if you still feel they do not add enough value to justify their inclusion having considered our response.

M&M:
- Data source, line 6: numbers given are in conflict with what is described in tables. The total amount of replacements analysed according to tables is 1.027.098, monoblocs are 378.897 and modulars are 648.201. A comprehensive check of the numbers reported should be done throughout the manuscript.

We have checked all the numbers and amended the section in data source line 6, as well as and in sFigure 1. Please accept our apologies for this error.

- I was not able to find eFigure 1

Please accept our apologies for this error, corrected to sFigure 1.

- How was the reference group (cobalt chrome head + HCLPE cup) determined? There is no mention of the reference group in this section
For modular implants, those with a cobalt chrome head + HCLPE liner were the most commonly used in our observation period. For consistency, and because the modular implants are more common than the monobloc ones, we also selected this group as the reference for the monobloc implants. This is now described in the “Exposure and adjustment factors” sub-section of the methods section:

“Implants with a cobalt chrome head and a HCPLE liner or cup were used as the reference group.”

Results:
- Tables are difficult to follow. Highlighting significant positive results (lower risk of revision for "experimental groups") could be of help

Thank you for this recommendation. We have reordered the presentation of our findings to highlight those “positive” findings first and, hopefully, reduce the complexity of the results interpretation.

- "This lower revision rate was limited to the first year when a delta ceramic head 7 was paired with a delta ceramic or non-HCLPE liner" (page 14, lines 6-7), according to sTable18, lower revision rate is seen at least up to 2 years from surgery

This is indeed an error and we have now corrected the text:

“This lower revision rate was limited to the first two years when a delta ceramic head 7 was paired with a delta ceramic or non-HCLPE liner”.

- Page 14, lines 16-18. Heads and cups combinations other than the reference group have been compared. However, there is no mention in the text to the reason of this choice.

We have now expanded the methods section to describe the comparison conducted and removed the comparisons to the delta ceramic head and liner component as this construct had not the lowest risk of revision.

Discussion. This section is difficult to follow: a revision of the structure should be done. It could be better to summarize the results at first, then compare them to previous work already available in the Literature and at the end higlight limitations and strenght of the study. Moreover, some lines (17-19) should be included in M&M section rather than in the discussion.
We have now modified the discussion as recommended. We are very grateful for these comments as the manuscript, especially the discussion, is now much more impactful.
We have also moved the highlighted lines to the methods section.

-----------------------------------------------------------
General editorial requests:

1. Please upload any figures associated with your paper as individual TIF or EPS files with 300dpi resolution at resubmission; please read our figure guidelines for more information on our requirements: http://journals.plos.org/plosmedicine/s/figures. While revising your submission, please upload your figure files to the PACE digital diagnostic tool, https://pacev2.apexcovantage.com/. PACE helps ensure that figures meet PLOS requirements. To use PACE, you must first register as a user. Then, login and navigate to the UPLOAD tab, where you will find detailed instructions on how to use the tool. If you encounter any issues or have any questions when using PACE, please email us at PLOSMedicine@plos.org.

2. Please ensure that the paper adheres to the PLOS Data Availability Policy (see http://journals.plos.org/plosmedicine/s/data-availability), which requires that all data underlying the study's findings be provided in a repository or as Supporting Information. For data residing with a third party, authors are required to provide instructions with contact information for obtaining the data. PLOS journals do not allow statements supported by "data not shown" or "unpublished results." For such statements, authors must provide supporting data or cite public sources that include it.

3. We ask every co-author listed on the manuscript to fill in a contributing author statement, making sure to declare all competing interests. If any of the co-authors have not filled in the statement, we will remind them to do so when the paper is revised. If all statements are not completed in a timely fashion this could hold up the re-review process. If new competing interests are declared later in the revision process, this may also hold up the submission. Should there be a problem getting one of your co-authors to fill in a statement we will be in contact. YOU MUST NOT ADD OR REMOVE AUTHORS UNLESS YOU HAVE ALERTED THE EDITOR HANDLING THE MANUSCRIPT TO THE CHANGE AND THEY SPECIFICALLY HAVE AGREED TO IT. You can see our competing interests policy here: http://journals.plos.org/plosmedicine/s/competing-interests.

-----------------------------------------------------------

To submit your revised manuscript please use the following link:

https://www.editorialmanager.com/pmedicine/

Your article can be found in the "Submissions Needing Revision" folder.

__________________________________________________
In compliance with data protection regulations, you may request that we remove your personal registration details at any time. (Use the following URL: https://www.editorialmanager.com/pmedicine/login.asp?a=r). Please contact the publication office if you have any questions.
