# Supplementary material for: The association of bearing surface materials with the risk of revision following primary total hip replacement: A cohort analysis of 1,026,481 hip replacements from the National Joint Registry
Source: PLoS Med. 2024 Nov 7;21(11):e1004478. doi: 10.1371/journal.pmed.1004478 (PMC11542800; doi:10.1371/journal.pmed.1004478)
Supplement: Attachment — Submitted filename: Responses to comments 2.docx [file pmed.1004478.s004.docx]

Please find below our responses to the editorial/review comments.

Reviewer #5: **I have a minor revision to suggest. It could be better to include in the manuscript the reasons of the choice of the reference group. In the answers given it was clearly stated, but there is no reference in the main text. It could be helpful to better understand the comparisons even for non-subspecialist readers.**
Thank you; we have now added the following sentences to the method section:

“Implants with a cobalt chrome head and a HCPLE liner or cup were used as the reference group as this was the most commonly used implant bearing surface combination over the studied period. The combination remains amongst the most popular across numerous national health organisations including the NHS and the care organisations in the Nordic countries.”

Reviewer #6: **On the absence of addressing competing risks, the authors' rebuttal appears reasonable. It might be emphasized within the manuscript itself.**

Thank you; we have now added the following sentences to the method section:

This research focussed specifically on implant-survivorship i.e. net implant failure; therefore non-competing rather than competing-risk modelling was used ([Are competing risks models appropriate to describe implant failure? (tandfonline.com)](https://www.tandfonline.com/doi/pdf/10.1080/17453674.2018.1444876)).

COMMENTS FROM THE GUEST ACADEMIC EDITOR
Reviewer 5 was invited to act as the academic editor for your revised manuscript. She looked through all the reviewer comments as well as your revised paper. She wrote that she believed that your paper was well-written and tackled an interesting topic for the orthopaedic community. **She was keen for your paper to be taken forward to publication but asked if the authors could address the point she made as Reviewer 5, namely that you include a more include a more precise explanation regarding the reference group of comparisons in the main text, as she believes this would be beneficial for non-subspecialist readers.**

Thank you; we have now addressed this recommendation as described above.

EDITORIAL REQUESTS
Data Availability Statement (DAS):
Thank you for your detailed DAS. I have only 1 minor amendment to suggest; the first sentence contains the **phrase ‘…the data were analysed in a data safe heaven…’ – I think this is meant to read as ‘data safe haven’**. Could you revise this if appropriate please?

Please accept our apologies for this; corrected.

Reporting guidance:
Thank you for amending your STROBE checklist with reference to the sections rather than page numbers.
**Please include a statement in your Methods section of your main text stating ‘This study is reported as per the ‘Strengthening the Reporting of Observational Studies in Epidemiology (STROBE): guidelines for reporting observational studies’ Statement’ and also refer to the checklist’s location.**
Thank you; statement added.

Statistical reporting:
Thank you for revising your tables such that 95% Cis are separated by commas instead of hyphens. I note that, in some places, **CIs are still reported with a hyphen and not with a comma, e.g. in the Results section of the main text. We would appreciate it if you could review your entire document to check and revise this accordingly.**
Thank you; the manuscript has been updated accordingly.

Author summary:
Thank you for providing an author summary. **This will need to be revised to match the journal style, as below:**
Thank you; the summary has been modified accordingly:

Why Was This Study Done?

- The categorisation of hip implants used as part of hip replacements is typically broad in national hip arthroplasty registries and does not fully reflect the observed differences in revision risks associated within the different types of implant materials grouped together.
- This research aimed to report the risk of revision by the detailed implant materials used to help the surgical community, and therefore patients, identify those with the lowest risk of further surgery or revision which will improve shared decision making prior to surgery.

What Did the Researchers Do and Find?

- This research analysed 1,026,481 primary total hip replacements performed in England and Wales with information up to 15 years after these initial hip replacement operations.
- Hip prostheses with a delta ceramic or oxidised zirconium head and highly crosslinked polyethylene liner or cup had the lowest risk of revision throughout the 15 years following surgery.
- These findings were also found when investigating the specific reasons for revision hip replacements being performed.

What Do These Findings Mean?

- These results, from one of the largest registries in the world covering all public and private health care structures in England and Wales will help hospitals, surgeons and therefore patients to choose hip implants and combinations of them that can be used with the lowest risk of revision.
- These results are not from a randomised controlled trial and therefore it is impossible to control for all factors that can influence the risk of revision.
